# Supplementary material for: Dopamine Receptor Ligand Selectivity—An In Silico/In Vitro Insight
Source: Biomedicines. 2023 May 17;11(5):1468. doi: 10.3390/biomedicines11051468 (PMC10216180; doi:10.3390/biomedicines11051468)
Supplement: Supplementary file 1 [file biomedicines-11-01468-s001.zip › biomedicines-2374148-supplementary.pdf]

Dopamine Receptor Ligand Selectivity—An In Silico/In Vitro Insight – Supplementary information

Lukas Zell, Alina Bretl, Veronika Temml and Daniela Schuster

Table of content – supplementary information

1. Dataset assembly 1

2.1 Dataset of D_1R-selective compounds (ChEMBL) 1

2.2 Dataset of D_2R-selective compounds (ChEMBL) 3

2.3 Dataset of D_2like-selective compounds (ChEMBL) 5

2.4 Dataset of D_3R-selective compounds (ChEMBL) 17

2. Molecular Dynamics Simulation (MDS) – methodological overview 24

3. In vitro screening– overview of 2D structures and NDF values of all compounds 29

4. In silico assessment of DR subtype selectivity – a distance-based approach 36

4.1. ChEMBL validation 36

4.2. Retrospective analysis of investigated compounds 36

5. Molecular dynamics simulation (MDS) – a workflow for the D_2R 37

6. In vitro careening activity of selected compounds 37

6.1. Summary of in vitro activity (based on NDF values) of control compounds and selected virtual hits 37

6.2. Summary of Ki values determined in vitro – overview of DR subtype selectivity 42

7. In silico insights into the DR binding pockets 43

1. Dataset assembly

2.1 Dataset of D_1R-selective compounds (ChEMBL)

Table S1. D_1R-selective compounds extracted from ChEMBL with in vitro determined biological activities for all investigated DR subtypes.

ID	SMILES	Ki [nM][a]			Ref.
		D_1R	D_2R	D_3R	
**SC1**	O=C(C=1C([H])=C([H])C([F])=C([H])C=1[H])N([H])C([H])([H])C([H])([H])N2C([H])([H])C([H])([H])N(C([H])([H])C2([H])[H])[C@@]4([H])C=3C([H])=C([H])C([H])=C([H])C=3C([H])([H])C([H])([H])C4([H])[H]	70	3090	1570	[46]
**SC2**	N#CC=1C([H])=C([H])C2=C(C=1[H])C(=C([H])N2[H])C([H])([H])N4C([H])([H])C([H])([H])N(C=3C([H])=C([H])C([Cl])=C([H])C=3[H])C([H])([H])C4([H])[H]	550	4300	6400	[47]
**SC3**	N#CC=2C([H])=C([H])C=1C([H])=C(N([H])C=1C=2[H])C([H])([H])N4C([H])([H])C([H])([H])N(C=3C([H])=C([H])C([Cl])=C([Cl])C=3[H])C([H])([H])C4([H])[H]	880	2000	33000	
**SC4**	[Br]C=1C([H])=C([H])C(OC([H])([H])[H])=C(C=1[H])C2=N[C@]([H])([C@]([H])(O2)C([H])([H])[H])C([H])([H])N4C([H])([H])C([H])([H])N(C=3C([H])=C([H])C([H])=C([H])C=3[H])C([H])([H])C4([H])[H]	480	21000	10000	[48]
**SC5**	[Cl]C=1C([H])=C([H])C(=C([H])C=1[H])N2C([H])([H])C([H])([H])N(C([H])([H])C2([H])[H])C([H])([H])C=3C([H])=NN4C=3C([H])=C([H])C([H])=C4C#C[H]	810	3700	4600	[49]
**SC6**	[H]C=1C([H])=C([H])C(=C([H])C=1[H])C([H])([H])C=2C([H])=C([H])C([H])=C([H])C=2OC([H])([H])C([H])([H])N(C([H])([H])[H])C([H])([H])[H]	343	2740	10000	[50]
**SC7**	[H]C=1C([H])=C([H])C(=C([H])C=1[H])C([H])([H])C=2C([H])=C([H])C([H])=C([H])C=2OC([H])([H])C([H])([H])C([H])([H])N3C([H])([H])C([H])([H])C([H])([H])C3([H])[H]	113	1006	2923	
**SC8**	[H]C=1C([H])=C([H])C(=C([H])C=1[H])C([H])([H])C=2C([H])=C([H])C([H])=C([H])C=2OC([H])([H])C([H])([H])N3C([H])([H])C([H])([H])C([H])([H])C([H])([H])C3([H])[H]	33.7	1015	7804	
**SC9**	[H]C=1C([H])=C([H])C(=C([H])C=1[H])C([H])([H])C=2C([H])=C([H])C([H])=C([H])C=2OC([H])([H])C([H])([H])N3C([H])([H])C([H])([H])OC([H])([H])C3([H])[H]	201	10000	10000	
**SC10**	[H]C=1C([H])=C([H])C(=C([H])C=1[H])C([H])([H])C=2C([H])=C([H])C([H])=C([H])C=2OC([H])([H])C([H])([H])N3C([H])([H])C([H])([H])C([H])([H])C3([H])[H]	379	10000	1818	
**SC11**	O=C2C1=C(C([H])=C([Cl])C([H])=C1[Cl])N([H])C(=O)[C@@]2(C=4C([H])=C([H])C([H])=C(OC([H])([H])C=3C([H])=C([H])C([H])=C([H])C=3[H])C=4[H])C([H])([H])[H]	120	10000	10000	[51]
**SC12**	[H]C=1C([H])=C([H])C3=C(C=1[H])C([H])([H])C=2C([H])=C(OC([H])([H])[H])C([H])=C([H])C=2C([H])([H])C([H])([H])N(C([H])([H])C([H])([H])C3([H])[H])C([H])([H])[H]	137	1396	23903	[52]
**SC13**	[H]C=1C([H])=C([H])C3=C(C=1[H])C([H])([H])C2=C([H])C(OC([H])([H])[H])=C(OC([H])([H])[H])C([H])=C2C([H])([H])C([H])([H])N(C([H])([H])C([H])([H])C3([H])[H])C([H])([H])[H]	579	1028	14830	
**SC14**	O=C1C3=C(N=C(N1C([H])([H])[H])N2C([H])([H])C([H])([H])OC([H])([H])C2([H])[H])C(=C([H])N3[H])C([H])([H])N5C([H])([H])C([H	520	1100	2700	[53]

ID	SMILES	Ki [nM][a]			Ref.
		D_1R	D_2R	D_3R	
	])([H])N(C=4C([H])=C([H])C([H])=C([H])C=4[H])C([H])([H])C5([H])[H]				
**SC15**	O=C(C=1C([H])=C([H])C([F])=C([H])C=1[H])C([H])([H])C([H])([H])C([H])([H])N5[C@@]3([H])[C@]7([H])[C@@]2([H])C([H])([H])[C@]6([H])[C@]4([H])[C@@]2([H])[C@@]3([H])[C@]4([H])[C@@]5(O[H])[C@]67[H]	209	1724	1958	[54]
**SC16**	[H]C=1C([H])=C([H])C2=C(C=1[H])C4=C(N2[H])C([H])([H])C=3C([H])=C([H])C([H])=C([H])C=3C([H])([H])C([H])([H])N([C@@]([H])(C4([H])[H])C([H])([H])[H])C([H])([H])[H]	640	10000	10000	[55]
**SC17**	[H]C=1C([H])=C([H])C2=C(C=1[H])C4=C(N2[H])C([H])([H])C=3C([H])=C([H])C([H])=C([H])C=3C([H])([H])C([H])([H])N([C@@]([H])(C4([H])[H])C([H])([H])O[H])C([H])([H])[H]	114	2923	4676	
**SC18**	O=C2C=1C([H])=C([H])C([H])=C([H])C=1C([H])([H])[C@@]2([H])C([H])([H])C([H])([H])N4C([H])([H])C([H])([H])N(C=3C([H])=C([H])C([Cl])=C([H])C=3[H])C([H])([H])C4([H])[H]	389.05	10000	4534	[56]
**SC19**	[Cl]C=1C([H])=C([H])C(=C([H])C=1[H])N2C([H])([H])C([H])([H])C([H])([H])N(C([H])([H])C2([H])[H])C([H])([H])C([H])([H])C([H])([H])C=4OC=3C([H])=C([H])C([H])=C([H])C=3C=4[H]	274	2507	1462	
**SC20**	[H]C=1C([H])=C([H])C(=C([H])C=1[H])[C@@]3([H])C=2C([H])=C(O[H])C([H])=C([H])C=2C([H])([H])C([H])([H])N(C3([H])[H])C([H])([H])[H]	207	1000	1000	[57]
**SC21**	[H]C=1C([H])=C([H])C(=C([H])C=1[H])[C@@]3([H])C=2C([H])=C(O[H])C(=C([H])C=2C([H])([H])C([H])([H])N(C3([H])[H])C([H])([H])[H])N([H])[H]	334	1000	1000	
**SC22**	[H]C=1C([H])=C([H])C(=C([H])C=1[H])[C@]3([H])C2=C([H])C(O[H])=C(C([H])=C2C([H])([H])C([H])([H])N(C3([H])[H])C([H])([H])[H])C4=C([H])C([H])=C([H])C(=C4[H])C([H])([H])[H]	216	1000	1000	
**SC23**	[H]C=1C([H])=C([H])C(=C([H])C=1[H])[C@@]3([H])C=2C([H])=C(O[H])C(=C([H])C=2C([H])([H])C([H])([H])N(C3([H])[H])C([H])([H])[H])C=4C([H])=C([H])C(O[H])=C([H])C=4[H]	349	1000	1000	
**SC24**	[H]/C4=C3\C1=C2/C(=C(/[H])C(OC([H])([H])[H])=C1OC([H])([H])[H])C([H])([H])C([H])([H])N([C@@]2([H])C([H])([H])C3=C([H])C(O[H])=C4OC([H])([H])[H])C([H])([H])[H]	373	inactive	>950	[58]
**SC25**	[H]/C1=C(\[H])C(OC([H])([H])[H])=C(OC([H])([H])[H])C4=C1C([H])([H])[C@@]3([H])C2=C([H])C(OC([H])([H])[H])=C(OC([H])([H])[H])C([H])=C2C([H])([H])C([H])([H])N3C4([H])[H]	153	1125	1371	[59]
**SC26**	[H]/C3=C(\[H])C(O/C2=N/C([H])=C([H])C=1OC([H])=C([H])C=12)=C([H])C(=C3/C5=C(/N=C(/[H])C4=NC([H])=C([H])N45)C([H])([H])[H])C([H])([H])[H]	110	8300	7900	[60]
**SC27**	[F]C([F])([F])C=3C([H])=C(O/C2=N/C([H])=C([H])C=1C([H])=C([H])[S]C=12)C([H])=C([H])C=3/C5=C(\[H])C([H])=C([H])C4=NC([H])=C([H])N45	130	8700	6200	
**SC28**	[F]C([F])([F])C=3C([H])=C(O/C2=N/C([H])=C([H])C=1OC([H])=C([H])C=12)C([H])=C([H])C=3/C5=C(\[H])C([H])=C([H])C4=NC([H])=C([H])N45	150	7600	10000	

ID	SMILES	Ki [nM][a]			Ref.
		D1R	D2R	D3R	
**SC29**	[F]C([F])([F])C=3C([H])=C(O/C2=N/C([H])=C([H])C=1OC([H])=C([H])C=12)C([H])=C([H])C=3/C5=C(/N=C(/[H])C4=NC([H])=C([H])N45)C([H])([H])[H]	140	9600	8100	

[a]All KI values retrieved from ChEMBL were determined in vitro utilizing radio-ligand binding assays.

2.2 Dataset of D2R-selective compounds (ChEMBL)

Table S2. D2R-selective compounds extracted from ChEMBL with in vitro determined biological activities for all investigated DR subtypes.

ID	SMILES	Ki [nM][a]			Ref.
		D1R	D2R	D3R	
**SC30**	[H]C=3C([H])=C2C1=C(/N=C(/[H])N1C([H])([H])[C@]([H])(N([H])C([H])([H])[H])C2([H])[H])C=3[H]	low affinity	355	2459	[61]
**SC31**	O=C(/C1=C(\[H])C([Cl])=C(C([H])=C1OC([H])([H])[H])N([H])C([H])([H])[H])N([H])[C@@]2([H])C([H])([H])C([H])([H])C([H])([H])N(C2([H])[H])C([H])([H])C=3C([H])=C([H])C([H])=C([H])C=3[H]	low affinity	12	1929	
**SC32**	O=C(C=1C([H])=C([Cl])C(=C([H])C=1OC([H])([H])[H])N(C(=O)C([H])([H])[H])C([H])([H])[H])N([H])C([H])([H])[C@]2([H])C([H])([H])C([H])([H])N(C2([H])[H])C([H])([H])C=3C([H])=C([H])C([H])=C([H])C=3[H]	5100	110	7200	[62]
**SC33**	O=C(C=1C([H])=C([Cl])C(=C([H])C=1OC([H])([H])[H])N([H])[H])N([H])C([H])([H])[C@@]2([H])C([H])([H])C([H])([H])N(C2([H])[H])C([H])([H])C=3C([H])=C([H])C([H])=C([H])C=3[H]	11000	820	2200	
**SC34**	O=C1OC([H])([H])C([H])([H])N1C([H])([H])C([H])([H])N6C([H])([H])C([H])([H])C([H])(C2=C([H])N(C=3C([H])=C([H])C(=C([H])C2=3)C=4N=NN(N=4)C([H])([H])[H])C=5C([H])=C([H])C([F])=C([H])C=5[H])C([H])([H])C6([H])[H]	1250	610	1190	[63]
**SC35**	[H]C=1/C(=C(/[H])N2C=1C([H])([H])[C@]([H])(N(C([H])([H])C([H])([H])C([H])([H])[H])C([H])([H])C([H])([H])C([H])([H])[H])C([H])([H])C2([H])[H])C([H])([H])[H]	83000	800	1300	[64]
**SC36**	O=C(C=1C([H])=C([Cl])C(=C([H])C=1OC([H])([H])[H])N([H])[H])N([H])[C@@]2([H])C([H])([H])C([H])([H])N(C([H])([H])[C@]2([H])C([H])([H])[H])C([H])([H])C=3C([H])=C([H])C([H])=C([H])C=3[H]	19000	470	2200	[65]
**SC37**	[H]C=1C([H])=C([H])C(OC([H])([H])[H])=C(C=1[H])N2C([H])([H])C([H])([H])N(C([H])([H])C2([H])[H])C([H])([H])C=4C=3C([H])=NC(=NC=3N([H])C=4C([H])([H])[H])N(C([H])([H])[H])C([H])([H])[H]	5600	250	1500	
**SC38**	[H]C=1C([H])=C([H])C(OC([H])([H])[H])=C(C=1[H])N2C([H])([H])C([H])([H])N(C([H])([H])C2([H])[H])C([H])([H])C=4N=C3/C(=C(/[H])C(=C([H])N3C=4[H])C([H])([H])[H])N5/N=C(/[H])C([H])=C5[H]	8700	530	2400	[66]
**SC39**	[H]C=1C([H])=C([H])C(OC([H])([H])[H])=C(C=1[H])N2C([H])([H])C([H])([H])N(C([H])([H])C2([H])[H])C([H])([H])C=4N=C3/C(=C(/[H])C(=C([H])N3C=4[H])C([H])([H])[H])N5C([H])([H])C([H])([H])OC([H])([H])C5([H])[H]	6400	210	2100	

ID	SMILES	Ki [nM][a]			Ref.
		D_1R	D_2R	D_3R	
**SC40**	[H]C=1C([H])=C([H])C(OC([H])([H])[H])=C(C=1[H])N2C([H])([H])C([H])([H])N(C([H])([H])C2([H])[H])C([H])([H])C=4N=C3C([H])=C([H])C([H])=C(N3C=4[H])N5/N=C(/[H])C([H])=C5[H]	27000	590	1000	
**SC41**	[H]C=1C([H])=C([H])C(OC([H])([H])[H])=C(C=1[H])N2C([H])([H])C([H])([H])N(C([H])([H])C2([H])[H])C([H])([H])C=4N=C3C([H])=C([H])C([H])=C([H])N3C=4[H]	20000	990	3900	
**SC42**	[Cl]/C2=N/N1C([H])=C(N=C1C([H])=C2[H])C([H])([H])N4C([H])([H])C([H])([H])N(C=3C([H])=C([H])C([H])=C([H])C=3OC([H])([H])[H])C([H])([H])C4([H])[H]	7700	96	1400	
**SC43**	[F]C([H])([H])C([H])([H])OC=1C([H])=C([H])C([H])=C(C=1[H])C([H])([H])N3C([H])([H])C([H])([H])N(C=2C([H])=C([H])C([Cl])=C([H])C=2[H])C([H])([H])C3([H])[H]	5100	590	1100	[67]
**SC44**	[F]C([H])([H])C([H])([H])OC=1C([H])=C([H])C(=C([H])C=1OC([H])([H])[H])C([H])([H])N3C([H])([H])C([H])([H])N(C=2C([H])=C([H])C([H])=C([H])C=2OC([H])([H])[H])C([H])([H])C3([H])[H]	5400	310	1100	[67]
**SC45**	[F]C([H])([H])C([H])([H])O/C1=C(\[H])C(OC([H])([H])[H])=C(OC([H])([H])[H])C([H])=C1C([H])([H])N3C([H])([H])C([H])([H])N(C=2C([H])=C([H])C([Cl])=C([H])C=2[H])C([H])([H])C3([H])[H]	3500	760	3900	[67]
**SC46**	O=C1N([H])C([H])([H])C([H])([H])N1C([H])([H])C([H])([H])N6C([H])([H])C([H])([H])C([H])(C2=C([H])N(C=3C([H])=C([H])C(=C([H])C2=3)C=4/N=C(/[H])N(N=4)C([H])([H])[H])C=5C([H])=C([H])C([F])=C([H])C=5[H])C([H])([H])C6([H])[H]	4100	660	2900	[68]
**SC47**	O=C1N(C([H])([H])C([H])([H])[C@]1([H])C([H])([H])C([H])([H])N6C([H])([H])C([H])([H])[C@@]([H])(C2=C([H])N(C=3C([H])=C([H])C(=C([H])C2=3)C=4/N=C(/[H])N(N=4)C([H])([H])[H])C=5C([H])=C([H])C([F])=C([H])C=5[H])C([H])([H])C6([H])[H])C([H])([H])[H]	2600	190	1400	[68]
**SC48**	[Br]C=1C([H])=C([H])C(OC([H])([H])[H])=C(C=1[H])C2=N/C(=C(/[H])[S]2)C([H])([H])N4C([H])([H])C([H])([H])N(C=3C([H])=C([H])C([H])=C([H])C=3[H])C([H])([H])C4([H])[H]	6400	190	20000	[53]
**SC49**	[H]C=3C([H])=C2C1=C(/N=C(/O[H])N1C([H])([H])[C@]([H])(N(C([H])([H])[H])C([H])([H])[H])C2([H])[H])C=3[H]	5000	20	10000	[69]
**SC50**	[F]C([H])([H])C([H])([H])C([H])([H])OC=1C([H])=C([H])C(=C([H])C=1[H])N2N=N/C(=C2/[H])C([H])([H])N4C([H])([H])C([H])([H])N(C=3C([H])=C([H])C([H])=C([H])C=3OC([H])([H])[H])C([H])([H])C4([H])[H]	38000	340	2800	[70]
**SC51**	[F]C([H])([H])C([H])([H])OC([H])([H])C([H])([H])C([H])([H])OC=1C([H])=C([H])C(=C([H])C=1[H])N2N=N/C(=C2/[H])C([H])([H])N4C([H])([H])C([H])([H])N(C=3C([H])=C([H])C([H])=C([H])C=3OC([H])([H])[H])C([H])([H])C4([H])[H]	3600	440	2900	[70]
**SC52**	O=C(C=1C([H])=C([H])C([F])=C([H])C=1[H])C([H])([H])C([H])([H])C(=O)N3C([H])([H])C([H])([H])C(O[H])(C=2C([H])=C([H])C([Cl])=C([H])C=2[H])C([H])([H])C3([H])[H]	19000	860	1700	[71]
**SC53**	[H]C=1C([H])=C([H])C2=C(C=1[H])C([H])([H])C([H])(C2([H])[H])C([H])([H])N5C([H])([H])C([H])([H])N(C=4C([H])=C([H])C=3OC([H])([H])C([H])([H])OC=3C=4[H])C([H])([H])C5([H])[H]	3000	738	2840	[72]

ID	SMILES	Ki [nM][a]			Ref.
		D_1R	D_2R	D_3R	
**SC54**	[Cl]C=1C([H])=C([H])C(=C([H])C=1[H])N2C([H])([H])C([H])([H])N(C([H])([H])C2([H])[H])C([H])([H])C3=C([H])N([H])C=4/N=C(/[H])C([H])=C([H])C3=4	10000	960	2310	

[a]All KI values retrieved from ChEMBL were determined in vitro utilizing radio-ligand binding assays.

2.3 Dataset of D_2like-selective compounds (ChEMBL)

Table S3. D_2like-selective compounds extracted from ChEMBL with in vitro determined biological activities for all investigated DR subtypes.

ID	SMILES	Ki [nM][a]			Ref.
		D_1R	D_2R	D_3R	
**SC55**	[H]C=1C([H])=C([H])C(=C([H])C=1[H])C2=C([H])C([H])([H])N(C([H])([H])C2([H])[H])C([H])([H])C([H])([H])C#CC=3C([H])=C([H])C([H])=NC=3[H]	5004	3.3	20	[73]
**SC56**	[H]C=1C([H])=C([H])C(=C([H])C=1[H])C2=C([H])C([H])([H])N(C([H])([H])C2([H])[H])C([H])([H])C([H])([H])C#CC=3C([H])=C([H])C(=C([H])C=3[H])N([H])[H]	10000	0.67	24	
**SC57**	[H]C=1C([H])=C([H])C(=C([H])C=1[H])C2=C([H])C([H])([H])N(C([H])([H])C2([H])[H])C([H])([H])C([H])([H])C#CC=3C([H])=C([H])C(=NC=3[H])N([H])[H]	10000	19	7	
**SC58**	[H]C=1C([H])=C([H])C(=C([H])C=1[H])C2=C([H])C([H])([H])N(C([H])([H])C2([H])[H])C([H])([H])C([H])([H])C#C/C3=N/C([H])=C(C([H])=C3[H])N([H])[H]	10000	1.9	2.6	
**SC59**	O=C(C=3C([H])=C([H])C=2C=1C([H])=C([H])C([H])=C([H])C=1C([H])([H])C=2C=3[H])N([H])C([H])([H])C([H])([H])C([H])([H])C([H])([H])N5C([H])([H])C([H])([H])N(C=4C([H])=C([H])C([H])=C([Cl])C=4[Cl])C([H])([H])C5([H])[H]	10000	217	1.4	[74]
**SC60**	O=C(C=3C([H])=C([H])C=2C=1C([H])=C([H])C([H])=C([H])C=1C=2C=3[H])N([H])C([H])([H])C([H])([H])C([H])([H])C([H])([H])N5C([H])([H])C([H])([H])N(C=4C([H])=C([H])C([H])=C([Cl])C=4[Cl])C([H])([H])C5([H])[H]	10000	262	0.9	
**SC61**	[Cl]C=2C([H])=C([H])C=1OC([H])([H])[C@@]([H])(OC=1C=2[H])C([H])([H])N([H])C([H])([H])[C@@]4([H])C([H])([H])C([H])([H])N(C=3C([H])=C([H])C([H])=C([H])C=3OC([H])([H])[H])C([H])([H])C4([H])[H]	1000	13.2	1.6	[75]
**SC62**	[Cl]C=1C([H])=C([H])C(=C([H])C=1[H])[C@@]3(O[H])C([H])([H])[C@@]2([H])N([C@]([H])(C([H])([H])C2([H])[H])C3([H])[H])C([H])([H])C4=C([H])N([H])C=5C([H])=C([H])C([H])=C([H])C4=5	1220	33.4	15.5	[76]
**SC63**	[Cl]C=1C([H])=C([H])C(=C([H])C=1[H])[C@@]3(O[H])C([H])([H])[C@@]2([H])N([C@]([H])(C([H])([H])C2([H])[H])C3([H])[H])C([H])([H])C4=C([H])[S]C=5C([H])=C([H])C([H])=C([H])C4=5	3000	12.9	3.62	
**SC64**	[Cl]C=1C([H])=C([H])C(=C([H])C=1[H])[C@@]3(O[H])C([H])([H])[C@@]2([H])N([C@]([H])(C([H])([H])C2([H])[H])C3([H])[H])C([H])([H])C4=C([H])OC=5C([H])=C([H])C([H])=C([H])C4=5	3930	1.06	0.71	
**SC65**	O=C1C([H])=NN(C(=O)N1C([H])([H])[H])C([H])([H])C([H])([H])C([H])([H])C([H])([H])N4C([H])([H])C([H])([H])N(/C3=C(\[H])C([H])=C([H])C=2C([H])=C([H])C(OC([H])([H])[H])=C([H])C=23)C([H])([H])C4([H])[H]	7396	22.01	62.8	[77]

ID	SMILES	Ki [nM][a]			Ref.
		D1R	D2R	D3R	
**SC66**	O=C(C=1C([H])=C3C([H])=C([H])C=1C([H])([H])C([H])([H])C=2C([H])=C([H])C(=C([H])C=2[H])C([H])([H])C3([H])[H])N([H])C([H])([H])C([H])([H])C([H])([H])C([H])([H])N5C([H])([H])C([H])([H])N(C=4C([H])=C([H])C([H])=C([Cl])C=4[Cl])C([H])([H])C5([H])[H]	2800	220	3.6	[78]
**SC67**	O=C(C=2OC=1C([H])=C([H])C([H])=C([H])C=1C=2[H])N([H])C([H])([H])C([H])([H])C([H])([H])C([H])([H])N4C([H])([H])C([H])([H])N(C=3C([H])=C([H])C([H])=C([H])C=3OC([H])([H])[H])C([H])([H])C4([H])[H]	2000	36.5	0.92	
**SC68**	O=C(C=2OC=1C([H])=C([H])C([H])=C([H])C=1C=2[H])N([H])C([H])([H])C([H])([H])C([H])([H])C([H])([H])N4C([H])([H])C([H])([H])N(C=3C([H])=C([H])C([H])=C([Cl])C=3[Cl])C([H])([H])C4([H])[H]	2100	44.8	0.81	
**SC69**	O=C(/C2=C(\[H])C=1C([H])=C([H])C([H])=C([H])C=1N2[H])N([H])C([H])([H])C([H])([H])[C@@]([H])(O[H])C([H])([H])N4C([H])([H])C([H])([H])N(C=3C([H])=C([H])C([H])=C([Cl])C=3[Cl])C([H])([H])C4([H])[H]	4630	502	1.39	
**SC70**	O=C(/C2=C(\[H])C=1C([H])=C([H])C([H])=C([H])C=1N2[H])N([H])C([H])([H])C([H])([H])[C@@]([H])(O[H])C([H])([H])N4C([H])([H])C([H])([H])N(C=3C([H])=C([H])C([H])=C([Cl])C=3[Cl])C([H])([H])C4([H])[H]	10000	433	1.12	
**SC71**	O=C(/C2=C(\[H])C=1C([H])=C([H])C([H])=C([H])C=1N2[H])N([H])C([H])([H])C([H])([H])[C@]([H])(O[H])C([H])([H])N4C([H])([H])C([H])([H])N(C=3C([H])=C([H])C([H])=C([Cl])C=3[Cl])C([H])([H])C4([H])[H]	10000	715	16.6	
**SC72**	O=C(/C2=C(\[H])C=1C([H])=C([H])C([H])=C([H])C=1N2[H])N([H])C([H])([H])C([H])([H])[C@@]([H])(O[H])C([H])([H])N4C([H])([H])C([H])([H])N(C=3C([H])=C([H])C([H])=C([H])C=3OC([H])([H])[H])C([H])([H])C4([H])[H]	10000	249	1.4	[79]
**SC73**	O=C(/C2=C(\[H])C=1C([H])=C([H])C([H])=C([H])C=1N2[H])N([H])C([H])([H])C([H])([H])[C@@]([H])(O[H])C([H])([H])N4C([H])([H])C([H])([H])N(C=3C([H])=C([H])C([H])=C([H])C=3OC([H])([H])C([H])([H])C([H])([H])[H])C([H])([H])C4([H])[H]	4960	47.1	62.1	
**SC74**	O=C(/C2=C(\[H])C=1C([H])=C([F])C([H])=C([H])C=1N2[H])N([H])C([H])([H])C([H])([H])[C@]([H])(O[H])C([H])([H])N4C([H])([H])C([H])([H])N(C=3C([H])=C([H])C([H])=C([Cl])C=3[Cl])C([H])([H])C4([H])[H]	10000	293	1.61	
**SC75**	O=C(/C2=C(\[H])C=1C([H])=C([F])C([H])=C([H])C=1N2[H])N([H])C([H])([H])C([H])([H])[C@@]([H])(O[H])C([H])([H])N4C([H])([H])C([H])([H])N(C=3C([H])=C([H])C([H])=C([H])C=3OC([H])([H])[H])C([H])([H])C4([H])[H]	5060	244	2.41	
**SC76**	O=C(/C2=C(\[H])C=1C([H])=C([I])C([H])=C([H])C=1N2[H])N([H])C([H])([H])C([H])([H])[C@@]([H])(O[H])C([H])([H])N4C([H])([H])C([H])([H])N(C=3C([H])=C([H])C([H])=C([H])C=3OC([H])([H])[H])C([H])([H])C4([H])[H]	10000	520	3.6	
**SC77**	O=C(/C2=C(\[H])C=1C([H])=C(OC([H])([H])[H])C([H])=C([H])C=1N2[H])N([H])C([H])([H])C([H])([H])[C@@]([H])(O[H])C([H])([H])N4C([H])([H])C([H])([H])N(C=3C([H])=C([H])C([H])=C([Cl])C=3[Cl])C([H])([H])C4([H])[H]	10000	489	1.2	

ID	SMILES	Ki [nM][a]			Ref.
		D1R	D2R	D3R	
**SC78**	O=C(/C2=C(\[H])C=1C([H])=C(OC([H])([H])[H])C([H])=C([H])C=1N2[H])N([H])C([H])([H])C([H])([H])[C@@]([H])(O[H])C([H])([H])N4C([H])([H])C([H])([H])N(C=3C([H])=C([H])C([H])=C([H])C=3OC([H])([H])[H])C([H])([H])C4([H])[H]	10000	390	2.32	
**SC79**	O=C(C=2OC=1C([H])=C([H])C([H])=C([H])C=1C=2[H])N([H])C([H])([H])C([H])([H])[C@]([H])(O[H])C([H])([H])N4C([H])([H])C([H])([H])N(C=3C([H])=C([H])C([H])=C([Cl])C=3[Cl])C([H])([H])C4([H])[H]	10000	622	6.11	
**SC80**	O=C(C=2OC=1C([H])=C([H])C([H])=C([H])C=1C=2[H])N([H])C([H])([H])C([H])([H])[C@@]([H])(O[H])C([H])([H])N4C([H])([H])C([H])([H])N(C=3C([H])=C([H])C([H])=C([H])C=3OC([H])([H])[H])C([H])([H])C4([H])[H]	10000	507	7.51	
**SC81**	O=C(C=2OC=1C([H])=C([H])C([I])=C([H])C=1C=2[H])N([H])C([H])([H])C([H])([H])[C@]([H])(O[H])C([H])([H])N4C([H])([H])C([H])([H])N(C=3C([H])=C([H])C([H])=C([Cl])C=3[Cl])C([H])([H])C4([H])[H]	10000	581	7.71	
**SC82**	O=C(C=2OC=1C([H])=C([H])C([I])=C([H])C=1C=2[H])N([H])C([H])([H])C([H])([H])[C@@]([H])(O[H])C([H])([H])N4C([H])([H])C([H])([H])N(C=3C([H])=C([H])C([H])=C([H])C=3OC([H])([H])[H])C([H])([H])C4([H])[H]	3690	430	7.5	
**SC83**	O=C(C=2[S]C=1C([H])=C([H])C([H])=C([H])C=1C=2[H])N([H])C([H])([H])C([H])([H])[C@@]([H])(O[H])C([H])([H])N4C([H])([H])C([H])([H])N(C=3C([H])=C([H])C([H])=C([H])C=3OC([H])([H])[H])C([H])([H])C4([H])[H]	6650	337	4.6	
**SC84**	O=C(/C2=C(\[H])C=1C([H])=C([H])C([H])=C([H])C=1N2[H])N([H])C([H])([H])[C@]([H])(O[H])C([H])([H])C([H])([H])N4C([H])([H])C([H])([H])N(C=3C([H])=C([H])C([H])=C([Cl])C=3[Cl])C([H])([H])C4([H])[H]	10000	28.4	0.26	
**SC85**	O=C(C=2[S]C=1C([H])=C([H])C([H])=C([H])C=1C=2[H])N([H])C([H])([H])C([H])=C([H])C([H])([H])N4C([H])([H])C([H])([H])N(C=3C([H])=C([H])C([H])=C([Cl])C=3[Cl])C([H])([H])C4([H])[H]	1730	149	1.11	
**SC86**	O=C(C=2OC=1C([H])=C([H])C([H])=C([H])C=1C=2[H])N([H])C([H])([H])C([H])=C([H])C([H])([H])N4C([H])([H])C([H])([H])N(C=3C([H])=C([H])C([H])=C([Cl])C=3[Cl])C([H])([H])C4([H])[H]	1460	76.5	2.11	
**SC87**	O=C2C=1C([H])=C([H])C([H])=C([H])C=1C([H])([H])C([H])([H])C([H])([H])N2C([H])([H])C([H])([H])C([H])([H])C([H])([H])N4C([H])([H])C([H])([H])N(C=3C([H])=C([H])C([H])=C([Cl])C=3[Cl])C([H])([H])C4([H])[H]	1737.8	36.3078	14.45	[80]
**SC88**	O=C2C=1C([H])=C([H])C([H])=C([H])C=1C([H])([H])C([H])([H])C([H])([H])N2C([H])([H])C([H])([H])C([H])([H])C([H])([H])N4C([H])([H])C([H])([H])N(C=3C([H])=C([H])C([H])=C([H])C=3OC([H])([H])[H])C([H])([H])C4([H])[H]	2691.53	3.63078	1.585	
**SC89**	O=C(C=1C([H])=C3C([H])=C([H])C=1C([H])([H])C([H])([H])C=2C([H])=C([H])C(=C([H])C=2[H])C([H])([H])C3([H])[H])C([H])([H])C([H])([H])C([H])([H])N5C([H])([H])C([H])([H])C(O[H])(C=4C([H])=C([H])C([Cl])=C([H])C=4[H])C([H])([H])C5([H])[H]	9800	21	17	[81]

ID	SMILES	Ki [nM][a]			Ref.
		D_1R	D_2R	D_3R	
**SC90**	O=C(C=2[S]C=1C([H])=C([H])C([H])=C([H])C=1C=2[H])N([H])C([H])([H])C([H])([H])C([H])([H])C([H])([H])N6C([H])([H])C([H])([H])N(C=3C([H])=C5C([H])=C([H])C=3C([H])([H])C([H])([H])C=4C([H])=C([H])C(=C([H])C=4[H])C([H])([H])C5([H])[H])C([H])([H])C6([H])[H]	4000	83	1.6	
**SC91**	O=C(C=2[S]C=1C([H])=C([H])C([H])=C([H])C=1C=2[H])N([H])C([H])([H])C([H])([H])C([H])([H])C([H])([H])N4C([H])([H])C([H])([H])N(C=3C([H])=C(C([H])=C([H])C=3C([H])([H])[H])C([H])([H])[H])C([H])([H])C4([H])[H]	2400	84	2.5	
**SC92**	O=C(C=1C([H])=C(C([H])=C([H])C=1C([H])([H])[H])C([H])([H])[H])N([H])C([H])([H])C([H])([H])C([H])([H])C([H])([H])N3C([H])([H])C([H])([H])N(C=2C([H])=C([H])C([H])=C([H])C=2OC([H])([H])[H])C([H])([H])C3([H])[H]	2100	22	4.7	
**SC93**	O=C(C([H])=C([H])C=1C([H])=C([H])C([F])=C([H])C=1[H])N([H])C([H])([H])C([H])([H])C([H])([H])C([H])([H])N3C([H])([H])C([H])([H])N(C=2C([H])=C([H])C([H])=C([H])C=2OC([H])([H])[H])C([H])([H])C3([H])[H]	1100	27	0.34	[82]
**SC94**	[F]C([H])([H])C([H])([H])C([H])([H])OC=3C([H])=C2C1=C(C([H])=C([H])C(O[H])=C1O[H])C([H])([H])[C@]4([H])C2=C(C=3[H])C([H])([H])C([H])([H])N4C([H])([H])C([H])([H])C([H])([H])[H]	5000	28	430	
**SC95**	[F]C([H])([H])C([H])([H])C([H])([H])OC=3C([H])=C2C1=C(C([H])=C([H])C([H])=C1O[H])C([H])([H])[C@]4([H])C2=C(C=3[H])C([H])([H])C([H])([H])N4C([H])([H])C([H])([H])C([H])([H])[H]	5000	0.54	100	
**SC96**	[F]C([H])([H])C([H])([H])OC=3C([H])=C2C1=C(C([H])=C([H])C([H])=C1O[H])C([H])([H])[C@]4([H])C2=C(C=3[H])C([H])([H])C([H])([H])N4C([H])([H])C([H])([H])C([H])([H])[H]	5000	3.5	410	[69]
**SC97**	[F]C([H])([H])C([H])([H])C([H])([H])OC=3C([H])=C2C1=C(C([H])=C([H])C([H])=C1O[H])C([H])([H])[C@]4([H])C2=C(C=3[H])C([H])([H])C([H])([H])N4C([H])([H])C([H])([H])[H]	5000	3.2	240	
**SC98**	[F]C([H])([H])C([H])([H])OC=3C([H])=C2C1=C(C([H])=C([H])C([H])=C1O[H])C([H])([H])[C@]4([H])C2=C(C=3[H])C([H])([H])C([H])([H])N4C([H])([H])C([H])([H])[H]	5000	0.83	550	
**SC99**	[H]C=1C([H])=C([H])C(OC([H])([H])[H])=C(C=1[H])N2C([H])([H])C([H])([H])N(C([H])([H])C2([H])[H])C([H])([H])C([H])([H])C([H])([H])C([H])([H])N3N=N/C(=C3/[H])C([H])([H])O/C4=C(\[H])C([H])=C([H])C7=C4/C5=C(\[H])C([H])=C([H])C6=C5[C@@]([H])(N(C([H])([H])C6([H])[H])C([H])([H])C([H])([H])C([H])([H])[H])C7([H])[H]	2700	661	2.67	
**SC100**	[H]C=1C([H])=C([H])C(OC([H])([H])[H])=C(C=1[H])N2C([H])([H])C([H])([H])N(C([H])([H])C2([H])[H])C([H])([H])C([H])([H])C([H])([H])C([H])([H])C=3N=NN(C=3[H])C([H])([H])C([H])([H])C([H])([H])O/C4=C(\[H])C([H])=C([H])C7=C4/C5=C(\[H])C([H])=C([H])C6=C5[C@@]([H])(N(C([H])([H])C6([H])[H])C([H])([H])C([H])([H])C([H])([H])[H])C7([H])[H]	1900	206	8.14	[83]
**SC101**	[H]C=1C([H])=C([H])C(OC([H])([H])[H])=C(C=1[H])N2C([H])([H])C([H])([H])N(C([H])([H])C2([H])[H])C([H])([H])C([H])([H])C([H])([H])C([H])([H])O/C3=C(\[H])C([H])=C([H])C6=C3/C4	1174	174	189	

ID	SMILES	Ki [nM][a]			Ref.
		D1R	D2R	D3R	
	=C(\[H])C([H])=C([H])C5=C4[C@@]([H])(N(C([H])([H])C5([H])[H])C([H])([H])C([H])([H])C([H])([H])[H])C6([H])[H]				
**SC102**	[H]C=1C([H])=C([H])C(OC([H])([H])[H])=C(C=1[H])N2C([H])([H])C([H])([H])N(C([H])([H])C2([H])[H])C([H])([H])C([H])([H])C([H])([H])O/C3=C(\[H])C([H])=C([H])C6=C3/C4=C(\[H])C([H])=C([H])C5=C4[C@]([H])(N(C([H])([H])C5([H])[H])C([H])([H])C([H])([H])C([H])([H])[H])C6([H])[H]	1307	141	23.4	
**SC103**	[H]C=1C([H])=C([H])C(=C([H])C=1[H])[C@]2([H])N(C([H])([H])[C@]4([H])[C@@]2([H])[C@@]([H])(N3C([H])([H])C([H])([H])C([H])([H])C3([H])[H])[C@]4([H])C([H])([H])O[H])C([H])([H])C=5C([H])=C([H])C([H])=C([H])C=5[H]	22000	250	280	[84]
**SC104**	[H]C=1C([H])=C([H])C(=C([H])C=1[H])C([H])([H])C([H])([H])N(C([H])([H])C([H])([H])C=2C([H])=C([H])C([H])=C(O[H])C=2[H])C([H])([H])C([H])([H])C([H])([H])[H]	10000	64.4	21.8	
**SC105**	[H]C=1C([H])=C([H])C(=C([H])C=1[H])C([H])([H])C([H])([H])N(C([H])([H])C2([H])C([H])([H])C2([H])[H])C([H])([H])C([H])([H])C3=C([H])C([H])=C([H])C(O[H])=C3[H]	10000	118	168	[85]
**SC106**	[H]C=1C([H])=C([H])C(=C([H])C=1[H])C([H])([H])C([H])([H])N(C([H])([H])C2([H])C([H])([H])C([H])([H])C2([H])[H])C([H])([H])C([H])([H])C3=C([H])C([H])=C([H])C(O[H])=C3[H]	10000	450	282	
**SC107**	O=C1C([H])=NN(C(=O)N1C([H])([H])[H])C([H])([H])C([H])([H])C([H])([H])C([H])([H])N3C([H])([H])C([H])([H])N(C=2C([H])=C([H])C([H])=C([H])C=2OC([H])([H])C([F])([H])[H])C([H])([H])C3([H])[H]	10000	37.1	22	[86]
**SC108**	[H]C=1C([H])=C([H])C(OC([H])([H])C([H])([H])[H])=C(C=1[H])N2C([H])([H])C([H])([H])N(C([H])([H])C2([H])[H])C([H])([H])C=3[S]C([H])=C([H])C=3[H]	10000	162	112	
**SC109**	[H]C=1C([H])=C([H])C(OC([H])([H])C([H])([H])[H])=C(C=1[H])N2C([H])([H])C([H])([H])N(C([H])([H])C2([H])[H])C([H])([H])C=3C([H])=C([H])C([H])=NC=3[H]	10000	255	42	
**SC110**	[Cl]C=1C([H])=C([H])C([H])=C(C=1[Cl])N2C([H])([H])C([H])([H])N(C([H])([H])C2([H])[H])C([H])([H])C=3C([H])=C([H])C([H])=NC=3[H]	10000	327	41	
**SC111**	[H]C=1C([H])=C([H])C2=C(C=1[H])C([H])=C([H])C([H])=C2C([H])([H])N4C([H])([H])C([H])([H])N(C=3C([H])=C([H])C([H])=C([H])C=3OC([H])([H])C([H])([H])[H])C([H])([H])C4([H])[H]	10000	264	59	[87]
**SC112**	[Cl]C=1C([H])=C([H])C([H])=C(C=1[Cl])N2C([H])([H])C([H])([H])N(C([H])([H])C2([H])[H])C([H])([H])C4=C([H])C([H])=C([H])C=3C([H])=C([H])C([H])=C([H])C=34	10000	351	92	
**SC113**	[H]C=2C([H])=C([H])C=1[S]C([H])=C(C=1C=2[H])C([H])([H])N4C([H])([H])C([H])([H])N(C=3C([H])=C([H])C([H])=C([H])C=3OC([H])([H])C([H])([H])[H])C([H])([H])C4([H])[H]	10000	219	45.1	
**SC114**	[Cl]C=1C([H])=C([H])C([H])=C(C=1[Cl])N2C([H])([H])C([H])([H])N(C([H])([H])C2([H])[H])C([H])([H])C3=C([H])[S]C=4C([H])=C([H])C([H])=C([H])C3=4	10000	383	52.5	
**SC115**	[H]C=1C([H])=C([H])C(=C([H])C=1[H])C([H])([H])N3C([H])([H])C([H])([H])N(C=2C([H])=C([H])C([H])=C([H])C=2OC([H])([H])C([H])([H])[H])C([H])([H])C3([H])[H]	10000	217	42.7	

ID	SMILES	Ki [nM][a]			Ref.
		D1R	D2R	D3R	
**SC116**	[Cl]C=1C([H])=C([H])C([H])=C(C=1[Cl])N2C([H])([H])C([H])([H])N(C([H])([H])C2([H])[H])C([H])([H])C=3C([H])=C([H])C([H])=C([H])C=3[H]	10000	242	41	
**SC117**	[H]C=1C([H])=C([H])C2=C(C=1[H])C(=C([H])N2[H])C([H])([H])N4C([H])([H])C([H])([H])N(C=3C([H])=C([H])C([H])=C([H])C=3OC([H])([H])C([H])([H])[H])C([H])([H])C4([H])[H]	10000	473	103	
**SC118**	[Cl]C=1C([H])=C([H])C([H])=C(C=1[Cl])N2C([H])([H])C([H])([H])N(C([H])([H])C2([H])[H])C([H])([H])C3=C([H])N([H])C=4C([H])=C([H])C([H])=C([H])C3=4	10000	502	123	
**SC119**	[H]C=1C([H])=C([H])C(OC([H])([H])[H])=C(C=1[H])N2C([H])([H])C([H])([H])N(C([H])([H])C2([H])[H])C([H])([H])C([H])([H])C([H])([H])C([H])([H])N3N=N/C(=C3/[H])C=4C([H])=C([H])C(=C([H])C=4[H])N([H])[H]	38430	198.8	2.7	[88]
**SC120**	[I]C=1C([H])=C([H])C(=C([H])C=1[H])N2C([H])([H])C([H])([H])N(C([H])([H])C2([H])[H])C([H])([H])C([H])([H])N([H])C([H])([H])[C@]4([H])OC=3C([H])=C(O[H])C([H])=C([H])C=3C([H])([H])C4([H])[H]	3960	80.5	23	[89]
**SC121**	[H]/C1=C(\[H])C(O[H])=C([H])C=2O[C@]([H])(C([H])([H])C([H])([H])C1=2)C([H])([H])N([H])C([H])([H])C([H])([H])C([H])([H])C([H])([H])C([H])([H])C([H])([H])[H]	10900	8.19	547	
**SC122**	[Cl]C=1C([H])=C([H])C(=C([H])C=1[H])N2C([H])([H])C([H])([H])N(C([H])([H])C2([H])[H])C([H])([H])C([H])([H])C([H])([H])C([H])([H])C4=NC=3C([H])=C([H])C([H])=C([H])C=3[S]4	1897	219	31	[56]
**SC123**	O=C(N([H])[C@@]1([H])C([H])([H])C([H])([H])[C@]([H])(C([H])([H])C1([H])[H])C([H])([H])C([H])([H])N3C([H])([H])C([H])([H])N(C=2C([H])=C([H])C([H])=C([Cl])C=2[Cl])C([H])([H])C3([H])[H])N(C([H])([H])[H])C([H])([H])[H]	2100	0.47	0.27	[90]
**SC124**	[Cl]C=1C([H])=C([H])C([H])=C(C=1[Cl])N2C([H])([H])C([H])([H])N(C([H])([H])C2([H])[H])C([H])([H])C([H])([H])C([H])([H])C([H])([H])O/C4=C(\[H])C3=C([H])C([H])=NN3C([H])=C4[H]	2900	34	7	
**SC125**	[Cl]C=1C([H])=C([H])C([H])=C(C=1[Cl])N2C([H])([H])C([H])([H])N(C([H])([H])C2([H])[H])C([H])([H])C([H])([H])C([H])([H])C([H])([H])OC=4/C3=C(\[H])C([H])=NN3C([H])=C([H])C=4[H]	1800	25	10	
**SC126**	[Cl]C=1C([H])=C([H])C([H])=C(C=1[Cl])N2C([H])([H])C([H])([H])N(C([H])([H])C2([H])[H])C([H])([H])C([H])([H])C([H])([H])C([H])([H])OC=4C([H])=C([H])C3=C([H])C([H])=NN3C=4[H]	1200	4.2	2.3	
**SC127**	[Cl]C=1C([H])=C([H])C([H])=C(C=1[Cl])N2C([H])([H])C([H])([H])N(C([H])([H])C2([H])[H])C([H])([H])C([H])([H])C([H])([H])C([H])([H])O/C3=N/N4/C(=C3/[H])C([H])=C([H])C([H])=C4[H]	1100	9.4	3.5	
**SC128**	O=C([H])C=1C([H])=NN2C=1C([H])=C([H])C([H])=C2OC([H])([H])C([H])([H])C([H])([H])C([H])([H])N4C([H])([H])C([H])([H])N(C=3C([H])=C([H])C([H])=C([Cl])C=3[Cl])C([H])([H])C4([H])[H]	1600	51	6.4	
**SC129**	[Cl]/C1=C(\[H])C([H])=C([H])C(=C1[Cl])N2C([H])([H])C([H])([H])N(C([H])([H])C2([H])[H])C([H])([H])C([H])([H])C([H])([H])C([H])([H	4300	1.8	2.3	

ID	SMILES	Ki [nM][a]			Ref.
		D_1R	D_2R	D_3R	
	])C([H])([H])O/C4=C(\[H])C3=C(C([H])=NN3C([H])=C4[H])C([H])=NO[H]				
**SC130**	N#C/C1=N/N4/C(=C1/[H])C([H])=C(OC([H])([H])C([H])([H])C([H])([H])C([H])([H])N3C([H])([H])C([H])([H])N(C=2C([H])=C([H])C([H])=C([Cl])C=2[Cl])C([H])([H])C3([H])[H])C([H])=C4[H]	3500	8.9	1.9	
**SC131**	[F]C=1C([H])=C([H])C(=C([H])C=1[H])C([H])([H])C([H])([H])C([H])([H])C([H])([H])N2[C@@]4([H])C([H])([H])C([H])([H])[C@]2([H])C([H])([H])[C@@](O[H])(C=3C([H])=C([H])C([Cl])=C([H])C=3[H])C4([H])[H]	10000	33	9.7	
**SC132**	[F]C=4C([H])=C([H])C(OC([H])([H])C([H])([H])C([H])([H])N1[C@@]3([H])C([H])([H])C([H])([H])[C@]1([H])C([H])([H])[C@@](O[H])(C=2C([H])=C([H])C([Cl])=C([H])C=2[H])C3([H])[H])=C([H])C=4[H]	10000	22	4.1	[91]
**SC133**	[F]C=1C([H])=C([H])C([H])=C([H])C=1[S]C([H])([H])C([H])([H])C([H])([H])N2[C@@]4([H])C([H])([H])C([H])([H])[C@]2([H])C([H])([H])[C@@](O[H])(C=3C([H])=C([H])C([Cl])=C([H])C=3[H])C4([H])[H]	4093	4.8	1.3	
**SC134**	O=C(C=1C([H])=C([H])C([H])=C([H])C=1OC([H])([H])C([H])([H])C([H])([H])N5C([H])([H])C([H])([H])N(C=2C([H])=C([H])C([H])=C3C=2C([H])=C([H])N3[S](=O)(=O)C=4C([H])=C([H])C([H])=C([H])C=4[H])C([H])([H])C5([H])[H])N([H])[H]	1000	6.3	0.1	
**SC135**	O=C(C=1C([H])=C([H])C(=C([H])C=1[H])C=2C([H])=C([H])C([H])=C([H])C=2[H])N([H])C([H])([H])C([H])([H])C([H])([H])C([H])([H])N([C@]3([H])C([H])([H])C([H])=C(C#C[H])C([H])([H])C3([H])[H])C([H])([H])C([H])([H])C([H])([H])[H]	13000	9.4	1	
**SC136**	O=C(C=1C([H])=C([H])C(=C([H])C=1[H])C=2C([H])=C([H])C([H])=C([H])C=2[H])N([H])C([H])([H])C([H])([H])C([H])([H])C([H])([H])N([C@@]3([H])C([H])([H])C([H])=C(C#C[H])C([H])([H])C3([H])[H])C([H])([H])C([H])([H])C([H])([H])[H]	18000	14	4.3	
**SC137**	O=C(C=2C([H])=C([H])C(OC([H])([H])C([H])([H])C([H])([H])C=1N=NN(C=1[H])C([H])([H])C([H])([H])C([H])([H])C([H])([H])[H])=C(OC([H])([H])[H])C=2[H])N([H])C([H])([H])C([H])([H])C([H])([H])C([H])([H])N([C@]3([H])C([H])([H])C([H])=C(C#C[H])OC3([H])[H])C([H])([H])C([H])([H])C([H])([H])[H]	2600	1.6	0.63	[92]
**SC138**	O=C(C=2C([H])=C([H])C(OC([H])([H])C([H])([H])C([H])([H])C=1N=NN(C=1[H])C([H])([H])C([H])([H])C([H])([H])C([H])([H])[H])=C(OC([H])([H])[H])C=2[H])N([H])C([H])([H])C([H])([H])C([H])([H])C([H])([H])N([C@@]3([H])C([H])([H])C([H])=C(C#C[H])OC3([H])[H])C([H])([H])C([H])([H])C([H])([H])[H]	3900	4.9	2.2	
**SC139**	O=C2C([H])=C([H])C=1/C(=C(/[H])C([H])=C(O[H])C=1N2[H])C([H])([H])C([H])([H])N(C([H])([H])C([H])([H])C([H])([H])[H])C([H])([H])C([H])([H])C([H])([H])C([H])([H])N([H])C(=O)C=4C([H])=C([H])C(OC([H])([H])C([H])([H])C([H])([H])C=3N=NN(C=3[H])C([H])([H])C([H])([H])C([H])([H])C([H])([H])[H])=C(OC([H])([H])[H])C=4[H]	4600	24	1.6	
**SC140**	O=C(C=2C([H])=C([H])C(OC([H])([H])C([H])([H])C([H])([H])C=1N=NN(C=1[H])C([H])([H])C([H])([H])C([H])([H])C([H])([H])[H])=C(OC([H])([H])[H])C=2[H])N([H])C([H])([H])C([H])([H])	23000	18	1.2	

ID	SMILES	Ki [nM][a]			Ref.
		D_1R	D_2R	D_3R	
	C([H])([H])C([H])([H])N([C@@]4([H])C([H])([H])C=3C([H])=C([H])C([H])=C(O[H])C=3C([H])([H])C4([H])[H])C([H])([H])C([H])([H])C([H])([H])[H]				
**SC141**	O=C(C=2C([H])=C([H])C(OC([H])([H])C([H])([H])C([H])([H])C=1N=NN(C=1[H])C([H])([H])C([H])([H])C([H])([H])C([H])([H])[H])=C(OC([H])([H])[H])C=2[H])N([H])C([H])([H])C([H])([H])C([H])([H])C([H])([H])N([C@]4([H])C([H])([H])C=3C([H])=C([H])C([H])=C(O[H])C=3C([H])([H])C4([H])[H])C([H])([H])C([H])([H])C([H])([H])[H]	2900	0.47	0.21	
**SC142**	O=C2N([H])C1=C([H])C([H])=C([H])C(=C1C2([H])[H])C([H])([H])C([H])([H])N(C([H])([H])C([H])([H])C([H])([H])[H])C([H])([H])C([H])([H])C([H])([H])[H]	32500	3.7	2.9	[93]
**SC143**	O=C(/N=N/C=1C([H])=C([F])C([F])=C([F])C=1[H])N([H])C([H])([H])C([H])([H])N(C3([H])C([H])([H])C=2C([H])=C([H])C([H])=C([H])C=2C3([H])[H])C([H])([H])C([H])([H])C([H])([H])[H]	2600	110	87	[94]
**SC144**	O=C(/N=N/C=1C([H])=C([H])C([F])=C([H])C=1[H])N([H])C([H])([H])C([H])([H])C([H])([H])C([H])([H])N3C([H])([H])C([H])([H])N(C=2C([H])=C([H])C([H])=C([H])C=2OC([H])([H])[H])C([H])([H])C3([H])[H]	1300	38	3	
**SC145**	O=C(C([H])=C([H])C=1C([H])=C([H])C([F])=C([H])C=1[H])N([H])C([H])([H])C([H])([H])C([H])([H])C([H])([H])N(C3([H])C([H])([H])C=2C([H])=C([H])C([H])=C([H])C=2C3([H])[H])C([H])([H])C([H])([H])C([H])([H])[H]	1300	19	0.31	
**SC146**	O=C(/N=N/C=1C([H])=C([H])C([F])=C([H])C=1[H])N([H])C([H])([H])C([H])([H])C([H])([H])C([H])([H])N(C3([H])C([H])([H])C=2C([H])=C([H])C([H])=C([H])C=2C3([H])[H])C([H])([H])C([H])([H])C([H])([H])[H]	3300	23	0.85	
**SC147**	O=C(C([H])=C([H])C=1C([H])=C([H])C([F])=C([H])C=1[H])N([H])C([H])([H])C([H])([H])N(C3([H])C([H])([H])C=2C([H])=C([H])C([H])=C([H])C=2C3([H])[H])C([H])([H])C([H])([H])C([H])([H])[H]	2200	43	14	
**SC148**	[H]C=1C([H])=C([H])C(OC([H])([H])[H])=C(C=1[H])N2C([H])([H])C([H])([H])N(C([H])([H])C2([H])[H])C([H])([H])C([H])([H])C([H])([H])C=3N=NN(C=3[H])C5=C([H])C4=C([H])C([H])=NN4C([H])=C5[H]	1700	140	77	[95]
**SC149**	O=C([H])C=1C([H])=NN2C=1C([H])=C(C([H])=C2[H])N3N=N/C(=C3/[H])C([H])([H])C([H])([H])C([H])([H])C([H])([H])N5C([H])([H])C([H])([H])N(C=4C([H])=C([H])C([H])=C([Cl])C=4[Cl])C([H])([H])C5([H])[H]	1500	110	4.4	
**SC150**	O=C1N([H])C=2C(OC1([H])[H])=C(C([H])=C([H])C=2O[H])[C@]([H])(O[H])C([H])([H])N([H])C([H])([H])C([H])([H])C=4C([H])=C([H])C(OC([H])([H])C([H])([H])C([H])([H])C=3C([H])=C([H])C([H])=C([H])C=3[H])=C(OC([H])([H])[H])C=4[H]	2800	310	400	[96]
**SC151**	O=C1N([H])C=2C(OC1([H])[H])=C(C([H])=C([H])C=2O[H])[C@@]([H])(O[H])C([H])([H])N([H])C([H])([H])C([H])([H])C=4C([H])=C([H])C(OC([H])([H])C([H])([H])C([H])([H])C=3C([H])=C([H])C([H])=C([H])C=3[H])=C(OC([H])([H])[H])C=4[H]	4500	130	690	
**SC152**	O=C2C([H])=C([H])C=1/C(=C(/[H])C([H])=C(O[H])C=1N2[H])C([H])([H])C([H])([H])N([H])C([H])([H])C([H])([H])C=4C([H])	2800	64	18	

ID	SMILES	Ki [nM][a]			Ref.
		D1R	D2R	D3R	
	=C([H])C(OC([H])([H])C([H])([H])C([H])([H])C=3C([H])=C([H])C([H])=C([H])C=3[H])=C(OC([H])([H])[H])C=4[H]				
**SC153**	O=C(N([H])C2=C([H])C1=C([H])C([H])=NN1C([H])=C2[H])C([H])([H])C([H])([H])C([H])([H])N4C([H])([H])C([H])([H])N(C=3C([H])=C([H])C([H])=C([H])C=3OC([H])([H])[H])C([H])([H])C4([H])[H]	1700	46	97	[97]
**SC154**	O=C([H])C=1C([H])=NN4C=1C([H])=C([H])C(OC([H])([H])C([H])([H])C([H])([H])C([H])([H])N3C([H])([H])C([H])([H])N(C=2C([H])=C([H])C([H])=C([H])C=2OC([H])([H])[H])C([H])([H])C3([H])[H])=C4[H]	1400	7.8	17	
**SC155**	O=C(N([H])C2=C([H])C1=C(C([H])=NN1C([H])=C2[H])C([H])=NO[H])C([H])([H])C([H])([H])C([H])([H])N4C([H])([H])C([H])([H])N(C=3C([H])=C([H])C([H])=C([Cl])C=3[Cl])C([H])([H])C4([H])[H]	2900	0.57	2.2	
**SC156**	[H]C=1C([H])=C([H])C(OC([H])([H])[H])=C(C=1[H])N2C([H])([H])C([H])([H])N(C([H])([H])C2([H])[H])C([H])([H])C([H])([H])C([H])([H])C([H])([H])OC=4C([H])=C([H])C3=C(C([H])=NN3C=4[H])C([H])=NO[H]	1000	4.2	2.3	
**SC157**	O=C([H])C=1C([H])=NN4C=1C([H])=C([H])C(OC([H])([H])C([H])([H])C([H])([H])C([H])([H])N([C@]3([H])C([H])([H])C=2C([H])=C([H])C([H])=C(O[H])C=2C([H])([H])C3([H])[H])C([H])([H])C([H])([H])C([H])([H])[H])=C4[H]	1400	2.4	1.6	
**SC158**	[H]C=1C([H])=C([H])C(OC([H])([H])[H])=C(C=1[H])N2C([H])([H])C([H])([H])N(C([H])([H])C2([H])[H])C([H])([H])C([H])([H])C([H])([H])C([H])([H])O/C4=C(\[H])C3=C([H])C([H])=NN3C([H])=C4[H]	2800	18	20	
**SC159**	O=C([H])C=1C([H])=NN2C=1C([H])=C(C([H])=C2[H])N([H])C(=O)C([H])([H])C([H])([H])C([H])([H])N4C([H])([H])C([H])([H])N(C=3C([H])=C([H])C([H])=C([H])C=3OC([H])([H])[H])C([H])([H])C4([H])[H]	1200	1.4	70	
**SC160**	O=C([H])C=1C([H])=NN4C=1C([H])=C(OC([H])([H])C([H])([H])C([H])([H])C([H])([H])N([C@]3([H])C([H])([H])C=2C([H])=C([H])C([H])=C(O[H])C=2C([H])([H])C3([H])[H])C([H])([H])C([H])([H])C([H])([H])[H])C([H])=C4[H]	1300	2.3	1.1	
**SC161**	[H]/C1=C(\[H])C4=C(C(O[H])=C1[H])C([H])([H])C([H])([H])[C@@]([H])(N(C([H])([H])C([H])([H])C([H])([H])[H])C([H])([H])C([H])([H])C([H])([H])C([H])([H])O/C3=C(\[H])C2=C([H])C([H])=NN2C([H])=C3[H])C4([H])[H]	3700	96	13	
**SC162**	[H]/C1=C(\[H])C4=C(C(O[H])=C1[H])C([H])([H])C([H])([H])[C@]([H])(N(C([H])([H])C([H])([H])C([H])([H])[H])C([H])([H])C([H])([H])C([H])([H])C([H])([H])OC=3C([H])=C([H])C2=C([H])C([H])=NN2C=3[H])C4([H])[H]	2100	0.63	0.71	
**SC163**	[H]C=1C([H])=C([H])C(OC([H])([H])[H])=C(C=1[H])N2C([H])([H])C([H])([H])N(C([H])([H])C2([H])[H])C([H])([H])C([H])([H])C([H])([H])C([H])([H])OC=4C([H])=C([H])C3=C([H])C([H])=NN3C=4[H]	1100	1.8	5	
**SC164**	[H]/C1=C(\[H])C4=C(C(O[H])=C1[H])C([H])([H])C([H])([H])[C@]([H])(N(C([H])([H])C([H])([H])C([H])([H])[H])C([H])([H])	1800	2.2	0.59	

ID	SMILES	Ki [nM][a]			Ref.
		D1R	D2R	D3R	
	C([H])([H])C([H])([H])C([H])([H])O/C3=C(\[H])C2=C(C([H])=NN2C([H])=C3[H])C([H])=NO[H])C4([H])[H]				
**SC165**	[H]/C1=C(\[H])C4=C(C(O[H])=C1[H])C([H])([H])C([H])([H])[C@@]([H])(N(C([H])([H])C([H])([H])C([H])([H])[H])C([H])([H])C([H])([H])C([H])([H])C([H])([H])OC=3C([H])=C([H])C2=C([H])C([H])=NN2C=3[H])C4([H])[H]	8600	120	11	
**SC166**	[H]/C1=C(\[H])C2=C(/N=C1/[H])C(O[H])=C([H])C([H])=C2N3C([H])([H])C([H])([H])N(C([H])([H])C3([H])[H])C([H])([H])C([H])([H])C([H])([H])C([H])([H])O/C5=C(\[H])C4=C([H])C([H])=NN4C([H])=C5[H]	1100	33	50	
**SC167**	O=C5N([H])C=4C([H])=C(OC([H])([H])C([H])([H])C([H])([H])C([H])([H])N3C([H])([H])C([H])([H])N(/C2=C(\[H])C([H])=C(O[H])C=1/N=C(/[H])C([H])=C([H])C=12)C([H])([H])C3([H])[H])C([H])=C([H])C=4C([H])([H])C5([H])[H]	1500	3.4	27	
**SC168**	O=C5N([H])C=4C([H])=C(OC([H])([H])C([H])([H])C([H])([H])C([H])([H])N3C([H])([H])C([H])([H])C([H])([H])N(C=1C([H])=C([H])C(O[H])=C2C=1OC([H])([H])C(=O)N2[H])C([H])([H])C3([H])[H])C([H])=C([H])C=4C([H])([H])C5([H])[H]	2700	1.6	2	
**SC169**	O=C2C([H])=C([H])C=1/C(=C(/[H])C([H])=C(O[H])C=1N2[H])N3C([H])([H])C([H])([H])C([H])([H])N(C([H])([H])C3([H])[H])C([H])([H])C([H])([H])C([H])([H])C([H])([H])N([H])C(=O)C=5C([H])=C([H])C(OC([H])([H])C([H])([H])C([H])([H])C=4N=NN(C=4[H])C([H])([H])C([H])([H])C([H])([H])C([H])([H])[H])=C(OC([H])([H])[H])C=5[H]	4000	200	3.2	
**SC170**	O=C2C([H])=C([H])C=1/C(=C(/[H])C([H])=C(O[H])C=1N2[H])N3C([H])([H])C([H])([H])C([H])([H])N(C([H])([H])C3([H])[H])C([H])([H])C([H])([H])C([H])([H])N([H])C(=O)C=5C([H])=C([H])C(OC([H])([H])C([H])([H])C([H])([H])C=4N=NN(C=4[H])C([H])([H])C([H])([H])C([H])([H])C([H])([H])[H])=C(OC([H])([H])[H])C=5[H]	17000	350	1.6	[98]
**SC171**	O=C5N([H])C=4C([H])=C(OC([H])([H])C([H])([H])C([H])([H])C([H])([H])N3C([H])([H])C([H])([H])N(C=1C([H])=C([H])C(O[H])=C2C=1OC([H])([H])C(=O)N2[H])C([H])([H])C3([H])[H])C([H])=C([H])C=4C([H])([H])C5([H])[H]	12000	0.87	1	
**SC172**	O=C5OC1=C(/C(=C(/[H])C([H])=C1O[H])N2C([H])([H])C([H])([H])N(C([H])([H])C2([H])[H])C([H])([H])C([H])([H])C([H])([H])C([H])([H])O/C4=C(\[H])C3=C([H])C([H])=NN3C([H])=C4[H])C([H])=C5[H]	20000	40	8.5	
**SC173**	O=C2C([H])=C([H])C=1/C(=C(/[H])C([H])=C(O[H])C=1N2[H])N3C([H])([H])C([H])([H])N(C([H])([H])C3([H])[H])C([H])([H])C([H])([H])C([H])([H])C([H])([H])OC=4C([H])=C([H])C5=C(C=4[H])N([H])C(=O)C([H])([H])C5([H])[H]	5400	0.89	12	
**SC174**	[H]/C2=C(\[H])C(O[H])=C1OC([H])([H])C([H])([H])O/C1=C2\N3C([H])([H])C([H])([H])N(C([H])([H])C3([H])[H])C([H])([H])C([H])([H])C([H])([H])C([H])([H])O/C5=C(\[H])C4=C([H])C([H])=NN4C([H])=C5[H]	5300	110	40	
**SC175**	O=C5OC1=C(/C(=C(/[H])C([H])=C1O[H])N2C([H])([H])C([H])([H])C([H])([H])N(C([H])([H])C2([H])[H])C([H])([H])C([H])([H	20000	100	240	

ID	SMILES	Ki [nM][a]			Ref.
		D1R	D2R	D3R	
	])C([H])([H])C([H])([H])OC=3C([H])=C([H])C4=C(C=3[H])N([H])C(=O)C([H])([H])C4([H])[H])C([H])=C5[H]				
**SC176**	O=C2C([H])=C([H])C=1/C(=C(/[H])C([H])=C(O[H])C=1N2[H])N3C([H])([H])C([H])([H])C([H])([H])N(C([H])([H])C3([H])[H])C([H])([H])C([H])([H])C([H])([H])C([H])([H])O/C5=C(\[H])C4=C([H])C([H])=NN4C([H])=C5[H]	13000	78	3.4	
**SC177**	O=C5N([H])C=4C([H])=C(OC([H])([H])C([H])([H])C([H])([H])C([H])([H])N3C([H])([H])C([H])([H])N(C=1C([H])=C([H])C(O[H])=C2OC([H])([H])C([H])([H])OC=12)C([H])([H])C3([H])[H])C([H])=C([H])C=4C([H])([H])C5([H])[H]	4500	2.9	38	
**SC178**	O=C2C([H])=C([H])C=1/C(=C(/[H])C([H])=C(O[H])C=1N2[H])N3C([H])([H])C([H])([H])N(C([H])([H])C3([H])[H])C([H])([H])C([H])([H])C([H])([H])C([H])([H])O/C5=C(\[H])C4=C([H])C([H])=NN4C([H])=C5[H]	4700	8.3	3.8	
**SC179**	O=C5OC1=C(/C(=C(/[H])C([H])=C1O[H])N2C([H])([H])C([H])([H])N(C([H])([H])C2([H])[H])C([H])([H])C([H])([H])C([H])([H])C([H])([H])OC=3C([H])=C([H])C4=C(C=3[H])N([H])C(=O)C([H])([H])C4([H])[H])C([H])=C5[H]	20000	4.8	7.7	
**SC180**	O=C1N([H])C2=C(OC1([H])[H])C(=C([H])C([H])=C2O[H])N3C([H])([H])C([H])([H])N(C([H])([H])C3([H])[H])C([H])([H])C([H])([H])C([H])([H])C([H])([H])O/C5=C(\[H])C4=C([H])C([H])=NN4C([H])=C5[H]	2300	2	0.71	
**SC181**	O=C2C([H])=C([H])C=1/C(=C(/[H])C([H])=C(O[H])C=1N2[H])N3C([H])([H])C([H])([H])C([H])([H])N(C([H])([H])C3([H])[H])C([H])([H])C([H])([H])C([H])([H])C([H])([H])OC=4C([H])=C([H])C5=C(C=4[H])N([H])C(=O)C([H])([H])C5([H])[H]	11000	1.6	1.9	
**SC182**	[I]C=1C([H])=C([H])C(=C([H])C=1[H])C([H])([H])N3C([H])([H])C([H])([H])C([H])(N(C=2/N=C(/[H])C([H])=C([H])C=2OC([H])(C([H])([H])[H])C([H])([H])[H])C([H])([H])[H])C([H])([H])C3([H])[H]	2830	568	145	[72]
**SC183**	O=C1N([H])C2=C(OC1([H])[H])C(=C([H])C([H])=C2O[H])N3C([H])([H])C([H])([H])N(C([H])([H])C3([H])[H])C([H])([H])C([H])([H])C([H])([H])C([H])([H])O/C5=C(\[H])C4=C(C([H])=NN4C([H])=C5[H])C([H])=NO[H]	4800	0.91	0.35	
**SC184**	O=C1N([H])C=2C(OC1([H])[H])=C(C([H])=C([H])C=2O[H])N3C([H])([H])C([H])([H])C([H])([H])N(C([H])([H])C3([H])[H])C([H])([H])C([H])([H])C([H])([H])[H]	8800	360	3.7	
**SC185**	O=C2C([H])=C([H])C=1/C(=C(/[H])C([H])=C(O[H])C=1N2[H])N3C([H])([H])C([H])([H])N(C([H])([H])C3([H])[H])C([H])([H])C([H])([H])C([H])([H])C([H])([H])O/C5=C(\[H])C4=C(C([H])=NN4C([H])=C5[H])C([H])=NO[H]	20000	3.4	2.7	[99]
**SC186**	O=C1N([H])C=2C(OC1([H])[H])=C(C([H])=C([H])C=2O[H])N3C([H])([H])C([H])([H])N(C([H])([H])C3([H])[H])C([H])([H])C([H])([H])C([H])([H])C([H])([H])O/C5=C(\[H])C4=C(C([H])=NN4C([H])=C5[H])C(=O)[H]	12000	1.1	1.2	
**SC187**	[H]/C2=C(\[H])C(O[H])=C1OC([H])([H])C([H])([H])O/C1=C2\N3C([H])([H])C([H])([H])N(C([H])([H])C3([H])[H])C([H])([H])	20000	1.7	35	

ID	SMILES	Ki [nM] [a]			Ref.
		D_1R	D_2R	D_3R	
	C([H])([H])C([H])([H])C([H])([H])O/C5=C(\[H])C4=C(C([H])=NN4C([H])=C5[H])C([H])=NO[H]				
**SC188**	O=C1N([H])C=2C(OC1([H])[H])=C(C([H])=C([H])C=2O[H])N3C([H])([H])C([H])([H])N(C([H])([H])C3([H])[H])C([H])([H])C([H])([H])C([H])([H])[H]	20000	120	5.9	
**SC189**	O=C2C([H])=C([H])C=1/C(=C(/[H])C([H])=C(O[H])C=1N2[H])N3C([H])([H])C([H])([H])N(C([H])([H])C3([H])[H])C([H])([H])[H]	20000	780	380	
**SC190**	O=C([H])C=1C([H])=NN5C=1C([H])=C(OC([H])([H])C([H])([H])C([H])([H])C([H])([H])N4C([H])([H])C([H])([H])N(C=2C([H])=C([H])C(O[H])=C3OC([H])([H])C([H])([H])OC=23)C([H])([H])C4([H])[H])C([H])=C5[H]	11000	5.7	24	
**SC191**	O=C1N([H])C=2C(OC1([H])[H])=C(C([H])=C([H])C=2O[H])N3C([H])([H])C([H])([H])N(C([H])([H])C3([H])[H])C([H])([H])[H]	20000	630	48	
**SC192**	O=C2C([H])=C([H])C=1/C(=C(/[H])C([H])=C(O[H])C=1N2[H])N3C([H])([H])C([H])([H])N(C([H])([H])C3([H])[H])C([H])([H])C([H])([H])C([H])([H])C([H])([H])O/C5=C(\[H])C4=C(C([H])=NN4C([H])=C5[H])C(=O)[H]	15000	1	2.3	
**SC193**	O=C(/C1=N/N2/C(=C1/[H])C([H])=C([H])C([H])=C2[H])N([H])C([H])([H])C([H])([H])C([H])([H])C([H])([H])N4C([H])([H])C([H])([H])N(C=3C([H])=C([H])C([H])=C([H])C=3OC([H])([H])[H])C([H])([H])C4([H])[H]	1600	270	3.5	[100]
**SC194**	O=C(/C1=N/N2/C(=C1/[H])C([H])=C([H])C([H])=C2[H])N([H])C([H])([H])C([H])([H])C([H])([H])C([H])([H])N4C([H])([H])C([H])([H])N(/C3=N/C(=N/C(=C3/[H])C([F])([F])[F])C(C([H])([H])[H])(C([H])([H])[H])C([H])([H])[H])C([H])([H])C4([H])[H]	7500	430	1.2	
**SC195**	O=C(C=1C([H])=C([H])C(=C([H])C=1[H])N(C([H])([H])[H])C([H])([H])[H])N([H])C([H])([H])C([H])([H])C([H])([H])C([H])([H])N3C([H])([H])C([H])([H])[C@]([H])(C=2C([H])=C([H])C([H])=C(O[H])C=2[H])C3([H])[H]	10000	345	27	[101]
**SC196**	[F]C([F])([F])C=1C([H])=C(C([H])=C([H])C=1[Cl])C2(O[H])C([H])([H])C([H])([H])N(C([H])([H])C2([H])[H])C([H])([H])C([H])([H])C([H])([H])C([H])([H])N(C=3C([H])=C([H])C(OC([H])([H])[H])=C([H])C=3[H])C=4C([H])=C([H])C(OC([H])([H])[H])=C([H])C=4[H]	10000	417	437	[102]
**SC197**	N#CC=1C([H])=C([H])C([H])=C(C=1[H])N2C([H])([H])C([H])([H])N(C([H])([H])C2([H])[H])C([H])([H])C([H])([H])C([H])([H])C([H])([H])N([H])C(=O)C=3C([H])=C([H])C(=C([H])C=3[H])C=4C([H])=C([H])[S]C=4[H]	10000	743	0.5	[103]
**SC198**	[F]C=1C([H])=C([H])C(OC([H])([H])[H])=C(C=1[H])[C@@]2([H])C([H])([H])[C@]2([H])C([H])([H])N(C([H])([H])C([H])([H])C([H])([H])[S]/C4=N/N=C(/C=3C([H])=C([H])C([H])=C([H])C=3[H])N4C([H])([H])[H])C([H])(C([H])([H])[H])C([H])([H])[H]	5000	512.9	104.7	[104]
**SC199**	O=C(C=2C([H])=C([H])C=1C([H])=C([H])C([H])=C([H])C=1C=2[H])N([H])C([H])([H])C([H])([H])C([H])([H])C([H])([H])N(C([H])([H])[C@]3([H])C([H])([H])[C@@]3([H])C=4C([H])=C([Cl])C([H])=C([H])C=4OC([H])([H])[H])C([H])([H])C([H])([H])[H]	1981	408	23.4	

ID	SMILES	Ki [nM][a]			Ref.
		D_1R	D_2R	D_3R	
**SC200**	[Cl]C=1C([H])=C([H])C(OC([H])([H])[H])=C(C=1[H])[C@]2([H])C([H])([H])[C@]2([H])C([H])([H])N(C([H])([H])C([H])([H])[H])C([H])([H])C([H])([H])C([H])([H])[S]/C4=N/N=C(/C=3C([H])=C([H])C([H])=C([H])C=3[H])N4C([H])([H])[H]	1509	238	31.6	
**SC201**	[Cl]C=1C([H])=C([H])C(OC([H])([H])[H])=C(C=1[H])[C@]2([H])C([H])([H])[C@@]2([H])C([H])([H])N(C([H])([H])C([H])([H])C([H])([H])[H])C([H])([H])C([H])([H])C([H])([H])[S]/C4=N/N=C(/C=3C([H])=C([H])C([H])=C([H])C=3[H])N4C([H])([H])[H]	1918	233	36.7	
**SC202**	[F]C=1C([H])=C([H])C(OC([H])([H])[H])=C(C=1[H])[C@@]2([H])C([H])([H])[C@]2([H])C([H])([H])N(C([H])([H])C([H])([H])[H])C([H])([H])C([H])([H])C([H])([H])[S]/C4=N/N=C(/C=3C([H])=C([H])C([H])=C([H])C=3[H])N4C([H])([H])[H]	1724.6	275.4	44	
**SC203**	[H]C2([H])C=1N=C([S]C=1C([H])([H])[C@@]([H])(N([H])C([H])([H])C([H])([H])C([H])([H])[H])C2([H])[H])N([H])[H]	10000	743.5	0.9	[105]
**SC204**	O=C(/C2=C(\[H])C=1C([H])=C([H])C([H])=C([H])C=1N2[H])N([H])C([H])([H])C([H])([H])[C@@]3([H])C([H])([H])[C@]3([H])C([H])([H])N4C([H])([H])[C@@]([H])(OC([H])([H])[C@]4([H])C([H])([H])[H])C=5C([H])=C([H])C(=NC=5[H])N([H])[H]	5630	87.8	1.85	[106]
**SC205**	O=C(/C2=C(\[H])C=1C([H])=C([H])C([H])=C([H])C=1N2[H])N([H])[C@@]3([H])C([H])([H])C([H])([H])[C@]([H])(C([H])([H])C3([H])[H])C([H])([H])C([H])([H])N4C([H])([H])[C@@]([H])(OC([H])([H])[C@]4([H])C([H])([H])[H])C=5C([H])=C([H])C(=NC=5[H])N([H])[H]	100000	104	21.6	
**SC206**	[H]/C1=C(\[H])C(O[H])=C([H])C2=C1OC([H])([H])[C@]3([H])N(C([H])([H])C([H])([H])O[C@]23[H])C([H])([H])C([H])([H])C([H])([H])[H]	100000	28	1.67	

[a]All KI values retrieved from ChEMBL were determined in vitro utilizing radio-ligand binding assays.

2.4 Dataset of D_3R-selective compounds (ChEMBL)

Table S4. D_3R-selective compounds extracted from ChEMBL with in vitro determined biological activities for all investigated DR subtypes.

ID	SMILES	Ki [nM][a]			Ref.
		D_1R	D_2R	D_3R	
**SC207**	O=C(C=1C([H])=C([Cl])C(=C([H])C=1OC([H])([H])[H])N([H])[H])N([H])C([H])([H])[C@]2([H])C([H])([H])C([H])([H])N(C2([H])[H])C([H])([H])C=3C([H])=C([H])C([H])=C([H])C=3[H]	11000	1400	570	[62]
**SC208**	[H]C=1C([H])=C([H])C(=C([H])C=1[H])C2=N/C(=C(/[H])[S]2)C([H])([H])N([H])C([H])([H])[C@]3([H])C([H])([H])C([H])([H])N(C3([H])[H])C([H])([H])C=4C([H])=C([H])C([H])=C([H])C=4[H]	8850	7900	660	[63]
**SC209**	[Br]C=1C([H])=C([H])C(OC([H])([H])[H])=C(C=1[H])C2=N/C(=C(/[H])O2)C([H])([H])N([H])C([H])([H])C([H])([H])[C@]3([H])C([H])([H])C([H])([H])N(C3([H])[H])C([H])([H])C=4C([H])=C([H])C([H])=C([H])C=4[H]	8950	1070	120	
**SC210**	[H]C=1C([H])=C([H])C(=C([H])C=1[H])N2C([H])([H])C([H])([H])N(C([H])([H])C2([H])[H])C([H])([H])C3=C([H])C([H])=C([H])N3[H]	5400	3000	570	[107]

ID	SMILES	Ki [nM][a]			Ref.
		D_1R	D_2R	D_3R	
**SC211**	O=C(C=2[S]C=1C([H])=C([H])C([H])=C([H])C=1C=2[H])N([H])C([H])([H])C([H])([H])C([H])([H])C([H])([H])N4C([H])([H])C([H])([H])N(C=3C([H])=C([H])C([H])=C([Cl])C=3[Cl])C([H])([H])C4([H])[H]	8800	3600	0.5	[108]
**SC212**	O=C(C=2C([H])=C([Br])C=1C([H])=C([H])C([H])=C([H])C=1C=2OC([H])([H])[H])N([H])C([H])([H])C([H])([H])[C@]3([H])C([H])([H])C([H])([H])N(C3([H])[H])C([H])([H])C=4C([H])=C([H])C([H])=C([H])C=4[H]	1700	2000	580	[65]
**SC213**	O=C(C=2OC=1C([H])=C([H])C([I])=C([H])C=1C=2[H])N([H])C([H])([H])C([H])([H])C([H])([H])C([H])([H])N4C([H])([H])C([H])([H])N(C=3C([H])=C([H])C([H])=C([Cl])C=3[Cl])C([H])([H])C4([H])[H]	3300	3200	5.7	[109]
**SC214**	O=C(C=2C([H])=C([H])C(C#CC=1C([H])=C([H])C([H])=C([H])C=1[H])=C([H])C=2[H])N([H])C([H])([H])C([H])([H])N4C([H])([H])C([H])([H])N(C=3C([H])=C([H])C([H])=C([Cl])C=3[Cl])C([H])([H])C4([H])[H]	5500	4700	29	[110]
**SC215**	O=C(C=2C([H])=C([H])C([H])=C(C#CC=1C([H])=C([H])C([H])=C([H])C=1[H])C=2[H])N([H])C([H])([H])C([H])([H])N4C([H])([H])C([H])([H])N(C=3C([H])=C([H])C([H])=C([Cl])C=3[Cl])C([H])([H])C4([H])[H]	3100	1200	68	
**SC216**	O=C(C=2C([H])=C([H])C(C#CC=1C([H])=C([H])C([H])=C([H])C=1[H])=C([H])C=2[H])N([H])C([H])([H])C([H])([H])C([H])([H])C([H])([H])N4C([H])([H])C([H])([H])N(C=3C([H])=C([H])C([H])=C([H])C=3OC([H])([H])[H])C([H])([H])C4([H])[H]	16000	1400	10	
**SC217**	O=C(C=2C([H])=C([H])C([H])=C(C#CC=1C([H])=C([H])C([H])=C([H])C=1[H])C=2[H])N([H])C([H])([H])C([H])([H])C([H])([H])C([H])([H])N4C([H])([H])C([H])([H])N(C=3C([H])=C([H])C([H])=C([H])C=3OC([H])([H])[H])C([H])([H])C4([H])[H]	5900	1200	22	
**SC218**	[Cl]C=1C([H])=C([H])C(=C([H])C=1[Cl])N2C([H])([H])C([H])([H])N(C([H])([H])C2([H])[H])C([H])([H])C=4N=C3C([H])=C([H])C(=NN3C=4[H])N5C([H])([H])C([H])([H])N(C([H])([H])C5([H])[H])C([H])([H])[H]	13000	8000	880	[66]
**SC219**	O=C(/C2=C(\[H])C=1C([H])=C([H])C([H])=C([H])C=1C([H])([H])N2[H])N([H])C([H])([H])C([H])([H])C([H])([H])C([H])([H])N5C([H])([H])C4=C([H])C=3C([H])=C([H])C([H])=C(OC([H])([H])[H])C=3N4C([H])([H])C5([H])[H]	10000	1012	33.5	[111]
**SC220**	O=C(/C2=N/C([H])=C1C([H])=C([H])C([H])=C([H])C1=C2[H])N([H])C([H])([H])C([H])([H])C([H])([H])C([H])([H])N4C([H])([H])C([H])([H])N(C=3C([H])=C([H])C([H])=C([Cl])C=3[H])C([H])([H])C4([H])[H]	5500	1039	0.27	
**SC221**	O=C3/C2=C(\[H])C=1C([H])=C([H])C([H])=C([H])C=1N2C([H])([H])C([H])([H])N3C([H])([H])C([H])([H])C([H])([H])C([H])([H])N6C([H])([H])C5=C([H])C=4C([H])=C([H])C([H])=C(OC([H])([H])[H])C=4N5C([H])([H])C6([H])[H]	10000	3530	8.6	
**SC222**	O=C(/C2=C(\[H])C=1C([H])=C([H])C([H])=C([H])C=1C([H])([H])N2[H])N([H])C([H])([H])C([H])([H])C([H])([H])C([H])([H])N5C([H])([H])C4=N/C3=C(\[H])C([H])=C([H])C(OC([H])([H])[H])=C3[C@]4([H])C([H])([H])C5([H])[H]	10000	6475	6	

ID	SMILES	Ki [nM][a]			Ref.
		D_1R	D_2R	D_3R	
**SC223**	[H]/C1=C(/N=NN1C([H])([H])C([H])([H])C([H])([H])C([H])([H])N([C@@]2([H])C([H])([H])C([H])=C(C#CC([H])([H])[H])C([H])([H])C2([H])[H])C([H])([H])C([H])([H])C([H])([H])[H])C([H])([H])[H]	100000	4100	280	[112]
**SC224**	O=C(C=1C([H])=C([H])C(=C([H])C=1[H])C=2C([H])=C([H])C([H])=C([H])C=2[H])N([H])C([H])([H])C([H])([H])C([H])([H])C([H])([H])N(C([H])([H])C([H])([H])C([H])([H])[H])C([H])([H])C([H])([H])C([H])([H])[H]	6800	9700	950	
**SC225**	O=C(C=1C([H])=C([H])C(=C([H])C=1[H])C=2C([H])=C([H])C([H])=C([H])C=2[H])N([H])C([H])([H])C([H])([H])C([H])([H])C([H])([H])N([C@]4([H])C([H])([H])C([H])=C(C#CC=3C([H])=C([H])C([H])=C([H])C=3[H])C([H])([H])C4([H])[H])C([H])([H])C([H])([H])C([H])([H])[H]	13000	5500	300	
**SC226**	O=C(C=1C([H])=C([H])C(=C([H])C=1[H])C=2C([H])=C([H])C([H])=C([H])C=2[H])N([H])C([H])([H])C([H])([H])C([H])([H])C([H])([H])N(C3([H])C([H])([H])C([H])([H])C([H])([H])C([H])([H])C3([H])[H])C([H])([H])C([H])([H])C([H])([H])[H]	4600	4700	140	
**SC227**	O=C(C=2C([H])=C([H])C(OC([H])([H])C([H])([H])C([H])([H])N1C([H])([H])C([H])([H])C([H])([H])C([H])([H])C1([H])[H])=C([H])C=2[H])N(C([H])([H])[H])C([H])([H])C([H])([H])C([H])=C5C=3C([H])=C([H])C([H])=C([H])C=3C([H])([H])C([H])([H])C=4C([H])=C([H])C([H])=C([H])C=45	1000	1000	243	[113]
**SC228**	[H]C=1C([H])=C([H])N=C(C=1[H])N2C([H])([H])C([H])([H])N(C([H])([H])C2([H])[H])C([H])([H])C3=C(N=C4C([H])=C(N=C(N34)C([H])([H])[H])C([H])([H])[H])C([H])([H])[H]	22000	17000	640	[53]
**SC229**	O=C(/C2=C(\[H])C=1C([H])=C([I])C([H])=C([H])C=1N2[H])N([H])C([H])([H])C([H])([H])[C@@]([H])(O[H])C([H])([H])N4C([H])([H])C([H])([H])N(C=3C([H])=C([H])C([H])=C([Cl])C=3[Cl])C([H])([H])C4([H])[H]	10000	1060	5.21	[79]
**SC230**	O=C([H])C=1C([H])=NN4C=1C([H])([H])[C@]([H])(N(C([H])([H])C([H])([H])C([H])([H])[H])C([H])([H])C([H])([H])C([H])([H])C([H])([H])N([H])C(=O)C=2C([H])=C([H])C(=C([H])C=2[H])C=3C([H])=C([H])C([H])=C([H])C=3[H])C([H])([H])C4([H])[H]	23000	8400	39	[114]
**SC231**	[Cl]C=1C([H])=C([H])C([H])=C([H])C=1N2C([H])([H])C([H])([H])N(C([H])([H])C2([H])[H])C([H])([H])C=3[S]C([H])=C([H])C=3[H]	10000	2179	427	[87]
**SC232**	[Cl]C=1C([H])=C([H])C([H])=C(C=1[Cl])N2C([H])([H])C([H])([H])N(C([H])([H])C2([H])[H])C([H])([H])C=3[S]C([H])=C([H])C=3[H]	10000	1545	44.5	
**SC233**	[Cl]C=1C([H])=C([H])C(=C([H])C=1[Cl])N2C([H])([H])C([H])([H])N(C([H])([H])C2([H])[H])C([H])([H])C=3[S]C([H])=C([H])C=3[H]	2541	1593	62	
**SC234**	[H]C=1C([H])=C([S]C=1[H])C([H])([H])N3C([H])([H])C([H])([H])N(C=2C([H])=C([H])C(O[H])=C([H])C=2[H])C([H])([H])C3([H])[H]	10000	2167	221	
**SC235**	[I]C=1C([H])=C([H])C(=C([H])C=1[H])N2C([H])([H])C([H])([H])N(C([H])([H])C2([H])[H])C([H])([H])[C@]4([H])OC=3C([H])=C(O[H])C([H])=C([H])C=3C([H])([H])C4([H])[H]	46500	no meaning	24.2	[89]

ID	SMILES	Ki [nM][a]			Ref.
		D1R	D2R	D3R	
			-ful activity		
**SC236**	[Cl]C=1C([H])=C([H])C(=C([H])C=1[H])N2C([H])([H])C([H])([H])N(C([H])([H])C2([H])[H])C([H])([H])C([H])([H])C([H])([H])C4=NC=3C([H])=C([H])C([H])=C([H])C=3[S]4	1273	2321	214	[56]
**SC237**	[Cl]C=1C([H])=C([H])C(=C([H])C=1[H])N2C([H])([H])C([H])([H])N(C([H])([H])C2([H])[H])C([H])([H])C([H])([H])C([H])([H])C4=NC=3C([H])=C([H])C([H])=C([H])C=3O4	1172	3962	180	
**SC238**	[F]C([F])([F])C=3C([H])=C([H])C(OC([H])([H])C([H])([H])C([H])([H])N2C([H])([H])C([H])([H])N(/C1=N/C([H])=C([H])C([H])=C1[H])C([H])([H])C2([H])[H])=C([H])C=3[H]	2344	1092	355	
**SC239**	[Cl]C=1C([H])=C([H])C(=C([H])C=1[H])N2C([H])([H])C([H])([H])N(C([H])([H])C2([H])[H])C([H])([H])C([H])([H])C([H])([H])C([H])([H])C4=NC=3C([H])=C([H])C([H])=C([H])C=3O4	7939	3108	119	
**SC240**	[H]C=1C([H])=C([H])C(=C([H])C=1[H])C([H])([H])C([H])([H])[C@@]2([H])OC([H])([H])C([H])([H])N(C2([H])[H])C([H])([H])C4=NC=3C([H])=C([H])C([H])=C([H])C=3N4[H]	100000	100000	10.8	[115]
**SC241**	[H]C=1C([H])=C([H])C(=C([H])C=1[H])C([H])([H])C([H])([H])[C@]2([H])OC([H])([H])C([H])([H])N(C2([H])[H])C([H])([H])C4=NC=3C([H])=C([H])C([H])=C([H])C=3N4[H]	100000	100000	25.9	
**SC242**	[H]C=1C([H])=C([H])C(=C([H])C=1[H])C([H])([H])C([H])([H])[C@@]2([H])OC([H])([H])C([H])([H])N(C2([H])[H])C([H])([H])C4=NC=3C([H])=C([H])C([H])=C([H])C=3N4[H]	100000	100000	15.7	
**SC243**	[H]C=1C([H])=C([H])C(=C([H])C=1[H])C([H])([H])C([H])([H])C([H])([H])N(C([H])([H])C([H])([H])C=3C([H])=C([H])C=2OC([H])([H])OC=2C=3[H])C([H])([H])C([H])([H])C=4C([H])=C([H])C(OC([H])([H])[H])=C(OC([H])([H])[H])C=4[H]	1682	1729	498	[116]
**SC244**	[H]C=1C([H])=C([H])C(=C([H])C=1[H])C([H])([H])C([H])([H])C([H])([H])OC=2C([H])=C([H])C(=C([H])C=2OC([H])([H])[H])C([H])([H])C([H])([H])N([H])C([H])([H])[C@@]([H])(O[H])C=3C([H])=C([H])C(O[H])=C(O[H])C=3[H]	2900	1900	660	[96]
**SC245**	O=C2C([H])=C([H])C=1/C(=C(/[H])C([H])=C(O[H])C=1N2[H])[C@]([H])(O[H])C([H])([H])N([H])C([H])([H])C([H])([H])C=4C([H])=C([H])C(OC([H])([H])C([H])([H])C([H])([H])C=3C([H])=C([H])C([H])=C([H])C=3[H])=C(OC([H])([H])[H])C=4[H]	6800	2900	790	
**SC246**	[H]/C2=C(\[H])C(O[H])=C1OC([H])([H])C([H])([H])O/C1=C2\N3C([H])([H])C([H])([H])N(C([H])([H])C3([H])[H])C([H])([H])[H]	7700	3200	790	[99]
**SC247**	[H]/C2=C(\[H])C(O[H])=C1OC([H])([H])C([H])([H])O/C1=C2\N3C([H])([H])C([H])([H])N([H])C([H])([H])C3([H])[H]	6000	6900	110	
**SC248**	O=C1N([H])C2=C(OC1([H])[H])C(=C([H])C([H])=C2O[H])N3C([H])([H])C([H])([H])C([H])([H])N([H])C([H])([H])C3([H])[H]	20000	14000	510	
**SC249**	O=C1N([H])C=2C(OC1([H])[H])=C(C([H])=C([H])C=2O[H])N3C([H])([H])C([H])([H])C([H])([H])N(C([H])([H])C3([H])[H])C([H])([H])[H]	20000	6400	42	

ID	SMILES	Ki [nM][a]			Ref.
		D1R	D2R	D3R	
**SC250**	O=C2C([H])=C([H])C=1/C(=C(/[H])C([H])=C(O[H])C=1N2[H])N3C([H])([H])C([H])([H])N(C([H])([H])C3([H])[H])C([H])([H])C([H])([H])C([H])([H])[H]	20000	1900	420	[100]
**SC251**	O=C1N([H])C2=C(OC1([H])[H])C(=C([H])C([H])=C2O[H])N3C([H])([H])C([H])([H])N([H])C([H])([H])C3([H])[H]	20000	8300	160	
**SC252**	O=C(/C1=N/N2/C(=C1/[H])C([H])=C([H])C([H])=C2[H])N([H])C([H])([H])C([H])([H])C([H])([H])C([H])([H])N5C([H])([H])C([H])([H])[N+]4(C=3C([H])=C([H])C([H])=C([H])C=3OC([H])([H])C4([H])[H])C([H])([H])C5([H])[H]	5700	6300	200	
**SC253**	O=C(C=2[S]C=1C([H])=C([H])C([H])=C([H])C=1C=2[H])N([H])C([H])([H])C([H])([H])C([H])([H])C([H])([H])N4C([H])([H])C([H])([H])N(/C3=N/C(=N/C(=C3/[H])C([F])([F])[F])C(C([H])([H])[H])(C([H])([H])[H])C([H])([H])[H])C([H])([H])C4([H])[H]	10000	4000	2.4	
**SC254**	[H]/C1=C(\[H])C(=C([H])C(O[H])=C1[H])[C@@]2([H])C([H])([H])C([H])([H])N(C2([H])[H])C([H])([H])C([H])([H])C([H])([H])C([H])([H])C([H])([H])C([H])([H])[H]	1278	3555	162	[101]
**SC255**	O=C(/C1=C(\[H])C([H])=NC=2C([H])=C([H])C([H])=C([H])C1=2)N([H])C([H])([H])C([H])([H])C([H])([H])C([H])([H])N4C([H])([H])C3=C([H])C(O[H])=C(OC([H])([H])[H])C([H])=C3C([H])([H])C4([H])[H]	1810	inactive	22	[117]
**SC256**	O=C(C=1C([H])=C([H])C([I])=C([H])C=1[H])N([H])C([H])([H])C([H])([H])C([H])([H])C([H])([H])N3C([H])([H])C2=C([H])C(O[H])=C(OC([H])([H])[H])C([H])=C2C([H])([H])C3([H])[H]	1600	inactive	10	
**SC257**	O=C(C=1C([H])=C([H])C(=C([H])C=1[H])C=2C([H])=C([H])[S]C=2[H])N([H])C([H])([H])C([H])([H])C([H])([H])C([H])([H])N4C([H])([H])C([H])([H])N(C=3C([H])=C([Cl])C([H])=C([Cl])C=3[H])C([H])([H])C4([H])[H]	10000	2049	0.44	[103]
**SC258**	O=C(C=2C([H])=C([H])C=1C([H])=C([H])C([H])=C([H])C=1C=2[H])N([H])C([H])([H])C([H])([H])C([H])([H])C([H])([H])N(C([H])([H])[C@]3([H])C([H])([H])[C@@]3([H])C=4C([H])=C([H])C([H])=C([F])C=4[H])C([H])([H])C([H])([H])C([H])([H])[H]	1308	4823	26.7	[104]
**SC259**	[F]C=1C([H])=C([H])C(OC([H])([H])[H])=C(C=1[H])[C@@]2([H])C([H])([H])[C@@]2([H])C([H])([H])N(C([H])([H])[C@]3([H])C([H])([H])C3([H])[H])C([H])([H])C([H])([H])C([H])([H])[S]/C5=N/N=C(/C=4C([H])=C([H])C([H])=C([H])C=4[H])N5C([H])([H])[H]	1078	5000	64.6	
**SC260**	O=C(C=1C([H])=C([H])C(=C([H])C=1[H])C2=NC([H])=C([H])C([H])=C2[H])N([H])C([H])([H])C([H])([H])C([H])([H])C([H])([H])N(C([H])([H])[C@]3([H])C([H])([H])[C@@]3([H])C=4C([H])=C([F])C([H])=C([H])C=4OC([H])([H])[H])C([H])([H])C([H])([H])C([H])([H])[H]	1758	3144	4	
**SC261**	[F]C=1C([H])=C([H])C(OC([H])([H])[H])=C(C=1[H])[C@@]2([H])C([H])([H])[C@]2([H])C([H])([H])N(C([H])([H])C([H])([H])C([H])([H])[H])C([H])([H])C([H])([H])C([H])([H])[S]/C4=N/N=C(/C=3C([H])=C([H])C([H])=C([H])C=3[H])N4C([H])([H])[H]	1668	1161.4	87.1	
**SC262**	O=C(C=2C([H])=C([H])C=1C([H])=C([H])C([H])=C([H])C=1C=2[H])N([H])C([H])([H])C([H])([H])C([H])([H])C([H])([H])N(C([H])([H])[C@]3([H])C([H])([H])[C@@]3([H])C=4C([H])=C([H])C(	1711	5000	43	

ID	SMILES	Ki [nM][a]			Ref.
		D1R	D2R	D3R	
	=C([H])C=4[H])C([F])([F])[F])C([H])([H])C([H])([H])C([H])([H])[H]				
**SC263**	O=C(C=1C([H])=C([H])C(=C([H])C=1[H])C2=NC([H])=C([H])C([H])=C2[H])N([H])C([H])([H])C([H])([H])C([H])([H])C([H])([H])N(C([H])([H])[C@]3([H])C([H])([H])[C@@]3([H])C=4C([H])=C([H])C([H])=C([F])C=4[H])C([H])([H])C([H])([H])C([H])([H])[H]	2906	5000	45.4	
**SC264**	O=C(/C2=C(\[H])C=1C([H])=C([H])C([H])=C([H])C=1N2[H])N([H])C([H])([H])C([H])([H])C([H])([H])C([H])([H])N(C([H])([H])[C@]3([H])C([H])([H])[C@@]3([H])C=4C([H])=C([H])C([H])=C([Cl])C=4[H])C([H])([H])C([H])([H])C([H])([H])[H]	992	3494	11.1	
**SC265**	O=C(C=2C([H])=C([H])C=1C([H])=C([H])C([H])=C([H])C=1C=2[H])N([H])C([H])([H])C([H])([H])C([H])([H])C([H])([H])N(C([H])([H])[C@]3([H])C([H])([H])[C@@]3([H])C=4C([H])=C([F])C([H])=C([H])C=4OC([H])([H])[H])C([H])([H])C([H])([H])C([H])([H])[H]	967	1083.9	14.5	
**SC266**	O=C(C=1C([H])=C([H])C(=C([H])C=1[H])C2=NC([H])=C([H])C([H])=C2[H])N([H])C([H])([H])C([H])([H])C([H])([H])C([H])([H])N(C([H])([H])[C@]3([H])C([H])([H])[C@@]3([H])C=4C([H])=C([Cl])C([H])=C([H])C=4OC([H])([H])[H])C([H])([H])C([H])([H])C([H])([H])[H]	5000	2105	39.4	
**SC267**	O=C(C=2C([H])=C([H])C=1C([H])=C([H])C([H])=C([H])C=1C=2[H])N([H])C([H])([H])C([H])([H])C([H])([H])C([H])([H])N(C([H])([H])[C@]3([H])C([H])([H])[C@@]3([H])C=4C([H])=C([Cl])C([H])=C([H])C=4OC([H])([H])[H])C([H])([H])C([H])([H])C([H])([H])[H]	930	1116	26.9	
**SC268**	O=C(C=1C([H])=C([H])C(=C([H])C=1[H])C2=NC([H])=C([H])C([H])=C2[H])N([H])C([H])([H])C([H])([H])C([H])([H])C([H])([H])N(C([H])([H])[C@]3([H])C([H])([H])[C@@]3([H])C=4C([H])=C([H])C([F])=C([H])C=4[H])C([H])([H])C([H])([H])C([H])([H])[H]	3631	5000	106.3	
**SC269**	O=C(C=1C([H])=C([H])C(=C([H])C=1[H])C2=NC([H])=C([H])C([H])=C2[H])N([H])C([H])([H])C([H])([H])C([H])([H])C([H])([H])N(C([H])([H])[C@]3([H])C([H])([H])[C@@]3([H])C=4C([H])=C([H])C(=C([H])C=4[H])C([F])([F])[F])C([H])([H])C([H])([H])C([H])([H])[H]	1423	5000	23.4	
**SC270**	O=C(C=1C([H])=C([H])C(=C([H])C=1[H])C2=NC([H])=C([H])C([H])=C2[H])N([H])C([H])([H])C([H])([H])C([H])([H])C([H])([H])N(C([H])([H])[C@]3([H])C([H])([H])[C@@]3([H])C=4C([H])=C([F])C([H])=C([H])C=4OC([H])([H])[H])C([H])([H])C([H])([H])C([H])([H])[H]	4898	1349	4.1	
**SC271**	O=C(C=1C([H])=C([H])C(=C([H])C=1[H])C2=NC([H])=C([H])C([H])=C2[H])N([H])C([H])([H])C([H])([H])C([H])([H])C([H])([H])N(C([H])([H])[C@@]3([H])C([H])([H])[C@]3([H])C=4C([H])=C([H])C([H])=C([Cl])C=4[Cl])C([H])([H])C([H])([H])C([H])([H])[H]	2344	1023	5.3	
**SC272**	O=C(C=2C([H])=C([H])C=1C([H])=C([H])C([H])=C([H])C=1C=2[H])N([H])C([H])([H])C([H])([H])C([H])([H])C([H])([H])N(C([	940	4012	15.5	

ID	SMILES	Ki [nM][a]			Ref.
		D1R	D2R	D3R	
	H])([H])[C@]3([H])C([H])([H])[C@@]3([H])C=4C([H])=C([H])C([H])=C([Cl])C=4[H])C([H])([H])C([H])([H])C([H])([H])[H]				
**SC273**	O=C(C=1C([H])=C([H])C(=C([H])C=1[H])C2=NC([H])=C([H])C([H])=C2[H])N([H])C([H])([H])C([H])([H])C([H])([H])C([H])([H])N(C([H])([H])[C@]3([H])C([H])([H])[C@@]3([H])C=4C([H])=C([H])C([H])=C([Cl])C=4[Cl])C([H])([H])C([H])([H])C([H])([H])[H]	1647	2362	1.2	
**SC274**	O=C(C=1C([H])=C([H])C(=C([H])C=1[H])C2=NC([H])=C([H])C([H])=C2[H])N([H])C([H])([H])C([H])([H])C([H])([H])C([H])([H])N(C([H])([H])[C@]3([H])C([H])([H])[C@@]3([H])C=4C([H])=C([H])C([Cl])=C([H])C=4[H])C([H])([H])C([H])([H])C([H])([H])[H]	1308	5000	17.6	
**SC275**	O=C(C=2C([H])=C([H])C=1C([H])=C([H])C([H])=C([H])C=1C=2[H])N([H])C([H])([H])C([H])([H])C([H])([H])C([H])([H])N(C([H])([H])[C@]3([H])C([H])([H])[C@@]3([H])C=4C([H])=C([H])C([F])=C([H])C=4[H])C([H])([H])C([H])([H])C([H])([H])[H]	992	5623	55.8	
**SC276**	O=C(/C2=C(\[H])C=1C([H])=C([H])C([H])=C([H])C=1N2[H])N([H])C([H])([H])C([H])([H])C([H])([H])C([H])([H])N(C([H])([H])[C@]3([H])C([H])([H])[C@@]3([H])C=4C([H])=C([H])C([F])=C([H])C=4[H])C([H])([H])C([H])([H])C([H])([H])[H]	1457	5934	45.7	
**SC277**	O=C(C=2C([H])=C([H])C=1C([H])=C([H])C([H])=C([H])C=1C=2[H])N([H])C([H])([H])C([H])([H])C([H])([H])C([H])([H])N(C([H])([H])[C@@]3([H])C([H])([H])[C@]3([H])C=4C([H])=C([H])C([H])=C([Cl])C=4[Cl])C([H])([H])C([H])([H])C([H])([H])[H]	1047	1148	20.8	
**SC278**	O=C(/C2=C(\[H])C=1C([H])=C([H])C([H])=C([H])C=1N2[H])N([H])C([H])([H])C([H])([H])C([H])([H])C([H])([H])N(C([H])([H])[C@]3([H])C([H])([H])[C@@]3([H])C=4C([H])=C([H])C(=C([H])C=4[H])C([F])([F])[F])C([H])([H])C([H])([H])C([H])([H])[H]	1278	5000	33.1	
**SC279**	O=C(/C2=C(\[H])C=1C([H])=C([H])C([H])=C([H])C=1N2[H])N([H])C([H])([H])C([H])([H])C([H])([H])C([H])([H])N(C([H])([H])[C@]3([H])C([H])([H])[C@@]3([H])C=4C([H])=C([H])C([H])=C([F])C=4[H])C([H])([H])C([H])([H])C([H])([H])[H]	1105	3494	18.8	
**SC280**	O=C(C=1C([H])=C([H])C(=C([H])C=1[H])C2=NC([H])=C([H])C([H])=C2[H])N([H])C([H])([H])C([H])([H])C([H])([H])C([H])([H])N(C([H])([H])[C@@]3([H])C([H])([H])[C@]3([H])C=4C([H])=C([F])C([H])=C([H])C=4OC([H])([H])[H])C([H])([H])C([H])([H])C([H])([H])[H]	1071	1230	3.8	
**SC281**	O=C(C=2C([H])=C([H])C=1C([H])=C([H])C([H])=C([H])C=1C=2[H])N([H])[C@]3([H])C([H])([H])[C@](O[H])(C3([H])[H])C([H])([H])C([H])([H])N([C@]5([H])C([H])([H])C=4[S]C(=NC=4C([H])([H])C5([H])[H])N([H])[H])C([H])([H])C([H])([H])C([H])([H])[H]	inactive	3902	30	[105]
**SC282**	[H]C=4C([H])=C3C1=C(C([H])=C([H])C=2N=C([S]C1=2)N([H])[H])C([H])([H])[C@]5([H])C3=C(C=4[H])C([H])([H])C([H])([H])N5C([H])([H])[H]	1150	1740	381	[118]
**SC283**	[H]/C1=C(\[H])C(=NC([H])=C1[C@@]2([H])OC([H])([H])[C@@]([H])(N(C2([H])[H])C([H])([H])C([H])([H])C([H])([H])[H])C([H])([H])[H])N([H])[H]	100000	1740	185	[106]

ID	SMILES	Ki [nM][a]			Ref.
		D_1R	D_2R	D_3R	
**SC284**	O=C(/C2=C(\[H])C=1C([H])=C([H])C([H])=C([H])C=1N2[H])N([H])C([H])([H])C([H])([H])[C@@]([H])(O[H])C([H])([H])N3C([H])([H])[C@@]([H])(OC([H])([H])[C@]3([H])C([H])([H])[H])C=4C([H])=C([H])C(=NC=4[H])N([H])[H]	100000	1010	423	

[a]All KI values retrieved from ChEMBL were determined in vitro utilizing radio-ligand binding assays.

2. Molecular Dynamics Simulation (MDS) – methodological overview

A summary of the minimization process settings is shown in Table S5. An overview of all parameters is given in Table S6. A summary of the Standard Dynamics Cascade as well as a detailed listing of all parameters is shown in Table S7 and Table S8. A Spherical Cutoff method is used for electrostatics.

Table S5. Summary of the Minimization process for the D_2R structure.

Potential Energy [kcal/mol]	Van der Waals Energy [kcal/mol]	Electrostatic Energy [kcal/mol]	Initial RMS Gradient [kcal/mol*A]	Final RMS Gradient [kcal/mol*A]	Minimizatio Criteria
-73078.46743	-7856.40360	-74320.37289	1.16878	0.47676	CONJUG> Minimization exiting with number of steps limit (200) exceeded

**Forcefield**	CHARMm
**Initial Potential Energy [kcal/mol]**	-70950.65436

Table S6. Detailed overview of the settings used during the Minimization process of the D2R structure.

**Minimization**	
**Parameter**	**Setting**
Algorithm	Smart Minimizer
Max Steps	200
RMS Gradient	0.1
Energy Change	00
Save Results Frequency	0
Implicit Solvent Model	None
Dielectric Constant	1
Implicit Solvent Dilectric Constant	80
Generalized Born Lambda Constant	-
Minimum Hydrogen Radius	0.8
Use Non-polar surface	True
Non-polar Surface Constant	0.92
Non-Polar Surface Coefficient	0.00542
Salt Concentration	0.0
Input Atomic Radii	van der Waals Radii
Use Molecular Surface	True
Nonbond List Radius	14.0
Nonbond Higher Cutoff Distance	12.0
Nonbond Lower Cutoff Distance	10.0
Electrostatics	Automatic
Kappa	0.34
Order	4
Minimization Constraints	-
Apply SHAKE Constraint	False

Number of Processors	1

Table S7. Summary of the Standard Dynamics Cascade process for the D2R structure.

End Time [ps]	Initial Potential Energy [kcal/mol]	Total Energy [kcal/mol]	Potential Energy [kcal/mol]	Kinetic Energy [kcal/mol]	Temperature [K]	Van der Waals Energy [kcal/mol]	Electrostatic Energy [kcal/mol]	Initial RMS Gradient [kcal/mol*A]	Final RMS Gradient [kcal/mol*A]
-	-72896.919	-	-72964.947	-	-	-7836.896	-74028.920	0.972	0.650
-	-72964.947	-	-74257.521	-	-	-7843.438	-75322.971	0.650	0.075
4000	-74257.521	-48876.612	-62155.902	13279.289	302.709	-6715.957	-74737.941	3.628	19.257
1004	-62155.902	-54734.887	-67927.536	13192.649	300.734	-6952.978	-80960.394	19.257	19.232
1014	-67927.536	-54865.097	-68117.446	13252.349	302.094	-6758.705	-81034.075	19.232	19.127

Stage	Forcefield	Start Time [ps]
Minimization	CHARMm	-
Minimization 2	CHARMm	-
Heating	CHARMm	0
Equilibration	CHARMm	4
Production	CHARMm	1004

Table S8. Detailed overview of the settings used during the Standard Dynamics Cascade process of the D_2R structure.

**Standard Dynamics Cascade**	
**Minimization**	
**Parameter**	**Setting**
Algorithm	Steepest Descent
Max Steps	1000
RMS Gradient	1.0
Constraints	-
**Minimization 2**	
**Parameter**	**Setting**
Algorithm	Adopted Basis NR
Max Steps	2000
RMS Gradient	0.1
Constraints	-
**Heating**	
**Parameter**	**Setting**
Simulation Time [ps]	4
Time Step [fs]	2
Initial Temperature	50.0
Target Temperature	300.0
Adjust Velocity Frequency	50
Save Results Interval [ps]	2
Constraints	-

Equilibration	
**Parameter**	**Setting**
Simulation Time [ps]	1000
Time Step [fs]	2
Target Temperature	300.0
Adjust Velocity Frequency	50
Save Results Interval [ps]	2
Constraints	-
**Production**	
**Parameter**	**Setting**
Simulation Time [ps]	10
Time Step [fs]	2
Target Temperature	300.0
Temperature Coupling Decay Time	5.0
Save Result Interval [ps]	2
Save Result File	True
Constraints	-
Type	NVT
TMass	1000.0
PMass	1000.0
PGamma	25.0
Reference Pressure	1.0
**Implicit Solvent Model**	
**Parameter**	**Setting**
Implicit Solvent Model	None
Dielectric Constant	1
Implicit Solvent Dielectric Constant	80
Generalized Born Lambda Constant	-
Minimum Hydrogen Radius	0.8
Use Non-polar surface	True
Non-polar Surface Constant	0.92
Non-Polar Surface Coefficient	0.00542
Salt Concentration	0.0
Input Atomic Radii	Van der Waals Radii

Use Molecular Surface	Ture
Nonbond List Radius	14.0
Nonbond Higher Cutoff Distance	12.0
Nonbond Lower Cutoff Distance	10.0
Electrostatics	Automatic
Kappa	0.34
Order	4
Dynamics Integrator	Leapfrog Verlet
Apply SHAKE Constraint	True
Random Number Seed	314159 314159 314159 314159
Number of Processors	1

3. In vitro screening– overview of 2D structures and NDF values of all compounds

Table S9. Overview of all compounds selected for further in vitro investigations. 2D structures and NDF ± standard deviation (SD) are shown for each compound (n = 4). All NDF values for D_2R were obtained in the previously published article of Zell et al [42]. NDF, normalized decreased fluorescence.

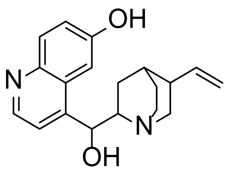

SC285
NDF (D_1R) = 0.83 ± 0.06
NDF (D_2R) = 1.13 ± 0.37
NDF (D_3R) = 1.17 ± 0.11

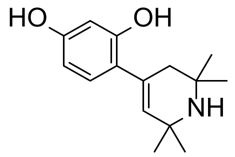

SC286
NDF (D_1R) = 0.88 ± 0.20
NDF (D_2R) = 1.05 ± 0.45
NDF (D_3R) = 1.04 ± 0.10

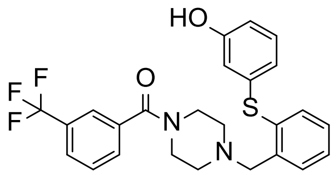

SC287
NDF (D_1R) = 0.91 ± 0.21
NDF (D_2R) = 0.90 ± 0.25
NDF (D_3R) = 1.00 ± 0.17

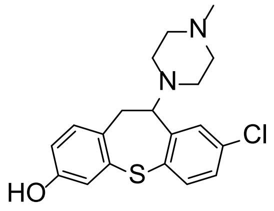

2
NDF (D_1R) = 5.41 ± 1.97
NDF (D_2R) = 40.41 ± 1.39
NDF (D_3R) = 4.16 ± 1.08

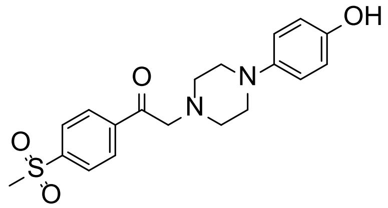

SC288
NDF (D_1R) = 3.21 ± 3.27
NDF (D_2R) = 1.01 ± 0.38
NDF (D_3R) = 1.58 ± 0.58

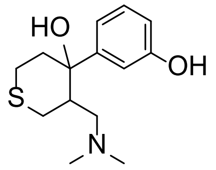

SC289
NDF (D_1R) = 1.66 ± 1.16
NDF (D_2R) = 0.90 ± 0.30
NDF (D_3R) = 1.24 ± 0.25

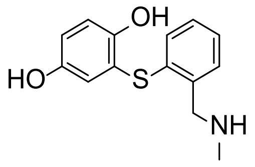

SC290
NDF (D_1R) = 3.50 ± 3.16
NDF (D_2R) = 0.91 ± 0.36
NDF (D_3R) = 1.81 ± 1.04

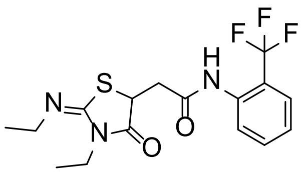

SC291
NDF (D_1R) = 0.81 ± 0.08
NDF (D_2R) = 0.84 ± 0.23
NDF (D_3R) = 1.02 ± 0.24

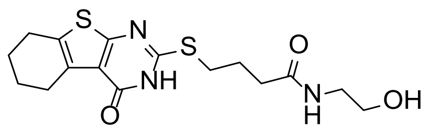

SC292
NDF (D_1R) = 0.80 ± 0.21
NDF (D_2R) = 0.95 ± 0.43
NDF (D_3R) = 0.92 ± 0.13

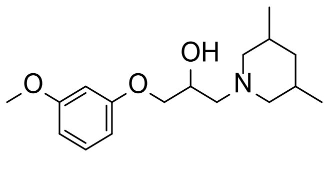

SC293
NDF (D_1R) = 0.85 ± 0.14
NDF (D_2R) = 1.02 ± 0.39
NDF (D_3R) = 1.04 ± 0.11

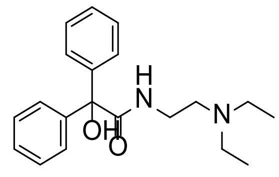

SC294
NDF (D_1R) = 2.72 ± 2.66
NDF (D_2R) = 1.01 ± 0.50
NDF (D_3R) = 1.49 ± 0.54

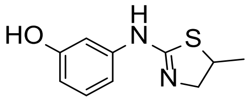

SC295
NDF (D_1R) = 1.08 ± 0.42
NDF (D_2R) = 0.95 ± 0.43
NDF (D_3R) = 1.26 ± 0.25

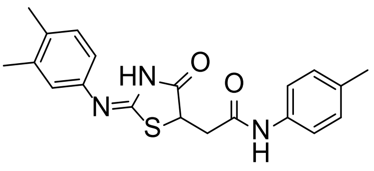

SC296
NDF (D_1R) = 1.02 ± 0.36
NDF (D_2R) = 0.94 ± 0.32
NDF (D_3R) = 0.93 ± 0.12

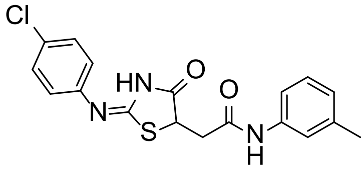

SC297
NDF (D_1R) = 0.89 ± 0.24
NDF (D_2R) = 0.96 ± 0.38
NDF (D_3R) = 1.04 ± 0.11

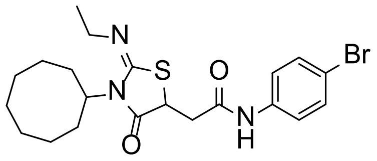

SC298
NDF (D_1R) = 1.21 ± 0.43
NDF (D_2R) = 1.00 ± 0.35
NDF (D_3R) = 0.85 ± 0.13

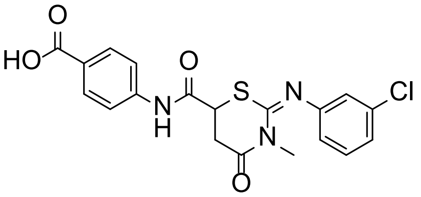

SC299
NDF (D_1R) = 0.94 ± 0.13
NDF (D_2R) = 0.82 ± 0.25
NDF (D_3R) = 1.05 ± 0.36

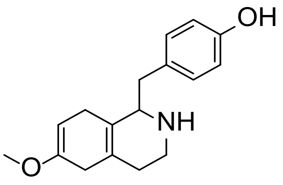

SC300
NDF (D_1R) = 2.72 ± 0.84
NDF (D_2R) = 1.14 ± 0.56
NDF (D_3R) = 2.16 ± 0.40

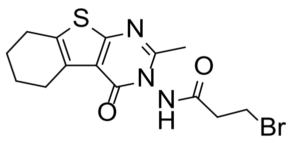

SC301
NDF (D_1R) = 0.78 ± 0.22
NDF (D_2R) = 0.99 ± 0.35
NDF (D_3R) = 0.97 ± 0.11

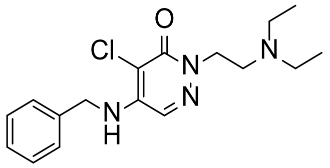

SC302
NDF (D_1R) = 1.04 ± 0.28
NDF (D_2R) = 0.99 ± 0.39
NDF (D_3R) = 1.26 ± 0.14

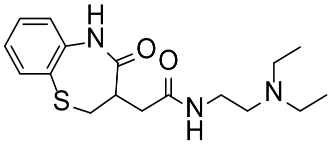

SC303
NDF (D_1R) = 1.00 ± 0.33
NDF (D_2R) = 1.02 ± 0.36
NDF (D_3R) = 1.50 ± 0.24

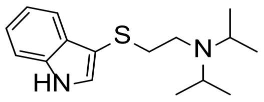

SC304
NDF (D_1R) = 0.88 ± 0.17
NDF (D_2R) = 0.98 ± 0.32
NDF (D_3R) = 1.20 ± 0.11

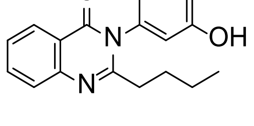

SC305
NDF (D_1R) = 0.90 ± 0.26
NDF (D_2R) = 0.93 ± 0.29
NDF (D_3R) = 1.03 ± 0.14

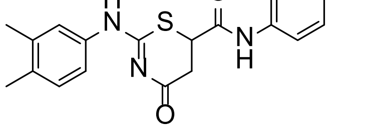

SC306
NDF (D_1R) = 1.05 ± 0.44
NDF (D_2R) = 0.94 ± 0.36
NDF (D_3R) = 1.09 ± 0.30

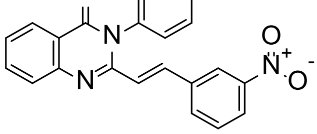

SC307
NDF (D_1R) = 0.93 ± 0.16
NDF (D_2R) = 0.86 ± 0.24
NDF (D_3R) = 0.93 ± 0.15

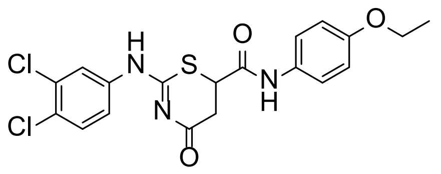

SC308

NDF (D_1R) = 0.87 ± 0.20
NDF (D_2R) = 2.04 ± 1.34
NDF (D_3R) = 1.64 ± 0.29

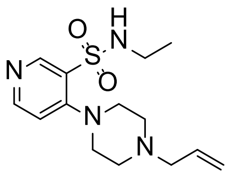

SC309

NDF (D_1R) = 0.96 ± 0.34
NDF (D_2R) = 1.10 ± 0.46
NDF (D_3R) = 2.03 ± 0.18

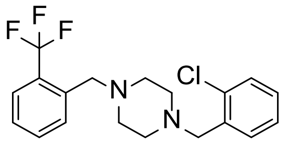

SC310

NDF (D_1R) = 0.99 ± 0.34
NDF (D_2R) = 1.06 ± 0.41
NDF (D_3R) = 1.54 ± 0.33

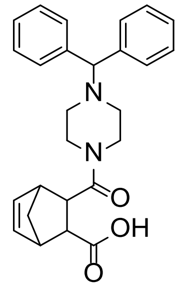

SC311

NDF (D_1R) = 1.09 ± 0.43
NDF (D_2R) = 1.02 ± 0.35
NDF (D_3R) = 1.67 ± 0.44

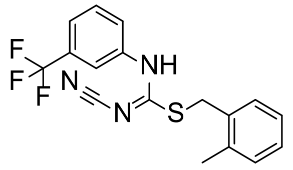

SC312

NDF (D_1R) = 0.97 ± 0.40
NDF (D_2R) = 1.10 ± 0.44
NDF (D_3R) = 1.23 ± 0.27

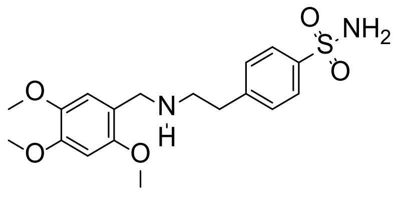

SC313

NDF (D_1R) = 0.88 ± 0.33
NDF (D_2R) = 0.94 ± 0.32
NDF (D_3R) = 1.12 ± 0.15

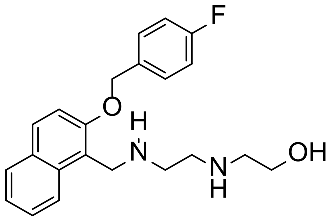

SC314

NDF (D_1R) = 4.17 ± 1.08
NDF (D_2R) = 1.68 ± 0.90
NDF (D_3R) = 3.00 ± 0.61

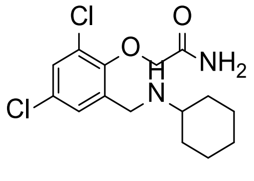

SC315

NDF (D_1R) = 1.18 ± 1.01
NDF (D_2R) = 0.95 ± 0.32
NDF (D_3R) = 1.06 ± 0.20

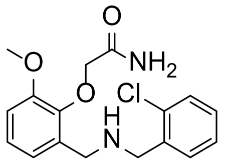

SC316

NDF (D_1R) = 0.93 ± 0.30
NDF (D_2R) = 1.01 ± 0.44
NDF (D_3R) = 1.16 ± 0.16

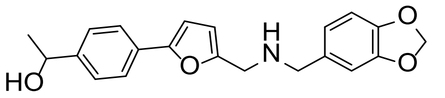

SC317

NDF (D_1R) = 1.06 ± 0.47
NDF (D_2R) = 0.93 ± 0.42
NDF (D_3R) = 1.07 ± 0.15

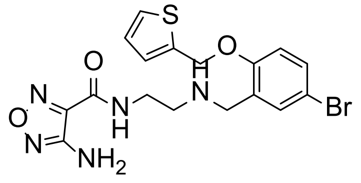

SC318

NDF (D_1R) = 1.81 ± 0.42
NDF (D_2R) = 1.14 ± 0.57
NDF (D_3R) = 2.39 ± 0.48

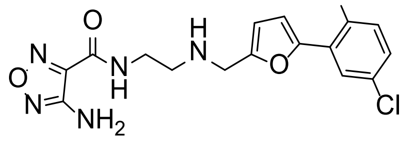

SC319

NDF (D_1R) = 0.89 ± 0.26
NDF (D_2R) = 2.41 ± 2.01
NDF (D_3R) = 3.64 ± 1.08

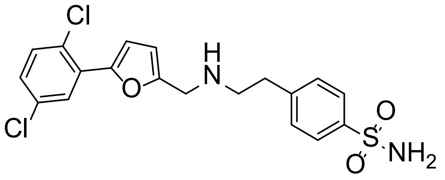

3

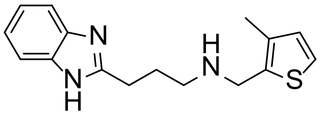

SC320

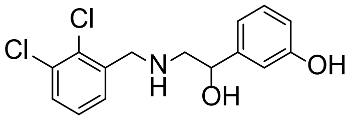

4

NDF (D_1R) = 1.78 ± 1.35
NDF (D_2R) = 3.99 ± 2.58
NDF (D_3R) = 3.96 ± 1.10

NDF (D_1R) = -
NDF (D_2R) = -
NDF (D_3R) = -

NDF (D_1R) = 0.90 ± 0.31
NDF (D_2R) = 0.90 ± 0.38
NDF (D_3R) = 2.75 ± 0.61

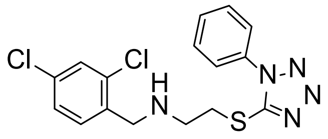

SC321

NDF (D_1R) = 1.00 ± 0.42
NDF (D_2R) = 0.97 ± 0.36
NDF (D_3R) = 1.26 ± 0.24

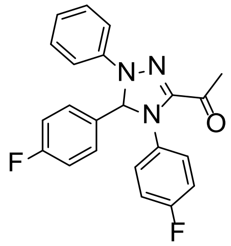

SC322

NDF (D_1R) = 0.86 ± 0.24
NDF (D_2R) = 0.90 ± 0.25
NDF (D_3R) = 1.01 ± 0.23

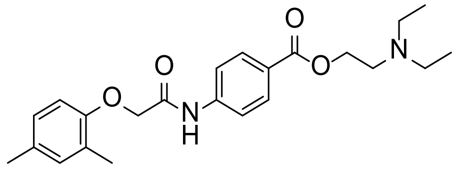

SC323

NDF (D_1R) = 1.43 ± 0.58
NDF (D_2R) = 0.95 ± 0.35
NDF (D_3R) = 1.55 ± 0.23

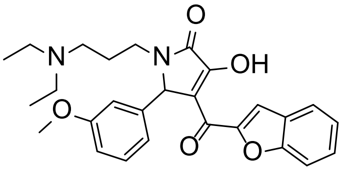

SC324

NDF (D_1R) = -
NDF (D_2R) = -
NDF (D_3R) = -

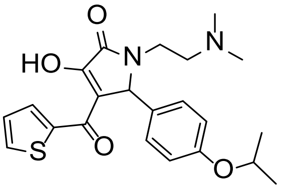

SC325

NDF (D_1R) = 1.02 ± 0.46
NDF (D_2R) = 0.94 ± 0.46
NDF (D_3R) = 1.22 ± 0.27

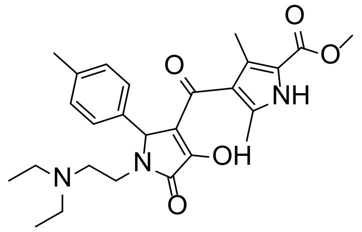

SC326

NDF (D_1R) = 0.87 ± 0.33
NDF (D_2R) = 0.95 ± 0.42
NDF (D_3R) = 1.06 ± 0.36

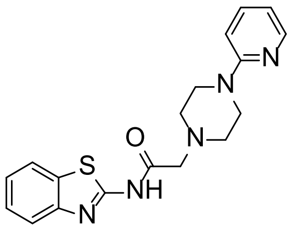

SC327

NDF (D_1R) = 0.92 ± 0.39
NDF (D_2R) = 0.90 ± 0.39
NDF (D_3R) = 1.00 ± 0.20

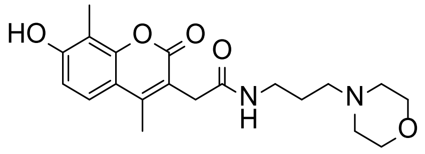

SC328

NDF (D_1R) = 0.43 ± 0.15
NDF (D_2R) = 0.99 ± 0.43
NDF (D_3R) = 1.13 ± 0.31

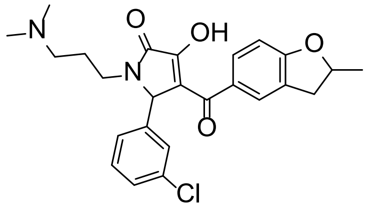

SC329

NDF (D_1R) = -
NDF (D_2R) = -
NDF (D_3R) = -

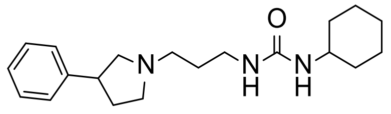

SC330

NDF (D_1R) = 1.88 ± 0.72
NDF (D_2R) = 1.35 ± 0.68
NDF (D_3R) = 3.31 ± 0.92

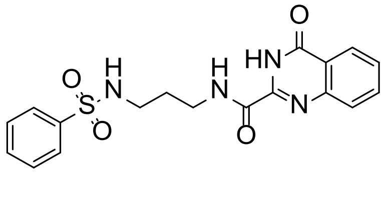

SC331

NDF (D_1R) = 0.95 ± 0.17
NDF (D_2R) = 0.90 ± 0.35
NDF (D_3R) = 1.14 ± 0.40

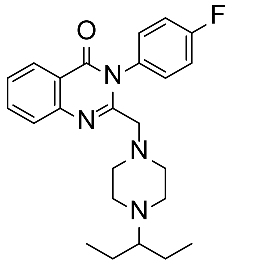

SC332

NDF (D_1R) = 0.97 ± 0.40
NDF (D_2R) = 0.90 ± 0.36
NDF (D_3R) = 1.18 ± 0.28

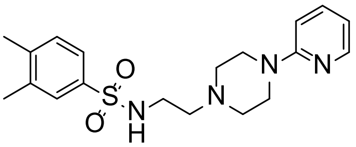

SC333

NDF (D_1R) = 1.13 ± 0.52
NDF (D_2R) = 2.98 ± 1.77
NDF (D_3R) = 2.49 ± 0.47

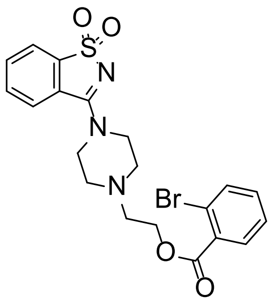

SC334

NDF (D_1R) = 0.95 ± 0.35
NDF (D_2R) = 0.96 ± 0.37
NDF (D_3R) = 1.12 ± 0.30

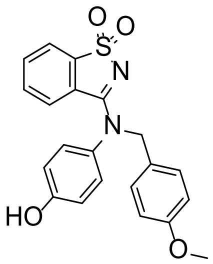

SC335

NDF (D_1R) = 0.93 ± 0.39
NDF (D_2R) = 1.03 ± 0.42
NDF (D_3R) = 1.40 ± 0.54

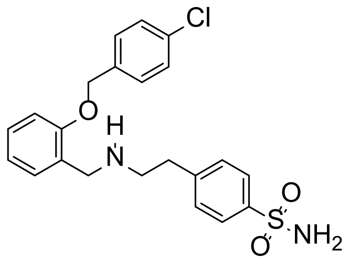

SC336

NDF (D_1R) = 1.46 ± 0.71
NDF (D_2R) = 1.07 ± 0.46
NDF (D_3R) = 2.76 ± 0.63

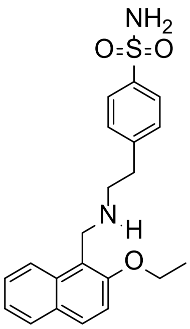

SC337

NDF (D_1R) = 1.12 ± 0.56
NDF (D_2R) = 0.99 ± 0.40
NDF (D_3R) = 1.24 ± 0.22

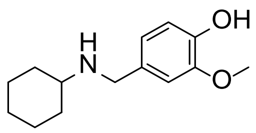

SC338

NDF (D_1R) = 1.14 ± 0.77
NDF (D_2R) = 0.93 ± 0.38
NDF (D_3R) = 1.15 ± 0.35

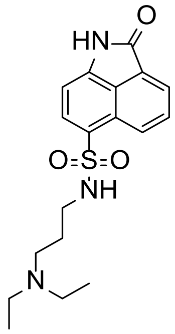

SC339

NDF (D_1R) = 0.59 ± 0.17
NDF (D_2R) = 0.94 ± 0.41
NDF (D_3R) = 1.11 ± 0.48

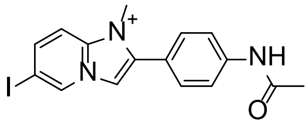

SC340

NDF (D_1R) = 0.83 ± 0.35
NDF (D_2R) = 1.41 ± 1.07
NDF (D_3R) = 1.10 ± 0.29

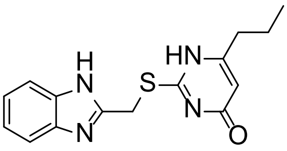

SC341

NDF (D_1R) = 0.83 ± 0.51
NDF (D_2R) = 1.52 ± 1.15
NDF (D_3R) = 0.92 ± 0.14

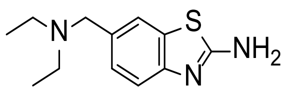

SC342

NDF (D_1R) = 1.24 ± 0.51
NDF (D_2R) = 1.38 ± 0.91
NDF (D_3R) = 1.24 ± 0.29

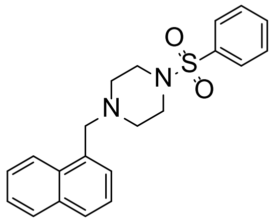

SC343

NDF (D_1R) = 1.36 ± 0.89
NDF (D_2R) = 1.06 ± 0.44
NDF (D_3R) = 1.15 ± 0.32

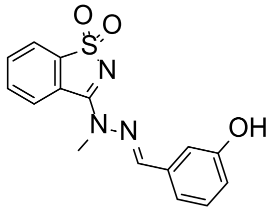

SC344

NDF (D_1R) = 1.09 ± 0.58
NDF (D_2R) = 1.06 ± 0.42
NDF (D_3R) = 0.99 ± 0.13

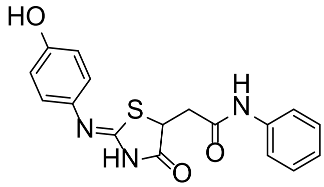

SC345

NDF (D_1R) = 0.93 ± 0.32
NDF (D_2R) = 0.95 ± 0.38
NDF (D_3R) = 1.11 ± 0.36

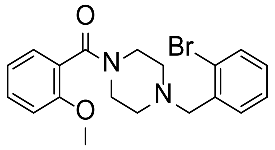

SC346

NDF (D_1R) = 1.04 ± 0.54
NDF (D_2R) = 0.97 ± 0.39
NDF (D_3R) = 1.15 ± 0.37

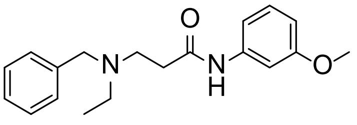

5

NDF (D_1R) = 1.26 ± 0.64
NDF (D_2R) = 15.74 ± 18.15
NDF (D_3R) = 2.65 ± 0.64

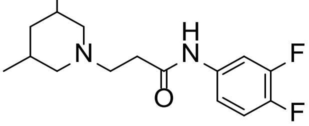

SC347

NDF (D_1R) = 1.12 ± 0.60
NDF (D_2R) = 1.05 ± 0.50
NDF (D_3R) = 1.40 ± 0.33

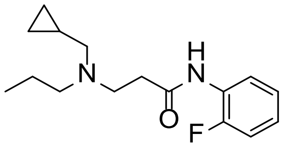

SC348

NDF (D_1R) = 0.89 ± 0.31
NDF (D_2R) = 1.02 ± 0.38
NDF (D_3R) = 1.36 ± 0.36

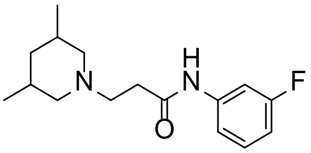

SC349

NDF (D_1R) = 1.12 ± 0.59
NDF (D_2R) = 1.26 ± 0.70
NDF (D_3R) = 1.95 ± 0.47

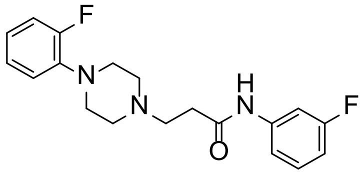

6

NDF (D_1R) = 2.15 ± 1.16
NDF (D_2R) = 8.18 ± 3.62
NDF (D_3R) = 4.21 ± 0.79

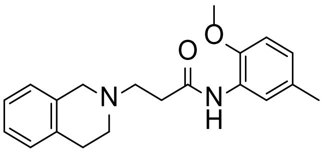

SC350

NDF (D_1R) = 1.19 ± 0.57
NDF (D_2R) = 1.83 ± 0.83
NDF (D_3R) = 3.25 ± 0.47

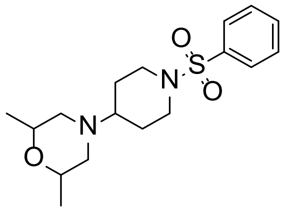

SC351

NDF (D_1R) = 0.93 ± 0.39
NDF (D_2R) = 0.99 ± 0.42
NDF (D_3R) = 1.19 ± 0.45

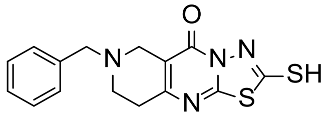

SC352

NDF (D_1R) = 0.91 ± 0.42
NDF (D_2R) = 1.02 ± 0.47
NDF (D_3R) = 1.22 ± 0.38

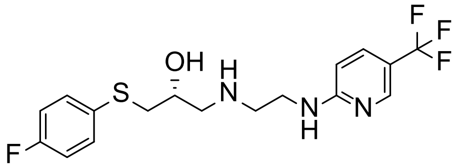

7

NDF (D_1R) = 1.57 ± 0.54
NDF (D_2R) = 10.85 ± 4.93
NDF (D_3R) = 3.79 ± 0.70

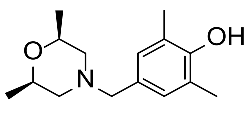

SC353

NDF (D_1R) = 1.08 ± 0.61
NDF (D_2R) = 1.00 ± 0.40
NDF (D_3R) = 1.20 ± 0.43

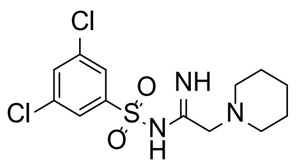

SC354

NDF (D_1R) = 0.98 ± 0.52
NDF (D_2R) = 0.99 ± 0.39
NDF (D_3R) = 1.06 ± 0.24

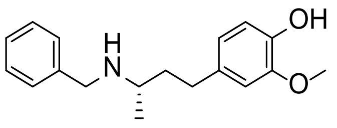

8

NDF (D_1R) = 1.20 ± 0.59
NDF (D_2R) = 1.10 ± 0.52
NDF (D_3R) = 2.63 ± 0.52

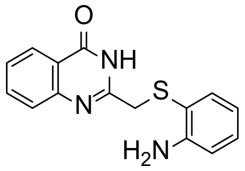

SC355

NDF (D_1R) = 1.34 ± 1.14
NDF (D_2R) = 1.13 ± 0.34
NDF (D_3R) = 1.22 ± 0.35

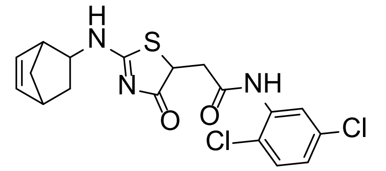

SC356

NDF (D_1R) = 1.28 ± 0.99
NDF (D_2R) = 2.44 ± 1.39
NDF (D_3R) = 1.23 ± 0.29

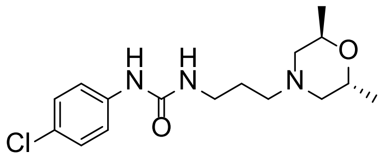

SC357

NDF (D_1R) = 1.05 ± 0.57
NDF (D_2R) = 1.66 ± 1.60
NDF (D_3R) = 2.02 ± 0.83

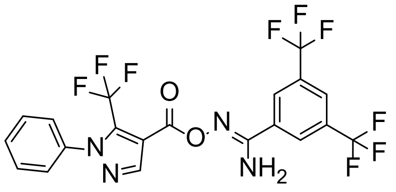

SC358

NDF (D_1R) = 1.33 ± 0.55
NDF (D_2R) = 1.11 ± 0.47
NDF (D_3R) = 1.22 ± 0.53

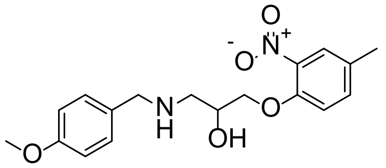

SC359

NDF (D_1R) = 1.28 ± 0.73
NDF (D_2R) = 2.00 ± 0.85
NDF (D_3R) = 2.49 ± 0.74

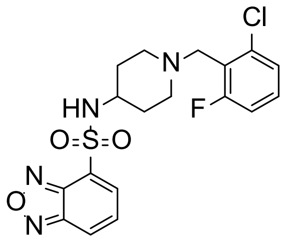

SC360

NDF (D_1R) = 1.05 ± 0.56
NDF (D_2R) = 0.93 ± 0.40
NDF (D_3R) = 1.35 ± 0.33

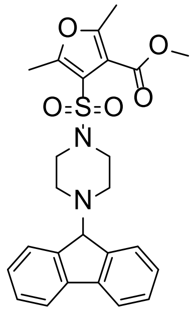

SC361

NDF (D_1R) = 0.96 ± 0.52
NDF (D_2R) = 1.11 ± 0.51
NDF (D_3R) = 1.10 ± 0.24

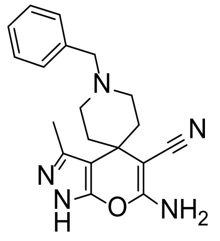

SC362

NDF (D_1R) = 1.05 ± 0.36
NDF (D_2R) = 1.41 ± 0.71
NDF (D_3R) = 2.03 ± 0.45

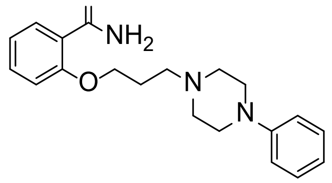

9

NDF (D_1R) = 1.71 ± 0.86
NDF (D_2R) = 22.08 ± 6.62
NDF (D_3R) = 3.97 ± 0.78

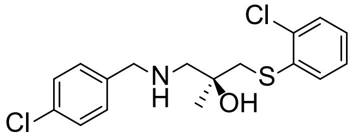

SC363

NDF (D_1R) = 1.11 ± 0.70
NDF (D_2R) = 1.11 ± 0.53
NDF (D_3R) = 1.53 ± 0.21

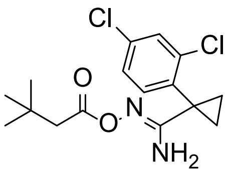

SC364

NDF (D_1R) = 0.92 ± 0.42
NDF (D_2R) = 1.08 ± 0.56
NDF (D_3R) = 1.17 ± 0.37

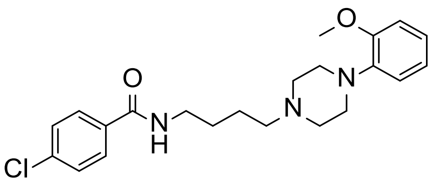

10

NDF (D_1R) = 2.59 ± 1.04
NDF (D_2R) = 22.89 ± 8.41
NDF (D_3R) = 4.09 ± 1.07

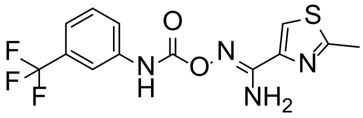

SC365

NDF (D_1R) = 0.99 ± 0.38
NDF (D_2R) = 1.04 ± 0.39
NDF (D_3R) = 1.12 ± 0.14

4. In silico assessment of DR subtype selectivity – a distance-based approach

4.1. ChEMBL validation

Table S10. Summary of the validation of the distance-based in silico approach using the described datasets extracted from ChEMBL. Cons. Gly, conserved glycine residue within the respective SBP. COM, center of mass.

Dataset	Distance (cons. Gly – COM) [Å]			Fold-difference (distance-based)		
	D_1R	D_2R	D_3R	D_1R/D_2R	D_1R/D_3R	D_2R/D_3R
D_2R-selective	17.88	13.59	13.66	1.32	1.31	0.99
D_2like-selective	17.91	12.31	13.11	1.45	1.37	0.94
D_3R-selective	17.79	12.44	13.17	1.43	1.35	0.94

4.2. Retrospective analysis of investigated compounds

Table S11. Summary of the distance-based in silico approach used during the investigation of the binding poses of the selected compounds **1** to **10**. Cpd., compound. Cons. Gly, conserved glycine residue within the respective SBP. COM, center of mass.

Cpd.	Pose rank (out of 30)			Distance (cons. Gly – COM) [Å]			Fold-difference (distance-based)		
	D_1R	D_2R	D_3R	D_1R	D_2R	D_3R	D_1R/D_2R	D_1R/D_3R	D_2R/D_3R
**1**	4th	1st	5th	19.96	17.70	16.70	1.13	1.19	1.06
**2**	1st	4th	1st	19.20	14.47	14.96	1.33	1.28	0.97
**3**	1st	1st	1st	18.16	13.41	14.15	1.35	1.28	0.95
**4**	1st	2nd	1st	17.70	15.42	14.06	1.15	1.26	1.10
**5**	1st	6th	4th	18.73	13.95	14.31	1.34	1.31	0.98
**6**	1st	1st	2nd	17.12	14.77	15.14	1.16	1.13	0.98
**7**	1st	2nd	2nd	18.32	12.99	14.05	1.41	1.30	0.93
**8**	1st	1st	7th	19.27	16.45	15.77	1.17	1.22	1.04
**9**	2nd	2nd	2nd	19.29	12.83	14.07	1.50	1.37	0.91
**10**	1st	1st	1st	18.11	12.84	13.50	1.41	1.34	0.95

5. Molecular dynamics simulation (MDS) – a workflow for the D2R

The MDS was performed following the workflow shown in **Figure S1.**

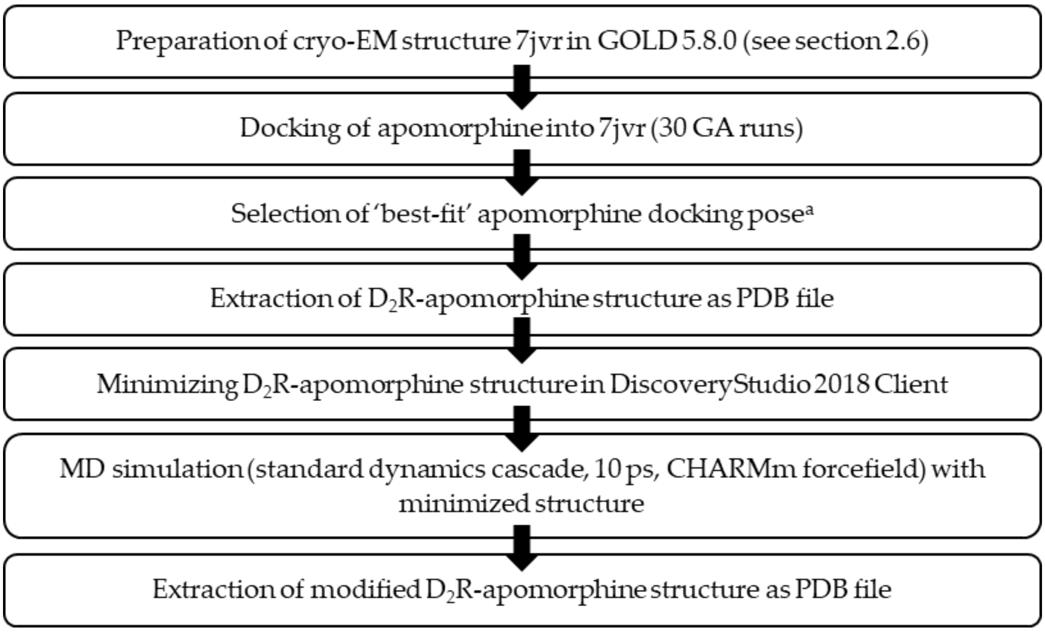

Figure S1. Summary of the utilized approach to modify PDB entry 7jvr [34] using a MDS approach. [a] 'best-fit' docking pose was assessed based on the correct positioning of apomorphine in the related D_1R structure 7jvq [34] with respect to the orientation of the catechol motif towards the serine triade as well as the formation of a salt-bridge with $Asp^{3.32}$.

6. In vitro careening activity of selected compounds

6.1. Summary of in vitro activity (based on NDF values) of control compounds and selected virtual hits

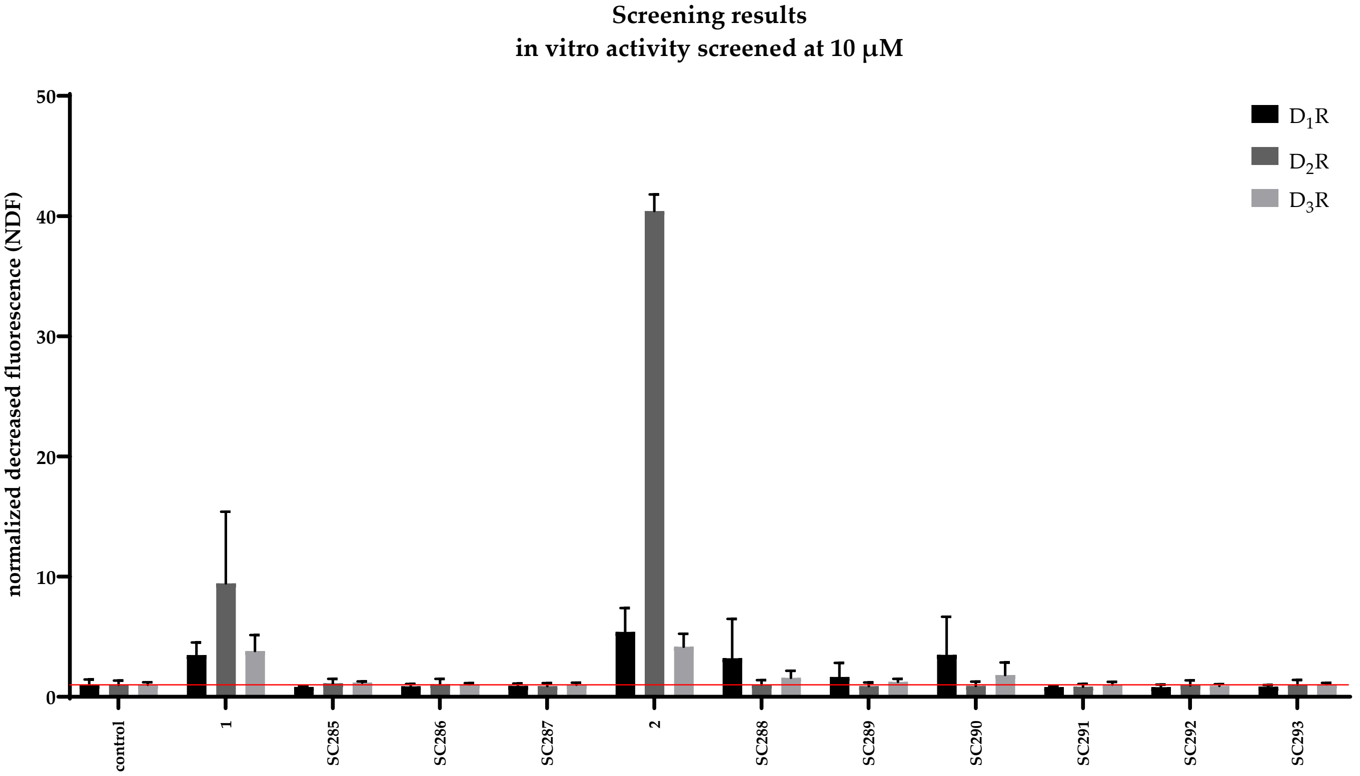

Figure S2. Comparison of biological activity of selected compounds at DR subtypes D_1R (black), D_2R (dark grey) and D_3R (light grey). Compound binding is shown as NDF in comparison to the control. Activities were measured in biological duplicates (two technical replicates each). Error bars show standard deviations (SD). The red line indicates a NDF of 1, thus, an inactive ligand at 10 µM screening concentration. NDF, normalized decreased fluorescence.

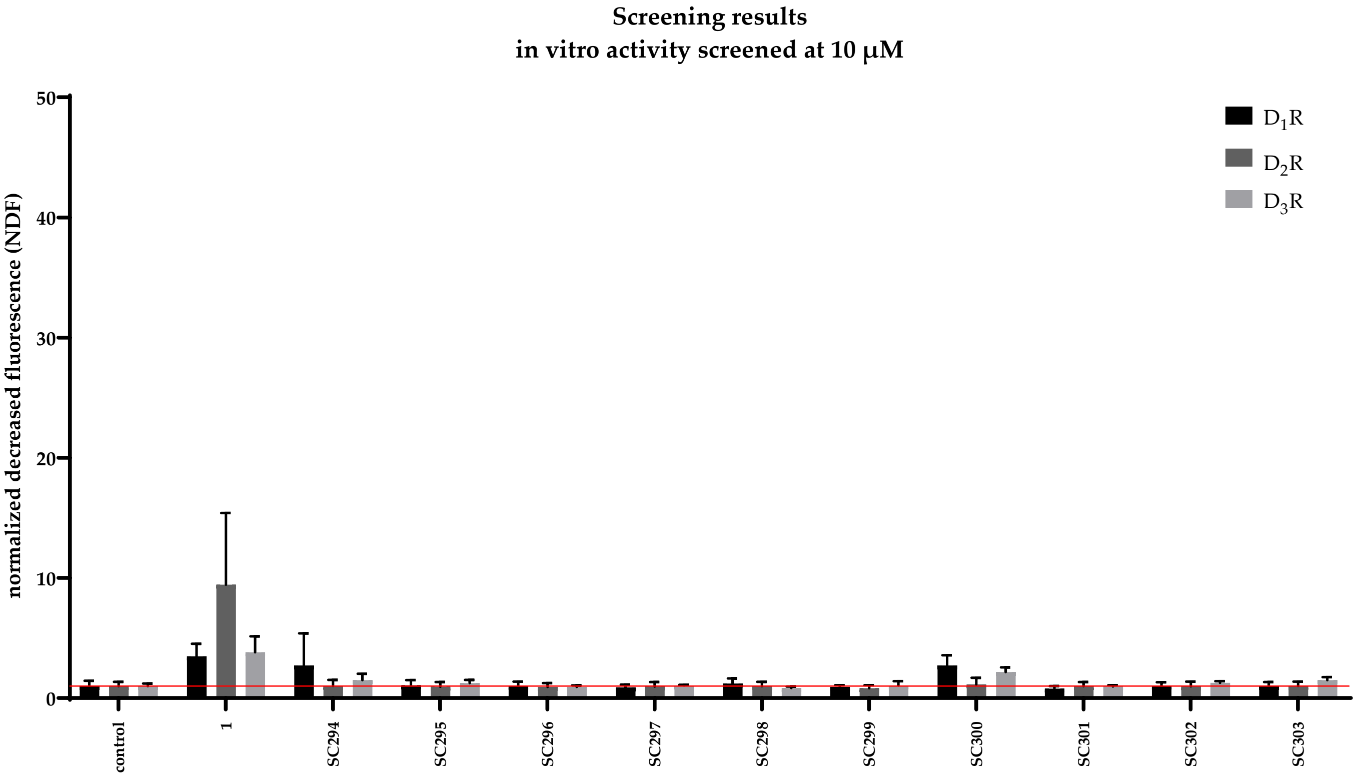

Figure S3. Comparison of biological activity of selected compounds at DR subtypes D_1R (black), D_2R (dark grey) and D_3R (light grey). Compound binding is shown as NDF in comparison to the control. Activities were measured in biological duplicates (two technical replicates each). Error bars show standard deviations (SD). The red line indicates a NDF of 1, thus, an inactive ligand at 10 µM screening concentration. NDF, normalized decreased fluorescence.

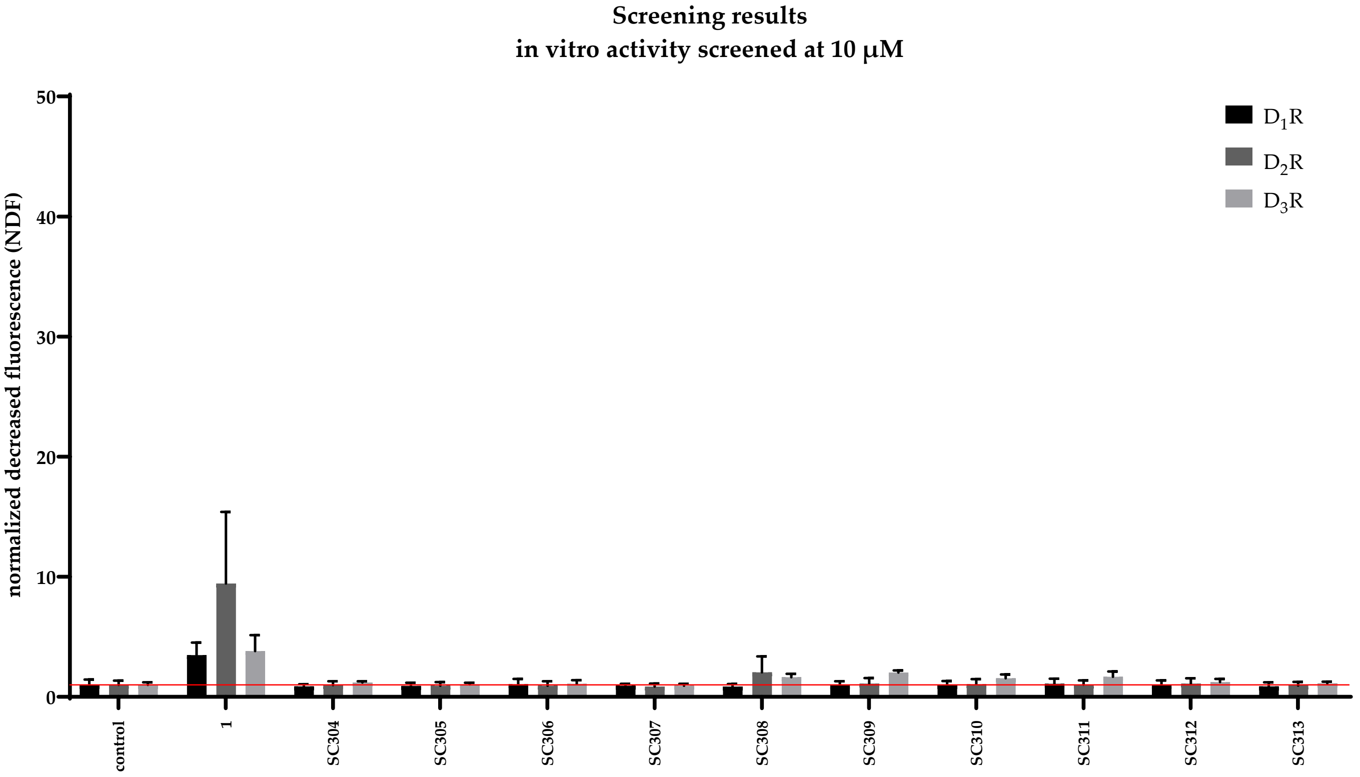

Figure S4. Comparison of biological activity of selected compounds at DR subtypes D_1R (black), D_2R (dark grey) and D_3R (light grey). Compound binding is shown as NDF in comparison to the control. Activities were measured in biological duplicates (two technical replicates each). Error bars show standard deviations (SD). The red line indicates a NDF of 1, thus, an inactive ligand at 10 µM screening concentration. NDF, normalized decreased fluorescence.

Screening results
in vitro activity screened at 10 µM

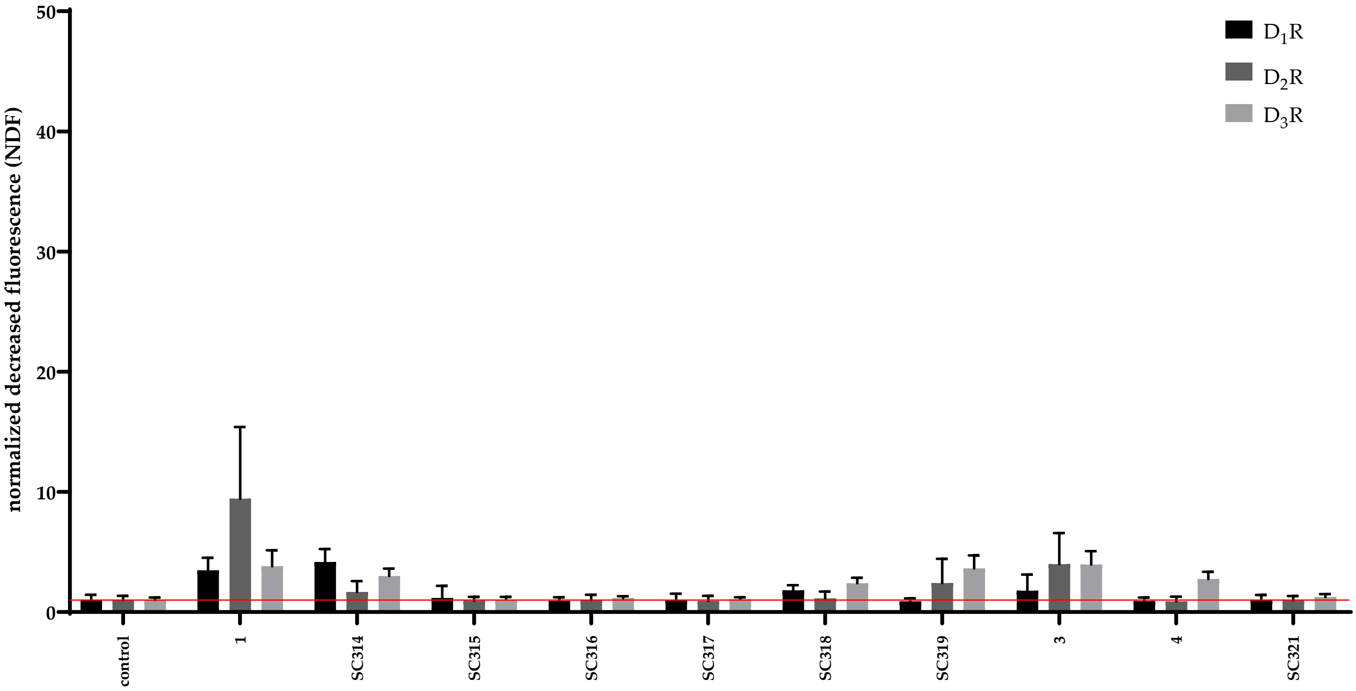

Figure S5. Comparison of biological activity of selected compounds at DR subtypes D_1R (black), D_2R (dark grey) and D_3R (light grey). Compound binding is shown as NDF in comparison to the control. Activities were measured in biological duplicates (two technical replicates each). Error bars show standard deviations (SD). The red line indicates a NDF of 1, thus, an inactive ligand at 10 µM screening concentration. NDF, normalized decreased fluorescence.

Screening results
in vitro activity screened at 10 µM

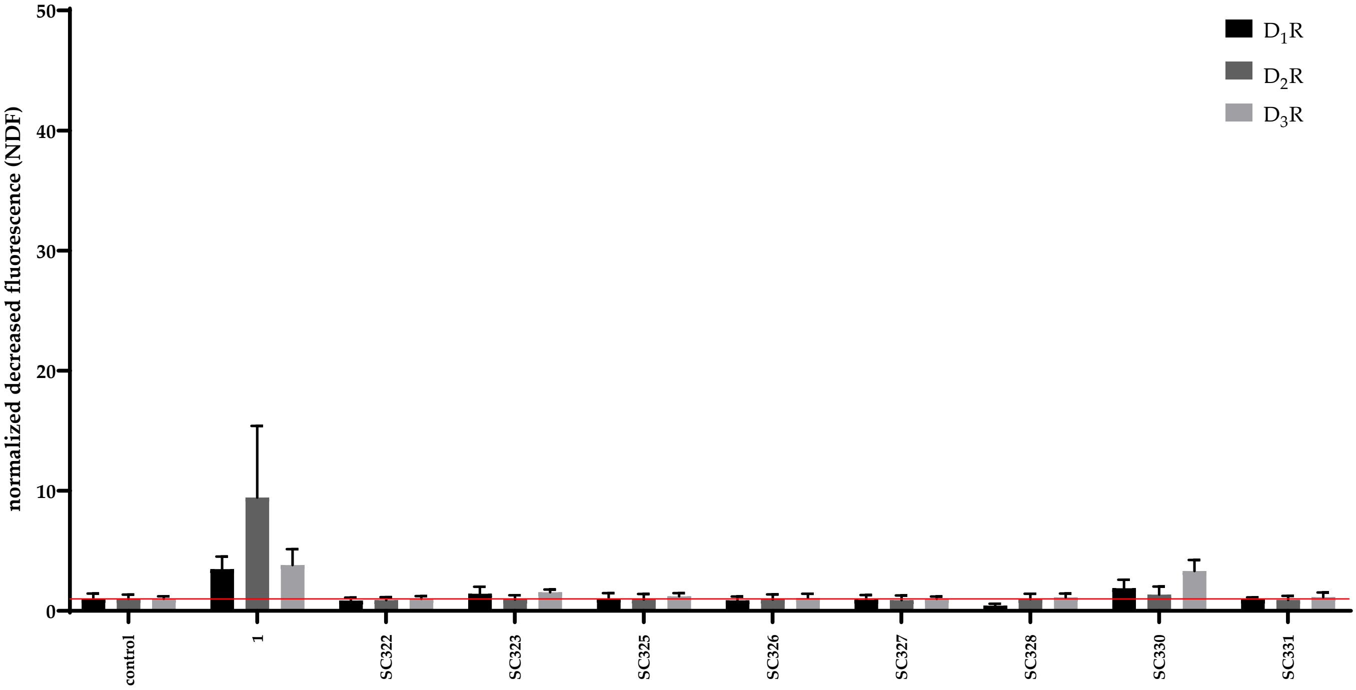

Figure S6. Comparison of biological activity of selected compounds at DR subtypes D_1R (black), D_2R (dark grey) and D_3R (light grey). Compound binding is shown as NDF in comparison to the control. Activities were measured in biological duplicates (two technical replicates each). Error bars show standard deviations (SD). The red line indicates a NDF of 1, thus, an inactive ligand at 10 µM screening concentration. NDF, normalized decreased fluorescence.

Screening results
in vitro activity screened at 10 µM

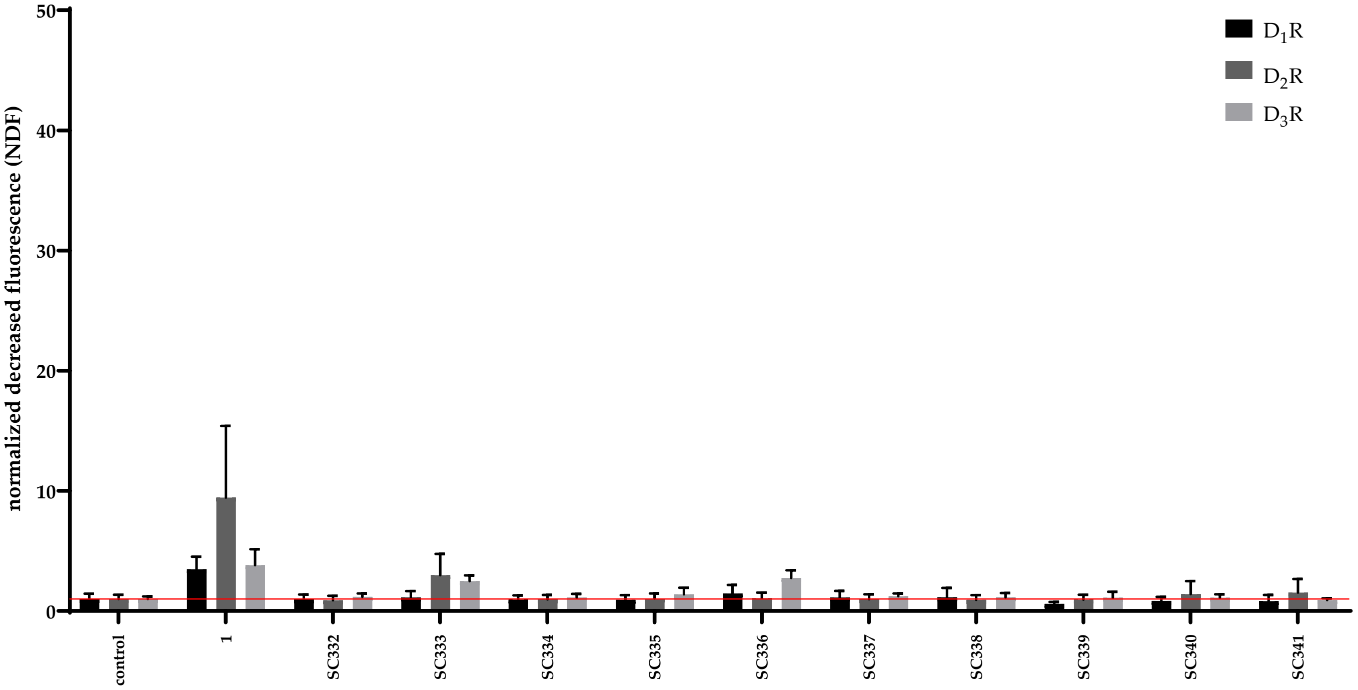

Figure S7. Comparison of biological activity of selected compounds at DR subtypes D_1R (black), D_2R (dark grey) and D_3R (light grey). Compound binding is shown as NDF in comparison to the control. Activities were measured in biological duplicates (two technical replicates each). Error bars show standard deviations (SD). The red line indicates a NDF of 1, thus, an inactive ligand at 10 µM screening concentration. NDF, normalized decreased fluorescence.

Screening results
in vitro activity screened at 10 µM

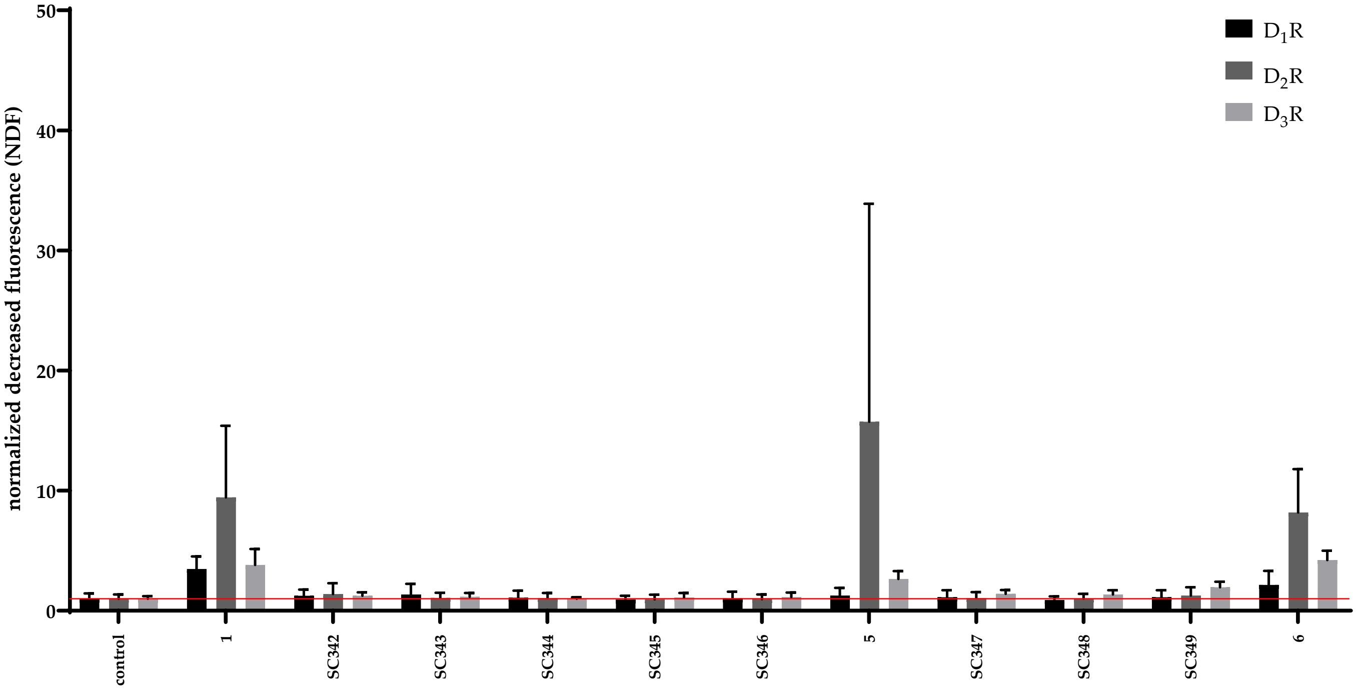

Figure S8. Comparison of biological activity of selected compounds at DR subtypes D_1R (black), D_2R (dark grey) and D_3R (light grey). Compound binding is shown as NDF in comparison to the control. Activities were measured in biological duplicates (two technical replicates each). Error bars show standard deviations (SD). The red line indicates a NDF of 1, thus, an inactive ligand at 10 µM screening concentration. NDF, normalized decreased fluorescence.

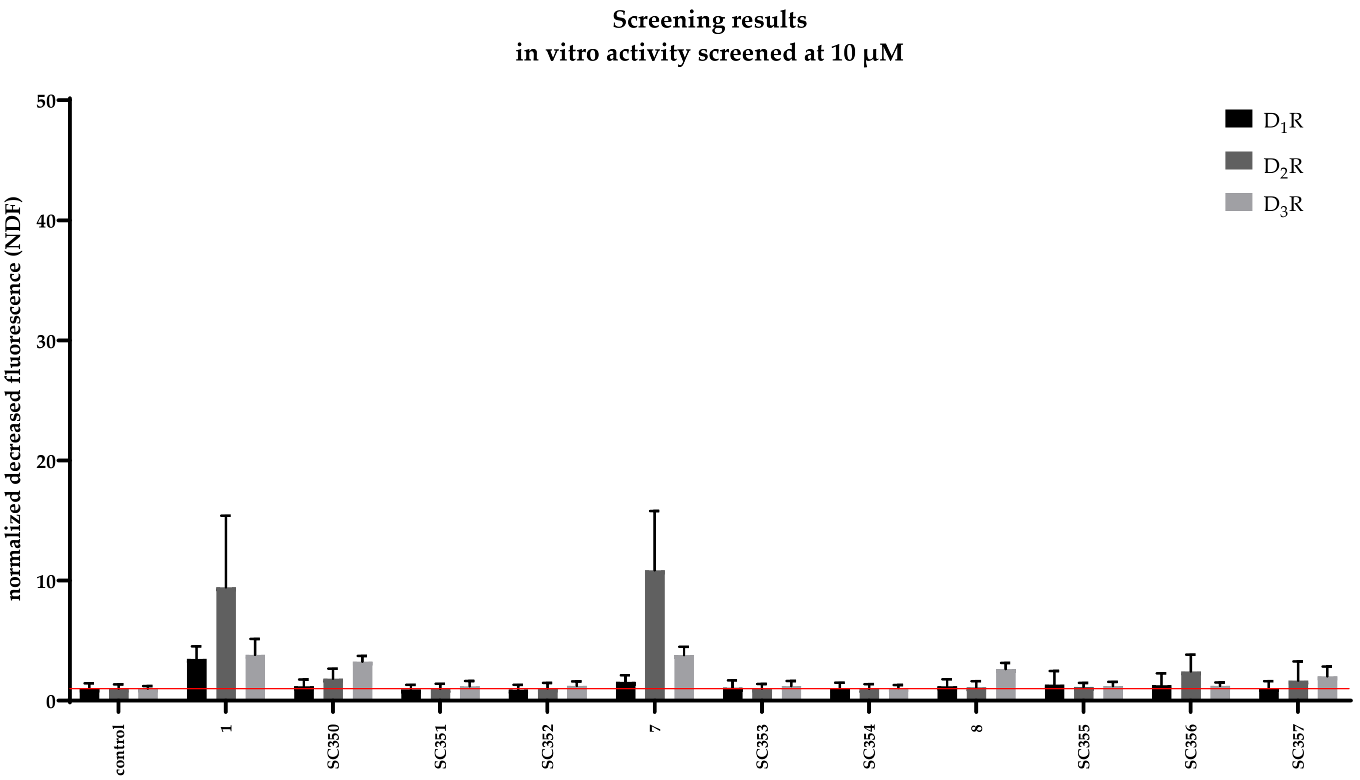

Figure S9. Comparison of biological activity of selected compounds at DR subtypes D_1R (black), D_2R (dark grey) and D_3R (light grey). Compound binding is shown as NDF in comparison to the control. Activities were measured in biological duplicates (two technical replicates each). Error bars show standard deviations (SD). The red line indicates a NDF of 1, thus, an inactive ligand at 10 μM screening concentration. NDF, normalized decreased fluorescence.

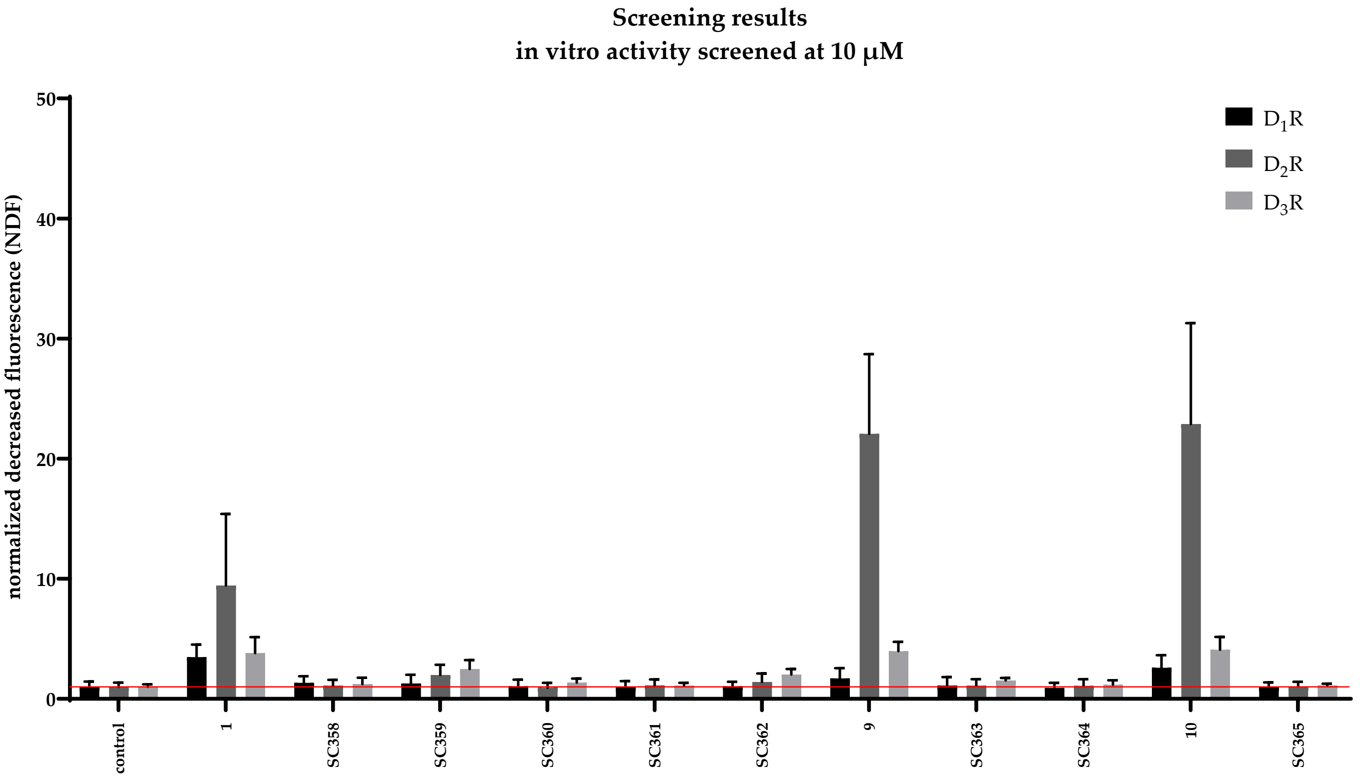

Figure S10. Comparison of biological activity of selected compounds at DR subtypes D_1R (black), D_2R (dark grey) and D_3R (light grey). Compound binding is shown as NDF in comparison to the control. Activities were measured in biological duplicates (two technical replicates each). Error bars show standard deviations (SD). The red line indicates a NDF of 1, thus, an inactive ligand at 10 μM screening concentration. NDF, normalized decreased fluorescence.

6.2. Summary of Ki values determined in vitro – overview of DR subtype selectivity

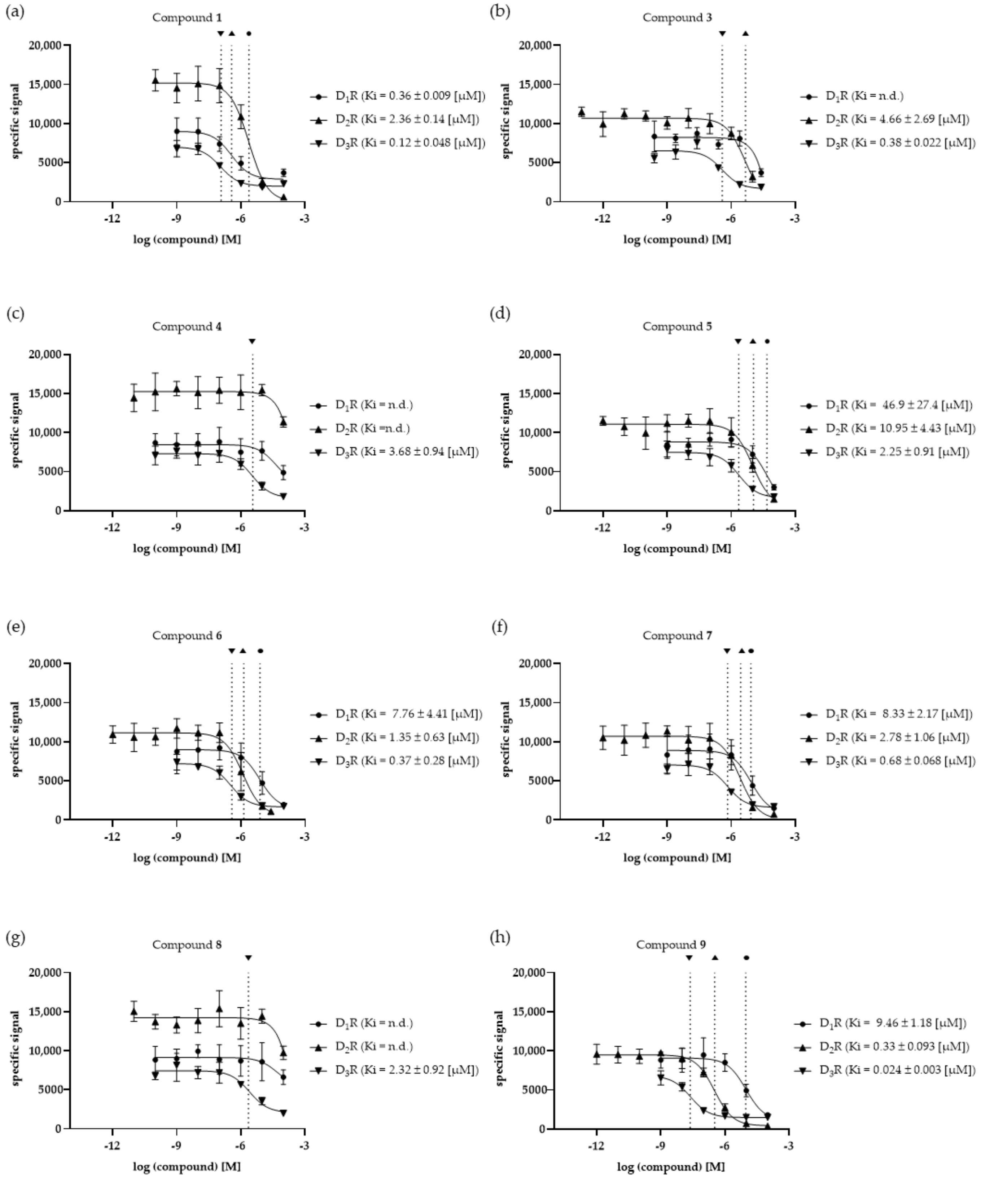

Figure S11. Comparison of the Ki values of compounds (a) **1**, (b) **3**, (c) **4**, (d) **5**, (e) **6**, (f) **7**, (g) **8** and (h) **9** determined at the investigated DR subtypes D1R, D2R and D3R. Vertical, dotted lines indicate the respective Ki values at the different DR subtypes. Ki values [µM] ± SD were determined with n = 6.

7. In silico insights into the DR binding pockets

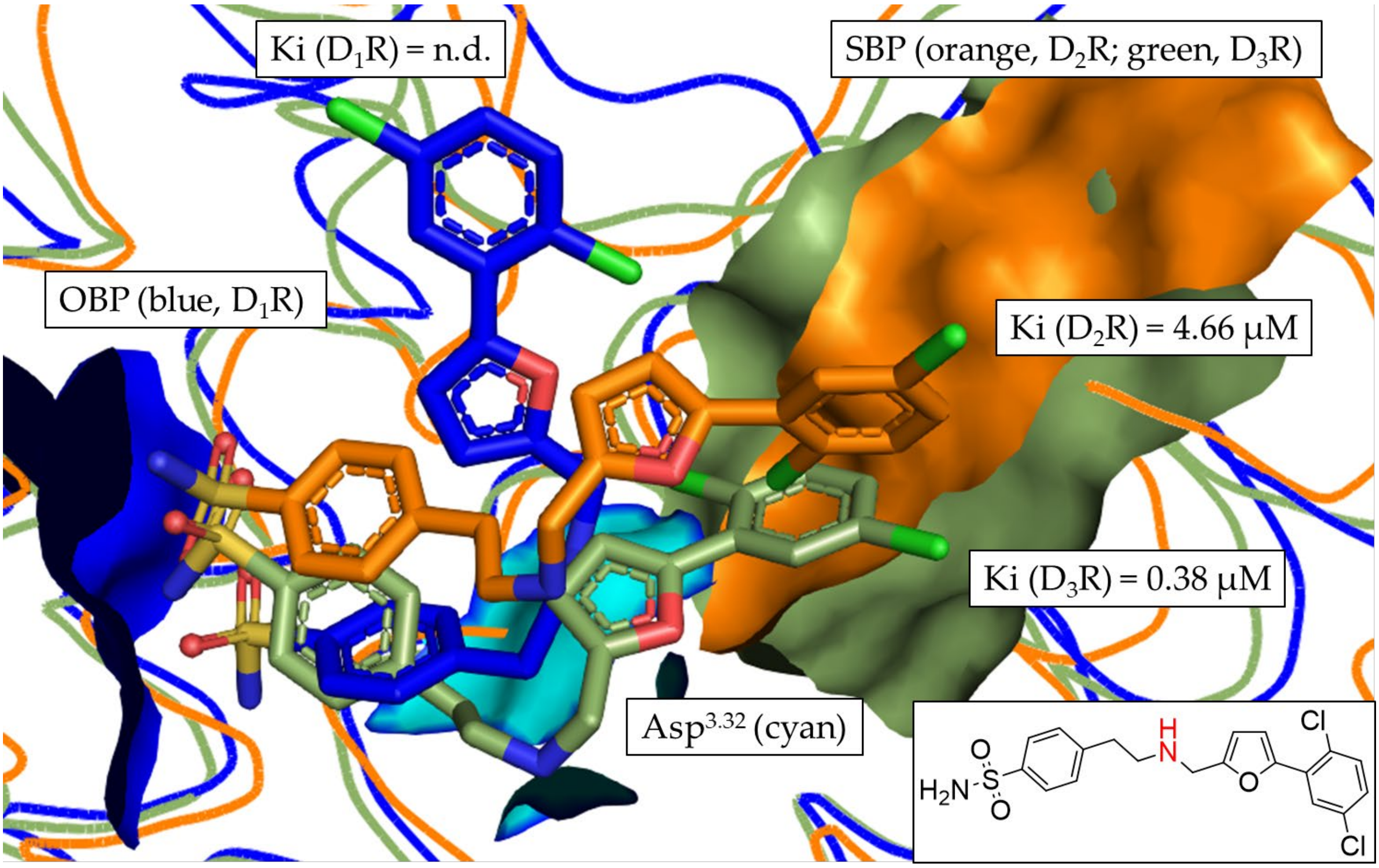

Figure S12. Alignment of the most frequent poses of compound **3** docked into D_1R, D_2R and D_3R. The surface of the highly conserved OBP is shown in blue (based on D_1R) consisting of $Asp^{3.32}$ (highlighted in cyan) and $Ser^{5.42/5.43/5.46}$. The conserved SBP-surface is displayed in orange (D_2R) and green (D_3R) consisting of $Val^{2.61}$, $Leu^{2.64}$, Gly^{ECL1}, $Phe^{3.28}$ and Cys^{ECL2} (individual amino acid labels shown in Table 3), respectively. Ki values determined in vitro are shown for each DR subtype. 2D structure of compound **3** is shown. Amine functional group involved in formation of the salt-bridge is highlighted in red.

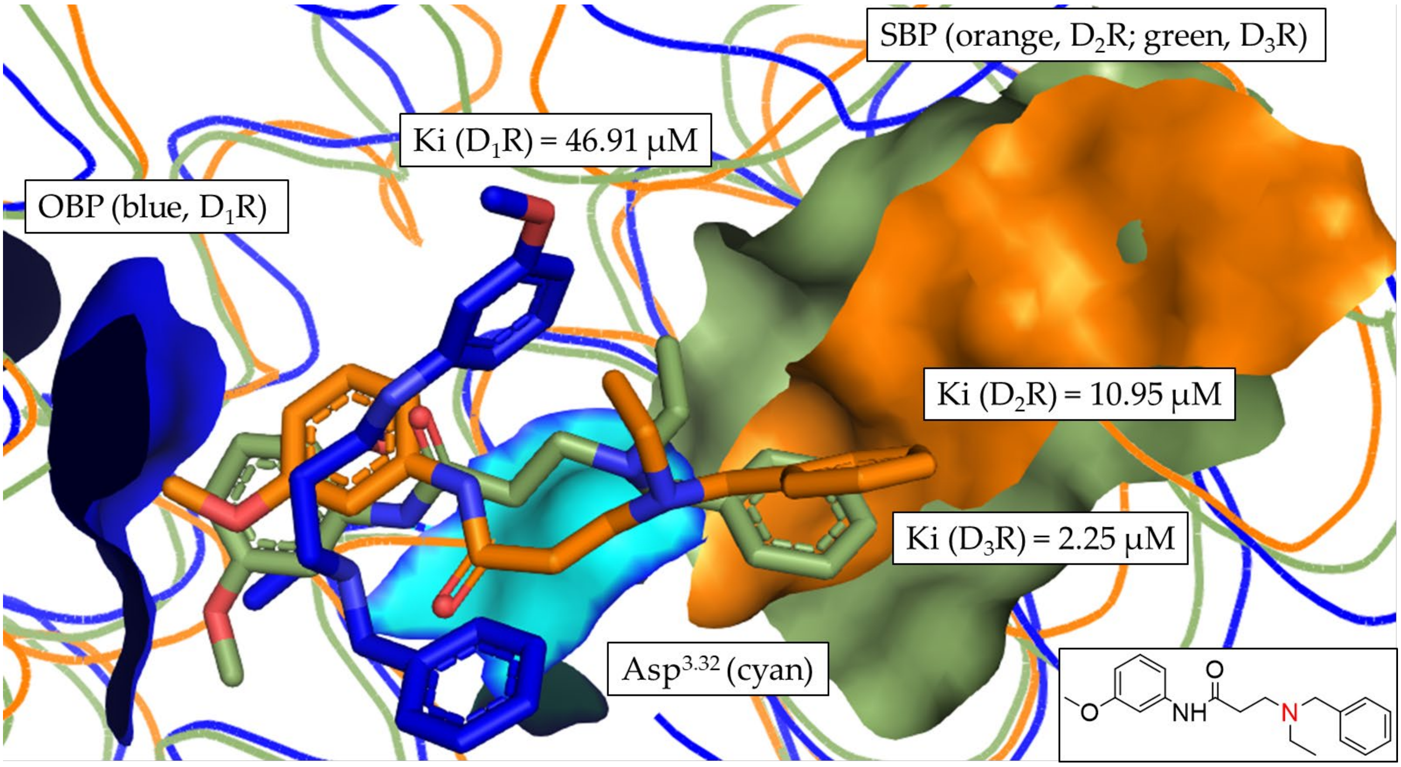

Figure S13. Alignment of the most frequent poses of compound **5** docked into D1R, D2R and D3R. The surface of the highly conserved OBP is shown in blue (based on D1R) consisting of Asp$^{3.32}$ (highlighted in cyan) and Ser$^{5.42/5.43/5.46}$. The conserved SBP-surface is displayed in orange (D2R) and green (D3R) consisting of Val$^{2.61}$, Leu$^{2.64}$, Gly^{ECL1}, Phe$^{3.28}$ and Cys^{ECL2} (individual amino acid labels shown in Table 3), respectively. Ki values determined in vitro are shown for each DR subtype. 2D structure of compound **5** is shown. Amine functional group involved in formation of the salt-bridge is highlighted in red.

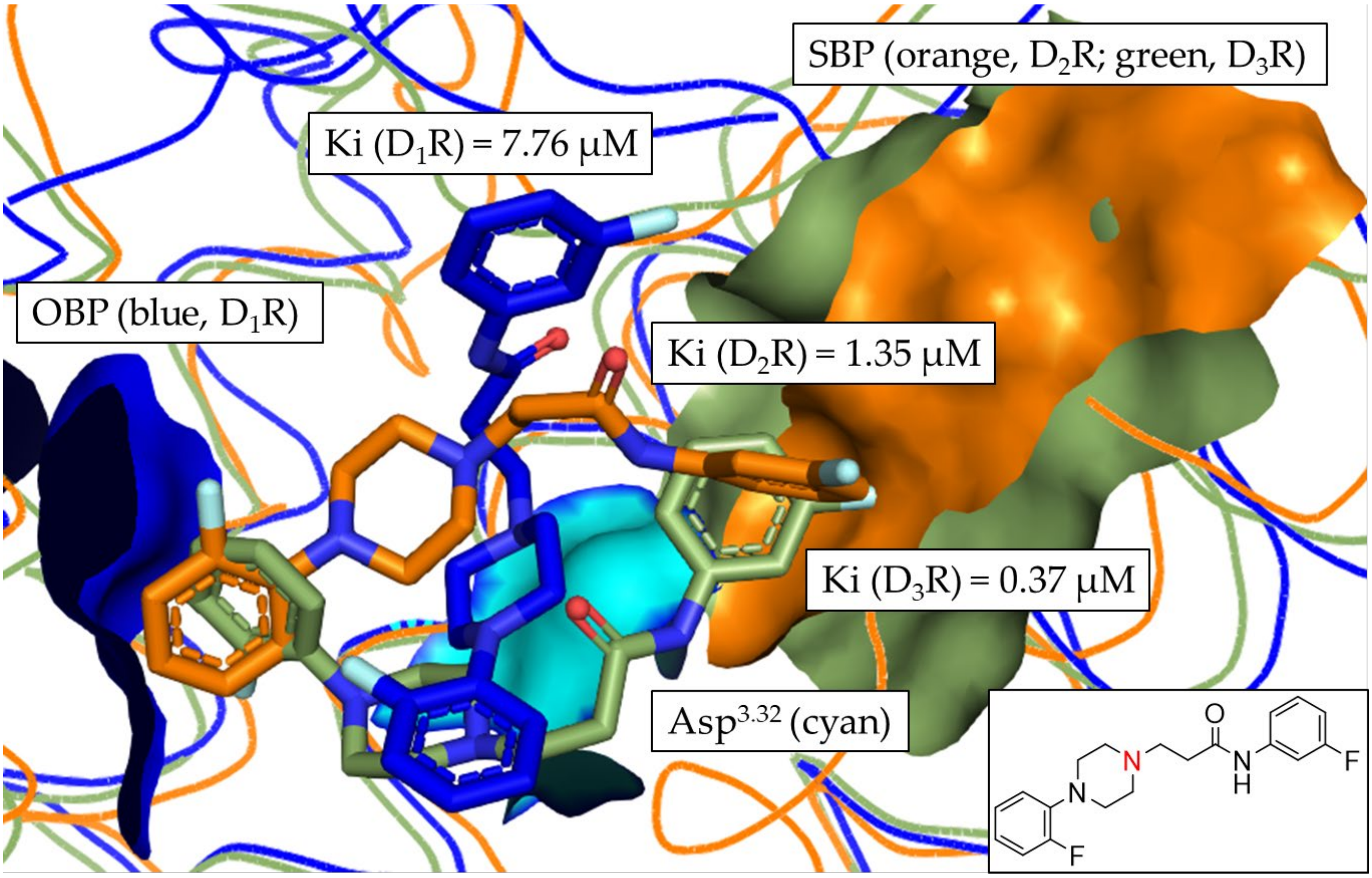

Figure S14. Alignment of the most frequent poses of compound **6** docked into D_1R, D_2R and D_3R. The surface of the highly conserved OBP is shown in blue (based on D_1R) consisting of $Asp^{3.32}$ (highlighted in cyan) and $Ser^{5.42/5.43/5.46}$. The conserved SBP-surface is displayed in orange (D_2R) and green (D_3R) consisting of $Val^{2.61}$, $Leu^{2.64}$, Gly^{ECL1}, $Phe^{3.28}$ and Cys^{ECL2} (individual amino acid labels shown in Table 3), respectively. Ki values determined in vitro are shown for each DR subtype. 2D structure of compound **6** is shown. Amine functional group involved in formation of the salt-bridge is highlighted in red.

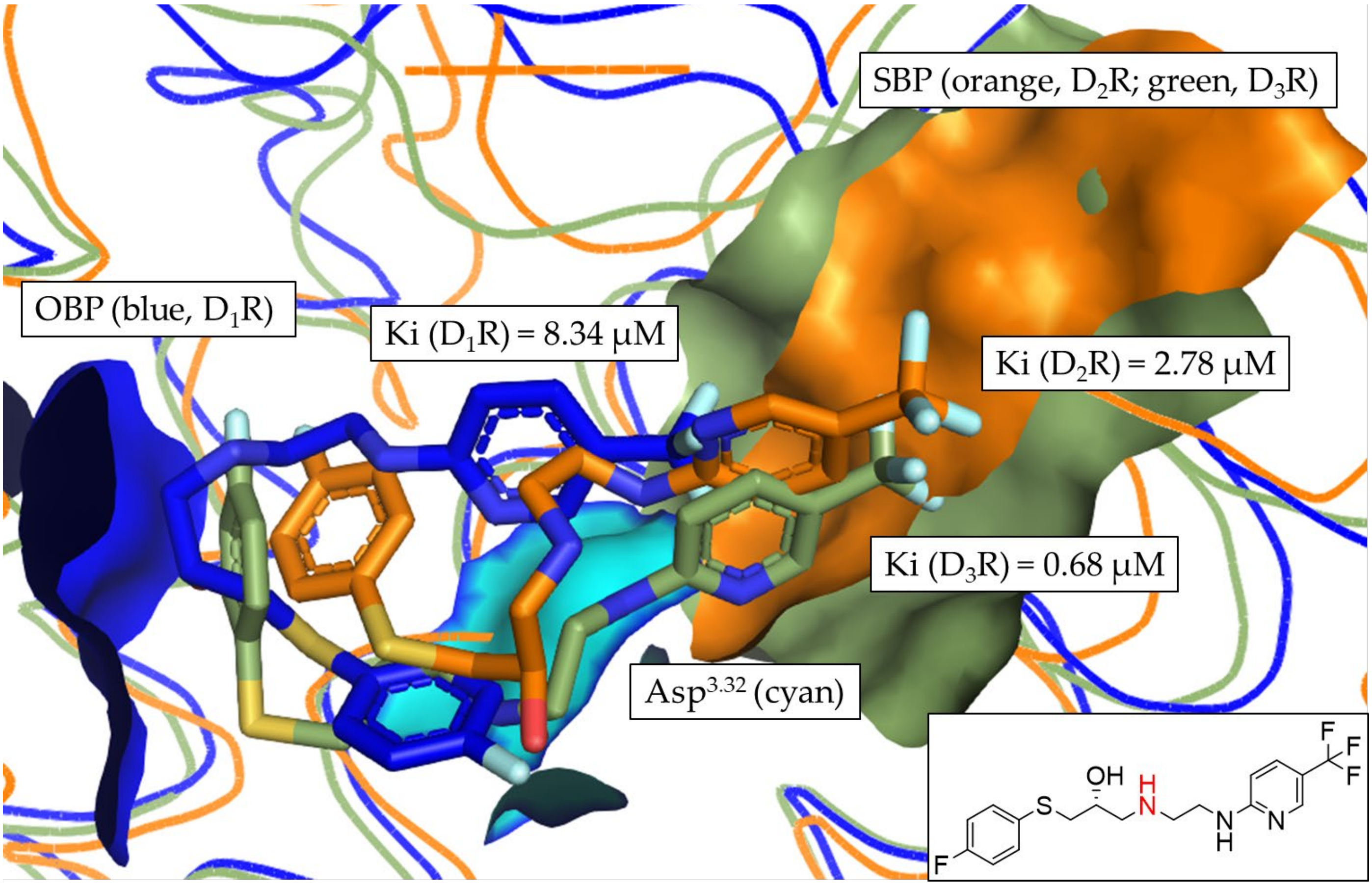

Figure S15. Alignment of the most frequent poses of compound **7** docked into D1R, D2R and D3R. The surface of the highly conserved OBP is shown in blue (based on D1R) consisting of $Asp^{3.32}$ (highlighted in cyan) and $Ser^{5.42/5.43/5.46}$. The conserved SBP-surface is displayed in orange (D2R) and green (D3R) consisting of $Val^{2.61}$, $Leu^{2.64}$, Gly^{ECL1}, $Phe^{3.28}$ and Cys^{ECL2} (individual amino acid labels shown in Table 3), respectively. Ki values determined in vitro are shown for each DR subtype. 2D structure of compound **7** is shown. Amine functional group involved in formation of the salt-bridge is highlighted in red.

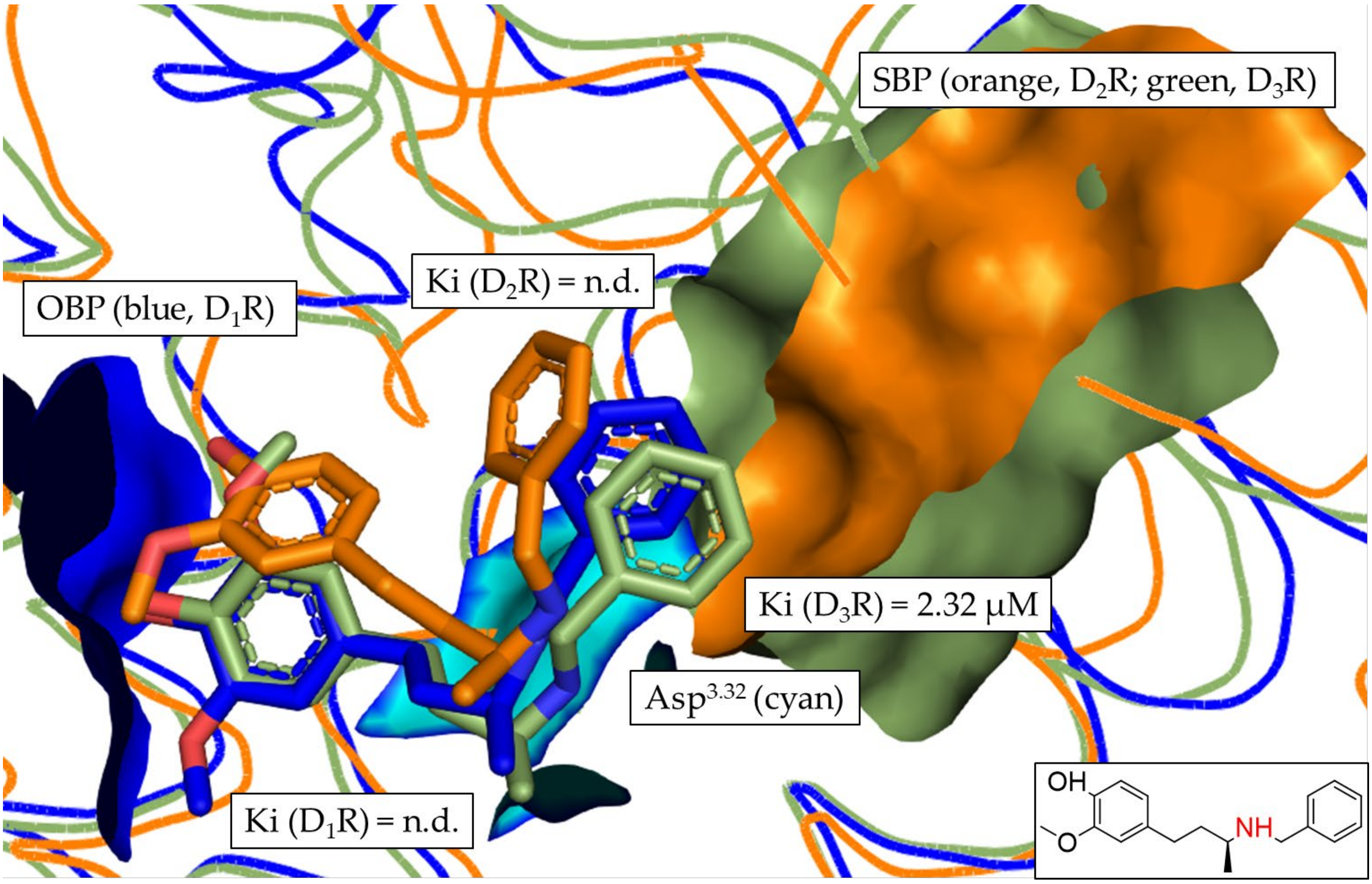

Figure S16. Alignment of the most frequent poses of compound **8** docked into D_1R, D_2R and D_3R. The surface of the highly conserved OBP is shown in blue (based on D_1R) consisting of $Asp^{3.32}$ (highlighted in cyan) and $Ser^{5.42/5.43/5.46}$. The conserved SBP-surface is displayed in orange (D_2R) and green (D_3R) consisting of $Val^{2.61}$, $Leu^{2.64}$, Gly^{ECL1}, $Phe^{3.28}$ and Cys^{ECL2} (individual amino acid labels shown in Table 3), respectively. Ki values determined in vitro are shown for each DR subtype. 2D structure of compound **8** is shown. Amine functional group involved in formation of the salt-bridge is highlighted in red.

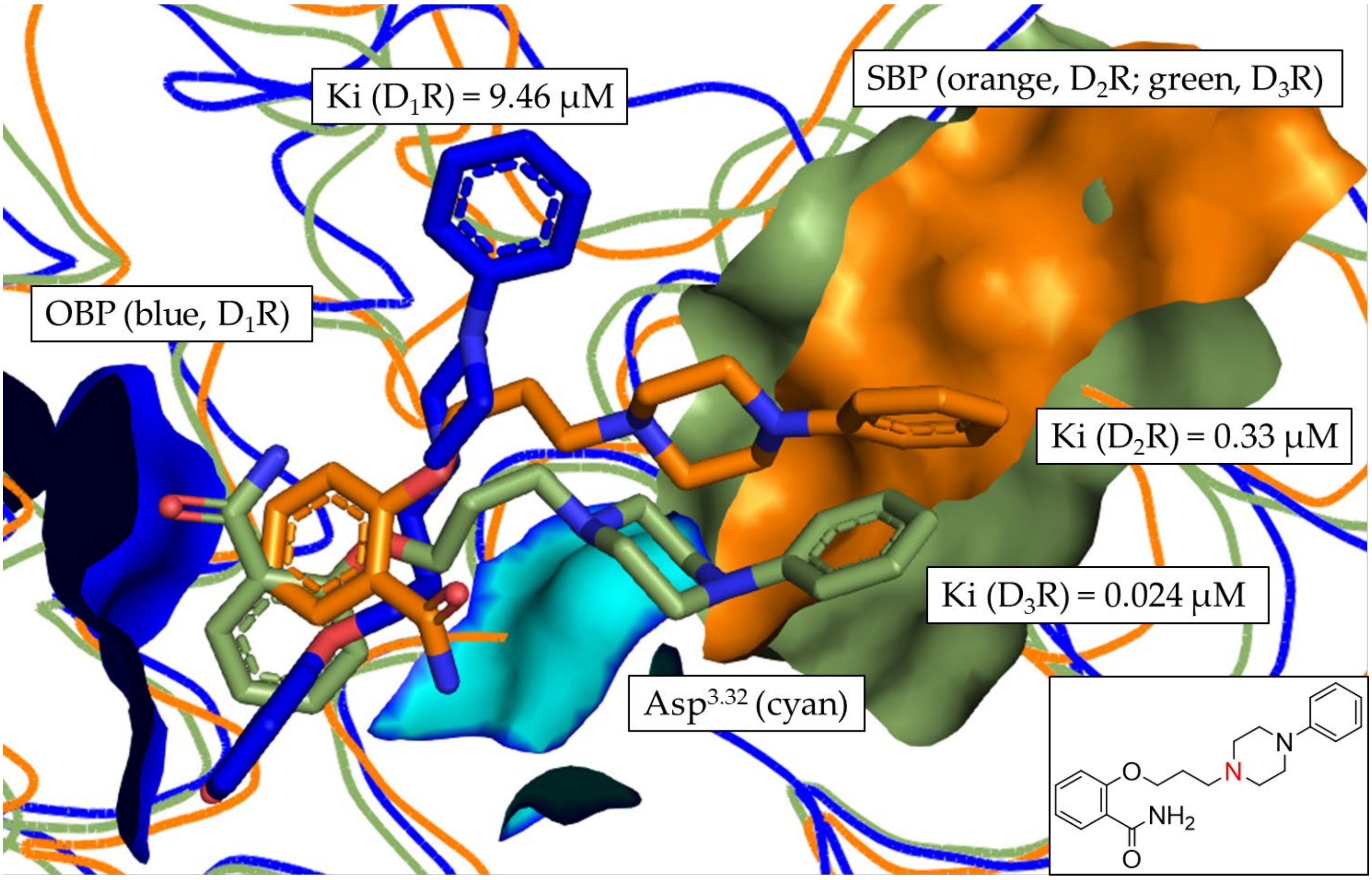

Figure S17. Alignment of the most frequent poses of compound **9** docked into D_1R, D_2R and D_3R. The surface of the highly conserved OBP is shown in blue (based on D_1R) consisting of $Asp^{3.32}$ (highlighted in cyan) and $Ser^{5.42/5.43/5.46}$. The conserved SBP-surface is displayed in orange (D_2R) and green (D_3R) consisting of $Val^{2.61}$, $Leu^{2.64}$, Gly^{ECL1}, $Phe^{3.28}$ and Cys^{ECL2} (individual amino acid labels shown in Table 3), respectively. Ki values determined in vitro are shown for each DR subtype. 2D structure of compound **9** is shown. Amine functional group involved in formation of the salt-bridge is highlighted in red.
